# Overwintering performance of bamboo leaves, and establishment of mathematical model for the distribution and introduction prediction of bamboos

**DOI:** 10.3389/fpls.2023.1255033

**Published:** 2023-09-08

**Authors:** Yufang Wu, Jing Li, Lixia Yu, Shuguang Wang, Zhuo Lv, Hao Long, Jingyu Zhai, Shuyan Lin, Yong Meng, Zhihua Cao, Hui Sun

**Affiliations:** ^1^ Faculty of Life Sciences, Southwest Forestry University, Kunming, China; ^2^ Faculty of Bamboo and Rattan, Southwest Forestry University, Kunming, China; ^3^ Key Laboratory for Forest Resources Conservation and Use in the Southwest Mountains of China, Ministry of Education, Southwest Forestry University, Kunming, China; ^4^ Horticulture Team, Beijing Zizhu Park, Beijing, China; ^5^ Bamboo Research Institute, Nanjing Forestry University, Nanjing, China; ^6^ Bamboo Research Institute, Hunan Academy of Forestry, Changsha, China; ^7^ Bamboo Research Institute, Anhui Academy of Forestry, Hefei, China

**Keywords:** bamboo leaves, overwintering performance, distribution area, low-temperature tolerance, mathematical prediction model, introduction

## Abstract

Bamboo has great economic values and is used extensively in many industries, and their natural distribution range was divided into 12 zones in China according to the temperature of their geographical distribution in previous works. Different bamboo species had significantly different abilities in low-temperature tolerance, which need to be considered carefully during ex-situ introduction. In this paper, we observed and evaluated the low-temperature damage of 19 bamboo species in winter, and measured the physiological changes of bamboo leaves. A total of 3060 leaf samples were obtained from 102 core collections in 34 bamboo species from the 5 regions of Chinese mainland for anatomical comparison, in order to screen out the key anatomical indicators related to their low-temperature tolerance and to establish a mathematical prediction model for bamboo introduction. The results showed that the low-temperature resistance of clustered bamboos was generally lower than that of the scattered bamboos. The decreased temperature led to the constant decrease of net photosynthetic rate and transpiration rate, but the increase of soluble sugar content in all bamboo species. There was no dormancy for all bamboo species in winter. The temperate bamboos showed lower photosynthesis as compared to tropical bamboos in winter. The leaf shape of bamboos was closely related to their distribution. A total of 13 leaf indicators were screened and more suitable to estimate the low-temperature tolerant abilities of bamboos and to predict their distribution. The MNLR (multiple nonlinear regression) mathematical model showed the highest fitting degree and the optimal prediction ability in the potential northernmost introduction range of bamboos. This study lay a foundation for bamboo introduction, and could also reduce the economic losses caused by the wrong introduction.

## Introduction

Bamboos provide wood and food for human life, economic and ecological benefits (Li et al., 2020). Bamboo was one of the world’s most important non-timber forest products. About 2.5 billion people around the world depend on bamboo economically, and international trade in bamboo exceeds $2.5 billion annually (Nirala et al., 2017). In tropical and subtropical regions, bamboo accounted for about 25% and 20% of the total biomass of all organisms, respectively. Bamboo not only had the unique advantage of a short rotation period, but also had better strength than several fast-growing plantation kinds of wood (Bansal and Zoolagud, 2002). Additionally, bamboos have a huge role in ecology and landscaping. The ecological functions of bamboo include erosion control and water conservation, moisture retention and rainfall interception, cleaning the air, reducing noise, etc. (Song et al., 2011). In the context of global climate change, finding low-cost ways to absorb carbon is becoming a major international policy objective (Montagnini and Nair, 2004). Bamboo grows rapidly and has a short growth cycle, which makes bamboo forests have huge carbon storage. Cultivating bamboo forests has become a natural and low-cost way to absorb carbon (Jyoti Nath and Das, 2012). Therefore, the existence of bamboo forests is conducive to mitigating climate change. The beautiful landscape of bamboo forests has aesthetic value and can also be used for urban greening and ecotourism (Wang et al., 2021). In general, bamboo is an important forest resource, which not only provides raw materials for economic development but also provides people with a variety of nutritious food and beautiful scenery. The development of the bamboo industry can also alleviate poverty and promote local economic development.

There are more than 590 bamboo species in China (Mera and Xu, 2014). China is not only the country with the largest number of bamboos in the world, but also the country with the most abundant bamboo resources and the largest bamboo forest area (Mera and Xu, 2014) Bamboo forests are mainly distributed in tropical and subtropical regions and are usually divided into 3 sub-vegetation types, named cool temperate bamboo forests, warm temperate bamboo forests, and hot bamboo forests (Liu et al., 2015; Xu et al., 2018). According to the underground stem, bamboo plants can be divided into subtype caespitose sympodium, subtype scattered sympodium, subtype mixed sympodium, monopodium, and amphipodium (Yi and Shi, 2008). Similarly, the distribution of bamboo in China can be divided into four zones, i.e., caespitose bamboos zone, caespitose and scattered mixed bamboo zone, scattered bamboo zone and subalpine bamboo zone according to the adaptive characteristics of different types of bamboos to climate, topography, and other ecological factors (Yi and Shi, 2008). Different types of bamboos have different adaptability to the new climate and environment. Usually, the species of *Dendrocalmus*, *Gigantochloa*, and *Cephalostachyum* need heat and humidity to grow well, while those of *Phyllostachys*, *Bashania*,and *Fargesia* are more cold tolerant. Most bamboo species with weak low-temperature tolerance, recent research has found that temperature was a main limiting factor for bamboo distributions (Li et al., 2019). Therefore, bamboos are mainly distributed in the southern area and only a few in the north area of China (Tong, 2007).

The large-scale introduction of bamboos occurred in the 1970s, and China launched the ‘Transferring Bamboo from South to North’ project (Liu et al., 2015). However, most bamboo introductions failed, because few works were made on the bamboo species screening, introduction test, cultivation and domestication before introduction (Su, 2008). As a result, most of the successfully introduced bamboos were scattered bamboos such as Phyllostachys. The main reason for the death of most bamboo species was the poor adaptability to the cold and drought climate in the north, so they grew badly and died finally (Sun et al., 2002). The ‘Transferring Bamboo from South to North’ project laid a certain foundation for the screening of cold tolerant bamboo species in China (Liu et al., 2013).

In addition to the bamboo introduction in China, there was also a long history of introducing bamboo from China to abroad. As a neighbor of China, the Japanese introduced moso bamboo from China as early as 1736 (Zhu and Li, 1994), which has now developed into the main bamboo species in Japan and was widely distributed throughout the country (Tanaka et al., 2014). European and American countries such as France, the former Soviet Union, the United States, and other countries also had a history of introducing bamboo from China. Although bamboos are not endemic species in Europe, they have been an important part of ornamental horticulture and landscaping since they were introduced (Gielis, 2002).

Since the climatic conditions in US was similar to those in China, most of the introduced bamboo species survived in the east and west coasts (McClure, 1945). However, there were also cases of failure. According to the recorded data of the *Inventory of Seeds and Plants Imported* of USDA and *Plantae Wilsonianae* bamboo species and the existing bamboo species data of American Bamboo Society (ABS), the introduction of the following bamboo species failed including *Acidosasa nanunica*, *A. venusta, Dendrocalamus farinosu, Indocalamus herklotsii, I. sinicus, I. wilsoni, Oligostachyum gracilipes, Pseudostachyum polymorphum, Sasa longiligulat, Schizostachyum funghomii, S. pseudolima, Shibataea kumasasa, Sinobambusa humilis* and *Si. Rubroligula* (Wu et al., 2020).

In China, the atmosphere temperature was inversely related to latitude and the temperature fell as latitude increased, and vice versa. The low temperature was the main reason for the introduced bamboos that could not safely overwinter and died finally. Cold tolerance was the ability of plants to endure seasonal low but non-freezing temperatures (0-15 C) while freezing tolerance indicated the ability of plants to survive at subzero (< 0 C) temperatures (Adhikari et al., 2022). Most tropical bamboo species suffered severe damage at temperatures below 0 C, such as the bamboo species of *Dendrocalamus*.

**Figure 1:**
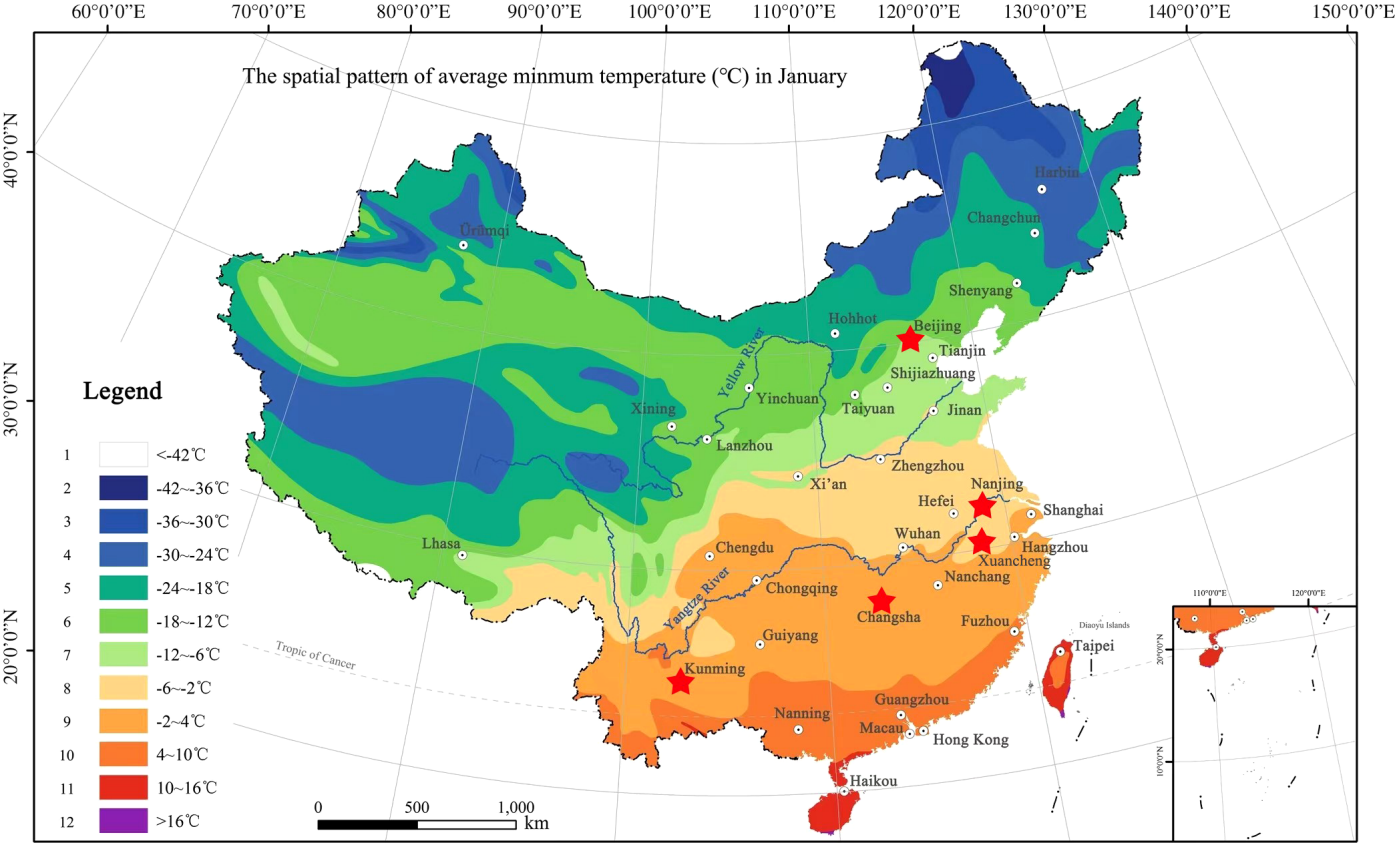
The hardiness zoning map of Bambusoideae in China (Shi et al., 2021), which were divided into 12 zones. Stars indicated the sampling sites include Kunming City, (N25°06’, E102°78’), Changsha City (N28°12’, E112°59’), Xuancheng City (N30°57’, E118°45’), Nanjing City (N32°04’, E118°48’) and Beijing City (N39°56’, E116°20’).

In low-temperature environment, the net photosynthetic rates and transpiration rates decreased significantly (Repkina et al., 2021), but the soluble sugar content increased so as to resist the adverse effects of low temperature (Lin et al., 2019). If the temperature continued to decrease, it would cause irreversible damage to plant cells and even plant death (Guy, 2003; Lu et al., 2022). The screening of suitable bamboo species with low temperature tolerant ability had become a key factor in the bamboo introduction and management. As for the bamboo distribution in China, Yi and Shi (2008) proposed a standard map of climate adaptability division for the distribution range of bamboos according to the lowest temperature they could tolerate in January. In this map, China was divided into 12 planting zones, with the lowest temperature in zone 1 and the highest temperature in zone 12 (**Figure 1**) in winter. The farther north bamboos are distributed in, the higher their tolerance to low temperature. The temperate bamboos distribute in the low number zones has higher abilities in low temperature tolerance.

Although Yi and Shi (2008) have made much work on the distribution range of bamboos, but there are still a large number of bamboo species that were unidentified in their distribution range. Meanwhile, we still can not predict the northernmost edge where the bamboos could be introduced and survived. To date, only a few bamboo species have been screened out to plant in the low temperature zones. Moreover, the large scale introduction tests will also consume a large amount of manpower, materials and financial resources. Meanwhile, the distribution range of those bamboo species introduced from abroad was also unclear in China. Hence, it is essential to establish a mathematical model for the distribution prediction of bamboo by screening the stable key indicators.

For plants, photosynthesis is easily affected by environmental conditions (Lou et al., 2018). Moreover, plants responded to environmental changes by implementing a series of physiological, metabolic, and developmental changes (Misyura et al., 2013). Leaves are not only the most important photosynthetic organ but also the most sensitive part to environmental changes (Gong et al., 2020). Leaf structure and morphological characteristics regulate the relationship between leaves and temperature, which can determine the temperature range experienced and directly reflect the long-term adaptability of plants to the ecological environment (Sastry and Barua, 2017). Zhang et al. (2007) found that the deposition of thick cuticle on the transgenic Arabidopsis leaves increases their frost resistance. Zeng et al. (2020) noticed that both adaxial epidermis thickness and stomatal density are important indicators of the low-temperature tolerance of *Camellia oleifera*. Photosynthetic and physiological changes are largely a reflection of plant stress to the outside world, and morphological structure was the evolutionary result of plants adapting to the environment. Therefore, the physiological and biochemical characteristics of leaves were more sensitive to environmental changes than the stable morphological characteristics (Li et al., 2014). In summary, we try to screen out the stable indicators significantly related to low-temperature tolerance among leaf morphological and anatomical characteristics as the key indicators for the establishment of the mathematical prediction model for the bamboo introduction. Additionally, regional and seasonal changes might also have a certain impact on the indicator screening. Hence, it was necessary to select the stable evaluating indicators and alleviated the seasonal and regional influences, so as to ensure the accuracy and applicability of the mathematical prediction model.

## Materials and methods

### Temperature and light intensity in winter of Kunming City

January was the coldest month in Kunming City, and the temperature decreased month by month from November to January of the next year (**Table 1**). Therefore, we took the months as the time to carry out the low-temperature tolerance research.

**Table 1:** Temperature and precipitation at sampling sites (2001-2020).

Location	Latitude	Month	Average high temperature (°C)	Average low temperature (°C)	Extreme high temperature (°C)	Extreme low temperature (°C)	Average precipitation (mm)
Kunming	N25°02'	Nov	20	8	24	2	37
		Dec	17	4	21	0	14
		Jan	16	3	23	-2	13
		Jun	26	18	30	12	180
		Jul	25	18	29	14	211
		Aug	26	18	31	14	200
Changsha	N28°12'	Nov	17	11	30	0	73
		Dec	12	5	21	-6	45
		Jan	10	4	25	-5	61
Xuancheng	N30°57'	Nov	17	8	28	-3	69
		Dec	11	2	19	-7	42
		Jan	9	0	23	-14	69
Nanjing	N32°04'	Nov	16	8	25	-4	52
		Dec	10	2	18	-6	29
		Jan	8	0	22	-9	39
Beijing	N39°56'	Nov	10	1	24	-9	8
		Dec	4	-5	28	-19	2
		Jan	3	-6	24	-18	3

In addition, we also collected the samples from four other different sites in China, i.e., Changsha City (N28°12’, E112°59’), Hunan Province, Xuancheng City (N30°57’, E118°45’), Anhui Province, Nanjing City (N32°04’, E118°48’), Jiangsu Province, and Beijing City (N39°56’, E116°20’) (**Figure 1**). According to the average month temperature of each city in the past two decades (2001-2020), the coldest month of all these cities was January (**Table 1**). The diurnal and monthly variations of net photosynthetic rate and transpiration rate of different bamboo species were explored, rather than the differences in net photosynthetic rate and transpiration rate of bamboo between different cities, so the photosynthetic active radiation intensity of Kunming City was only concerned. The diurnal variation of light intensity showed a similar trend in the three months of Kunming City with a single peak curve (**Figure S1**).

### Plant materials

The morphological and anatomical characteristics of leaves were easily affected by the environmental conditions. Hence, it was important to screen out the stable key indicators that could effectively determine the distribution range of bamboo from all the leaf characteristics.

**Figure 2:**
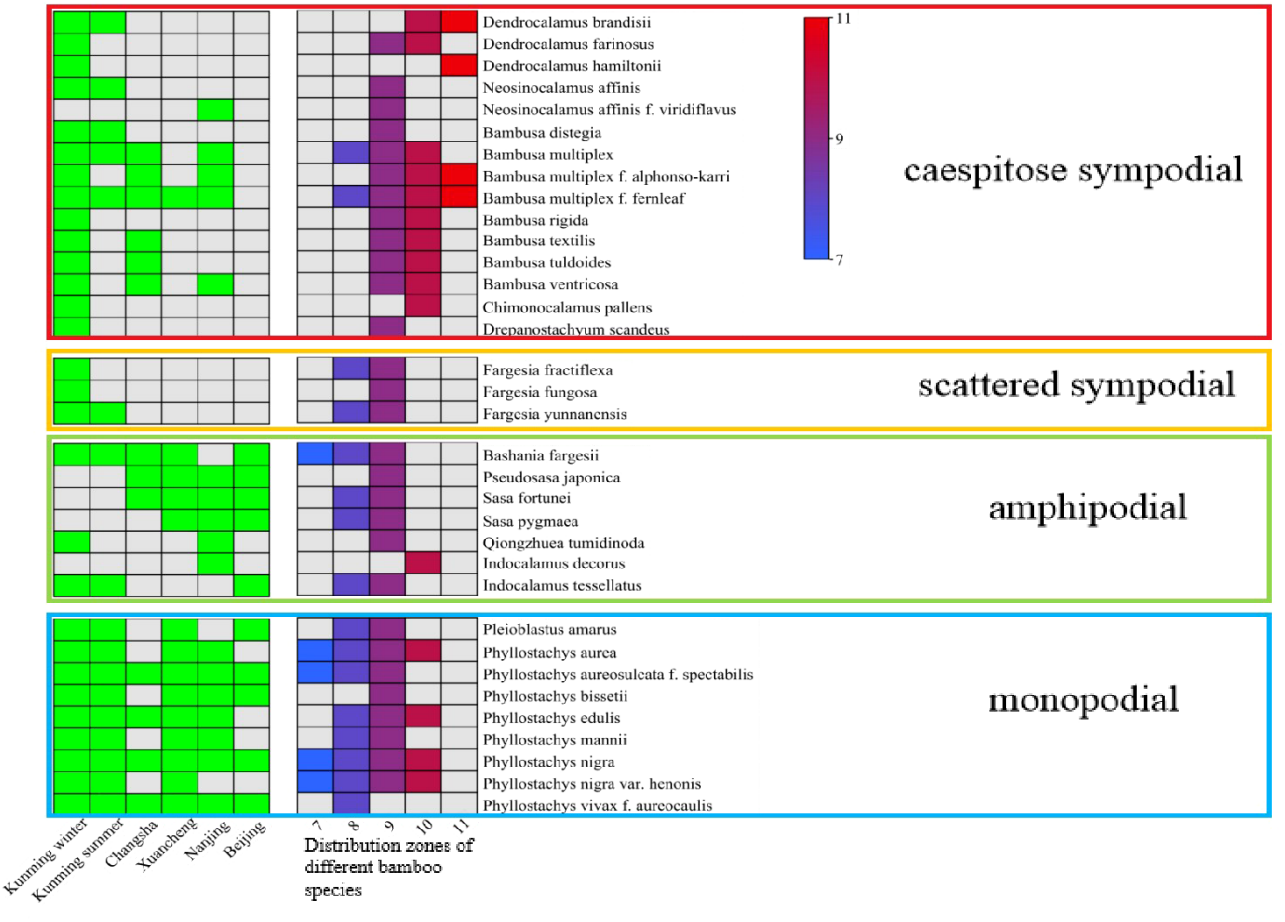
The collection city of each bamboo species and the distribution zones of bamboos. The green square indicate the collected bamboo species. Squares from blue to red indicate the distribution of bamboo from low temperature to tropical regions.

In this study, a total of 34 bamboo species, 102 core collections, and 3060 samples were collected from five cities in China, which could reveal the bamboo distribution from southern to northern in China (**Figure 2**). To improve the accuracy of correlation analysis and eliminate the influences of regions on leaf characteristics, the leaf samples of 29 bamboo species were chosen in the bamboo garden of Southwest Forestry University in Kunming City (N25°06’, E102°78’). Kunming City suffered a significant drop in temperature in winter of 2019, so we collected these samples during this period. In order to eliminate the influences of seasons and to screen out the stable indicators, the leaves of 18 bamboo species were repeatedly sampled in both summer and winter in the bamboo garden of the Southwest Forestry University.

The bamboo species were not completely the same in different regions of China. Hence, it was difficult to obtain the leaf samples from a large number bamboo species that were common in all regions of China. The leaf samples of the same bamboo species from other regions were collected to analyze the variation degrees and further to eliminate the influences of regions during model establishment. However, the leaf samples of some other bamboo species that were not the same in the regions were also collected to verify the accuracy of mathematical models. The leaves of a total of 13 bamboo species were sampled in the bamboo garden Changsha City (N28°12’, E112°59’), 15 bamboo species in the bamboo garden of Xuancheng City (N30°57’, E118°45’), 17 bamboo species in the bamboo garden of Nanjing Forestry University in Nanjing City (N32°04’, E118°48’) and 10 bamboo species in the Zizhu Park in Beijing City (N39°56’, E116°20’) (**Figures 1**, **2**). This sampling proposal was basically in accord with the bamboo distribution from the southern to the northern of the Chinese mainland.

The leaves of 2-year-old bamboo were chosen as samples. This was mainly because the 2-year-old bamboos were in their mature stage and the leaves were the main organ that easily suffered the freezing injury. Three culms of each bamboo species were selected and 20 g leaves were sampled around the middle of the canopy.

### Observations on the freezing injury of bamboo leaves in winter

The freezing injury of bamboo leaves were observed from November to January in 2019 and 2020, and the damage of leaves during winter seasons were recorded (‘+’ indicated no significant damage in winter or slight yellowing of leaf tips. ‘++’ indicated slightly cold damage, and 25% of the leaf area became yellow. ‘+++’ indicated severe cold damage, and 50% of the leaf area became yellow). The low-temperature tolerant abilities of bamboos were evaluated and divided into three grades (level 1: strong low-temperature tolerance, level 2: middle low-temperature tolerance, level 3: weak cold tolerance).

### Determination of photosynthesis, moisture, and photoassimilates content

The photosynthetic and transpiration rates were measured by using the Top Cloud-Agri 3051D portable photosynthetic instrument (Zhejiang Top Cloud-Agri Technology Co., Ltd., Hangzhou, China). During the period from November to January in Kunming City, a total of 30 leaves were determined for each bamboo species, and each leaf was measured once an hour from 8: 00 to 18: 00 and repeated three times. The average value of the photosynthetic rate measured from 8: 00 to 18: 00 every day in each month was taken as the net photosynthetic rate of the month.

For moisture content determination, approximately 2.0 g of samples were weighed and dried at 105°C. The moisture contents were calculated according to the weight difference before and after drying.

The extraction of endogenous soluble sugar and starch contents was performed according to the method of Glassop et al. (2007). A 1.0 g leaf sample was ground in liquid nitrogen, extracted with 10 mL deionized water at 70°C, and centrifuged at 12,000 g for 20 min. The collected supernatant was used to determine soluble sugar content. The sediments were stored at -20°C for starch content determination. The contents of endogenous soluble sugar and starch were determined by the phenol-sulfuric acid method (Dubois et al., 1956). Each sample was measured three times.

### Morphological characteristics of leaves between different species, seasons, and sites

A total of 3060 leaves collected from five cities were scanned by a scanner to generate the leaf images. The leaf length, leaf width, and leaf area were measured by a two-dimensional measurement software (DS-3000, Caikang, Shanghai, China) and the leaf length/leaf width ratio was calculated.

The leaves were placed under a stereomicroscope to count the number of veins and to calculate the vein density as the following formula:


Vein density=amount of weinsleaf width (cm)

The stomata density was measured in the middle parts of the leaf samples under a fluorescence microscope (Nikon E400, Nikon, Tokyo, Japan) and photographed with the two-dimensional measurement software according to the following formula:


Stomata density=amount of stomatalsleaf area(mm2)

### Measurement of leaf anatomical structures

A total of 3060 leaves of 102 collections were cut into small pieces and fixed in FAA, and then were cut into 7μm thick paraffin sections by a rotary cutting machine (Leica RM2165, Leica, Frankfurt, Germany). The sections were observed and photographed under a light microscope (PH100-3B41L-IPL, Phenix, Jiangxi, China). The leaf thickness and the thickness of the adaxial epidermis, abaxial epidermis, adaxial cuticle, abaxial cuticle and mesophyll were measured by using the two-dimensional measurement software.

### Establishment of mathematical model for bamboo distribution prediction

All bamboo species had their northernmost distribution zones, which were often determined by their low-temperature tolerance. According to the description of *Illustrated Flora of Bambusoideae in China* (Yi and Shi, 2008), the farther north the distribution area was, the smaller the distribution zone was. The morphological and anatomical characteristics of bamboo leaves were easily affected by seasons and regions, resulting in a decline in the accuracy of correlation analysis. Hence, the northernmost distribution zones of all bamboo species distributed in Kunming City were chosen to analyze their correlations with the morphological and anatomical characteristics of their leaves in winter seasons, so as to screen out the indicators that correlated significantly with their distributions (P<0.05). It could effectively reduce the influences of regions on the key indicators screening by analyzing the bamboo species in the same environment.

To analyze the influences of seasons on the indicators, the seasonal variation degree (between summer and winter) of all indicators of 18 bamboo species obtained in Kunming City was calculated as the following formula:


Coefficient of variation (CV)=standard deviation (SD)mean (M)×100%

To analyze the influences of regions on the indicators, three bamboo species in all 5 cities (Changsha City, Nanjing City, Xuancheng City, Beijing City, and Kunming City) were chosen to analyze their regional variation. The mathematical models were established based on the indicators with weak and middle variation degrees (CV<35%). All those with high variation degrees (CV>35%) were rejected during the models establishment.

All the selected indicators were chosen as independent variables to establish the prediction models of bamboo together with their northernmost distribution zones that were set as dependent variables.

#### (1) Multiple linear regression (MLR)

The multiple linear regression formula was established with the selected indicators and the northernmost distribution zone of each bamboo species, and then the constant terms and coefficients of each indicator were obtained.

#### (2) Multiple nonlinear regression (MNLR)

In order to analyze the possible nonlinear relationship between bamboo leaf indicators and their northernmost distribution zones, the SPSS 25.0 software (IBM SPSS Statistics for Windows, Version 25.0; Armonk, NY, USA) was employed to perform the multiple nonlinear regression analysis. The best univariate nonlinear regression model was established based on the dependent variables y (distribution zones) and the independent variables x_i_ (leaf indicators). All the curve models in SPSS software were tried to choose the best univariate regression model through the R^2^ value. The univariate nonlinear regression models were then artificially composed into a multivariate nonlinear regression model. The constant terms and indicator coefficients were subsequently calculated by SPSS nonlinear regression.

#### (3) Principal component analysis (PCA)

Principal component analysis (PCA) is a multivariate statistical technique used to reduce the number of variables in the data set into a smaller number of ‘dimensions’ (Vyas and Kumaranayake, 2006). PCA was performed on the selected indicators using SPSS25.0 to obtain eigenvalues, cumulative contribution rates, and principal component scores. Based on the PCA results, the bamboo distribution prediction model was established. First, the principal components with eigenvalues greater than 1 were selected. Each principal component was a linear weighted combination of the initial variables (Vyas and Kumaranayake, 2006). The calculation formula of the principal component were as follows:


PC 1 = a 11 x 1 + a 12 x 2 +⋯+ a 1n x n⋮PC m  = a m1 x 1 + a m2 x 2 +⋯+ a mnx n 

In the formula, x_1_ to x_n_ represented all variables, and a_mn_ represented the weight of the m^th^ principal component and the n^th^ variable. Each weight value was obtained based on the principal component score coefficient matrix.

##### a. Principal component analysis-multiple linear regression(PCA-MLR)

Multiple linear regression was carried out with the value of the selected principal component as independent variable and the bamboo distribution zones as dependent variable. The constant term and principal component were calculated by SPSS software.

##### b. Principal component analysis-multiple nonlinear regression(PCA-MNLR)

The optimal univariate nonlinear regression model of y (northernmost distribution zone) on principal component were established. All the curve models were tested in SPSS software in order to select the best univariate regression model according to the values of goodness of fit (R^2^). R² was defined as the ratio of the sum of squares of the regression and the total sum of squares (SST) (Petrini et al., 2009). The univariate nonlinear regression model was then artificially composed to a multivariate nonlinear regression model, and the constant term and factor coefficients were calculated by SPSS. The value of each main factor calculated by the principal component analysis was regressed by a univariate curve, and subsequently, the curve with the highest R^2^ was selected for the nonlinear regression.

### Re-establishment of mathematical model after exclusion of the indicators with weak correlation coefficient (|r|<0.3)

The above mathematical models were established based on all selected indicators (P<0.05). In statistics, the correlation coefficient less than 3 were considered as poor correlation (Gao and Yin, 2007). In order to increase the rigor, all the models were rebuilt by using those indicators with the correlation coefficient>3.0, i.e., MLR-E, MNLR-E, PCA-MLR-E and PCA-MNLR-E. Finally, the model with the highest goodness of R^2^ value was selected as the optimal model.

### Verification of prediction models

The northernmost distribution zones of the bamboo species involved in the model establishment were predicted via the above eight models once again, which were then compared with their actual distribution zones, and the R^2^ values were also calculated. Additionally, the potential distribution zones of a total of 6 bamboo species that did not participate in modeling were also predicted by using the eight models, and then were compared with their actual distribution zones so as to verify the prediction abilities of these models. Finally, the optimal prediction model was screened out according to the R^2^ values and prediction ability.

### Statistical analysis

All data for statistical analysis were determined three times and analyzed by using the Excel files (Microsoft Excel 2016) and SPSS 25.0. Data variance analysis was performed using the Analysis of Variance (ANOVA) method and the Least Significant Difference (LSD) test. The figures were made by using Origin 2019 software (OriginLab Corporation, Northampton, MA, USA), and TBtools VS 1.051 (https://www.tbtools.com) was used to visualize the heat map.

## Results

### The overwintering performance of leaves in different bamboo species

Different bamboo species showed different abilities in low-temperature tolerance in winter, which indicated different adaptabilities to the low temperature environment. During the overwintering period (from Nov. to the next Jan.) of different bamboo species in Kunming in 2019 and 2020, a total of 19 species were observed in the wild, so as to compare their differences in low-temperature tolerance (**Table 2**). Among which, no significant injuries of low temperature were observed in the leaves of *Phyllostachys edulis*, *Ph. nigra*, *Ph. bissetii*, *Bambusa multiplex*, *B. multiplex* f. *alphonso-karri*, *Fargesia fractiflexa*, *Bashania fargesii*, and *Qiongzhuea tumidinoda*, which implied high level of low-temperature tolerance. However, severe injuries were observed in the leaves of *Chimonocalamus pallens*, *Dendrocalamus brandisii, D. hamiltonii* and *D. farinosus*, which indicated their low level in low-temperature tolerance. The leaves of *Fargesia fungosa*, *F. yunnanensis*, *Pleioblastus amarus*, *Drepanostachyum scandeus*, *Bambusa rigida*, *B. textilis*, *Neosinocalamus affinis* suffered slight injuries, implying their middle level in low-temperature tolerance.

**Table 2:** Overwintering performance of different bamboo species.

Bamboo species	Overwintering performance of different bamboo species						Cold tolerance level
	Nov, 2019	Dec, 2019	Jan, 2020	Nov, 2020	Dec, 2020	Jan, 2021	
*Dendrocalamus brandisii*	++	++	+++	++	++	+++	Level 3
*Dendrocalamus farinosus*	+	++	+++	++	++	+++	Level 3
*Dendrocalamus hamiltonii*	++	++	+++	++	+++	+++	Level 3
*Neosinocalamus affinis*	+	+	++	+	++	+++	Level 2
*Bambusa multiplex*	+	+	+	+	+	++	Level 1
*Bambusa multiplex* f. *alphonso-karri*	+	+	++	+	++	++	Level 1
*Bambusa rigida*	+	++	++	+	++	+++	Level 2
*Bambusa textilis*	+	++	++	+	++	+++	Level 2
*Chimonocalamus pallens*	+	++	+++	+	+++	+++	Level 3
*Drepanostachyum scandeus*	+	++	++	+	++	++	Level 2
*Fargesia fractiflexa*	+	+	+	+	+	++	Level 2
*Fargesia fungosa*	+	+	++	+	+	++	Level 1
*Fargesia yunnanensis*	+	+	++	+	+	++	Level 2
*Bashania fargesii*	+	+	+	+	+	+	Level 1
*Qiongzhuea tumidinoda*	+	+	++	+	++	++	Level 1
*Pleioblastus amarus*	+	+	+	+	+	++	Level 2
*Phyllostachys bissetii*	+	+	++	+	++	++	Level 1
*Phyllostachys edulis*	+	+	++	+	+	++	Level 1
*Phyllostachys nigra*	+	+	+	+	+	+	Level 1

'+' indicated that no significant damage, '++' indicated that slightly cold damage, and '+++' indicated that severe cold damage.

### Comparison of photosynthesis, transpiration and water contents among different bamboo species

**Figure 3:**
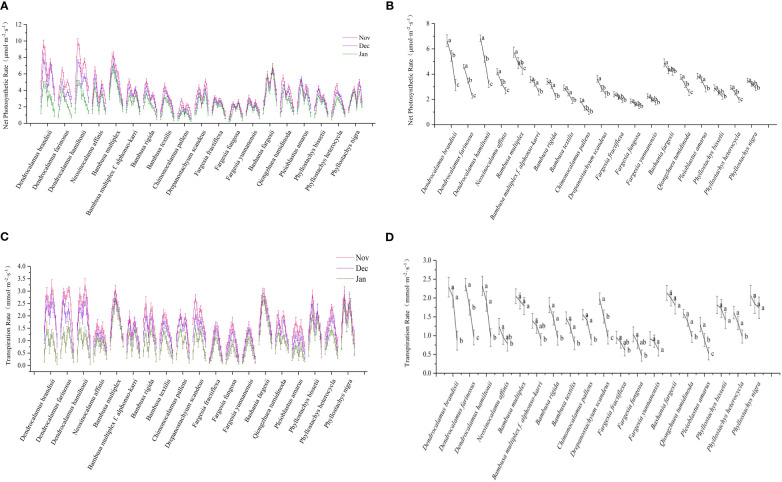
Net photosynthetic rate and transpiration rate of bamboo. **(A)** Diurnal variation of net photosynthetic rate of each bamboo species in daytime in different months. **(B)** Net photosynthetic rate of each bamboo species in different months. **(C)** Diurnal variation of transpiration rate of each bamboo species in daytime in different months. **(D)** Transpiration rate of each bamboo species in different months. Means with the same letters in each bamboo species was not significantly different (P < 0.05). Means with the different letters in each bamboo species was significantly different (P ≥ 0.05).

The photosynthesis of bamboo plants was affected by environmental changes, such as temperature and light intensity (Chen et al., 2018; Ji et al., 2018; Ng et al., 2020). Photosynthesis could be determined in all bamboo species in winter, and different photosynthesis rates were shown in different species (**Figures 3A, B**), implying no dormancy in bamboos in winter. Among all measured bamboo species, *Dendrocalamus brandisii*, *D. hamiltonii*, *Bambusa multiplex* showed higher net photosynthetic rates than *Fargesia yunnanensis*, *F. fractiflexa F. fungosa* and *Chimonocalamus delicatus* in winter. The net photosynthetic rates of different bamboo genera were shown in the following order: *Dendrocalamus* > *Bambusa* > *Phyllostachys* > *Fargesia*. Generally, the clustered bamboos showed higher net photosynthetic rates than the scattered bamboos, and in other words, the net photosynthetic rates were higher in the tropical bamboo species than in the temperate bamboo species in winter. This implied that the temperate bamboos decreased their photosynthesis in order to better adapt to the low-temperature winter.

Two types of diurnal variation curves were observed in the net photosynthesis of bamboos, i.e., single peak and double peak types (**Figure 3A**). The single peak curve showed that the net photosynthesis rates of leaves increased constantly with sunshine intensity and temperature with the maximum at noon and then decreased in the subsequent time (**Figure 3A**). The photosynthesis of *Bambusa multiplex*, *B. textilis*, and *Phyllostachys edulis* belonged to this type. The diurnal variation of net photosynthetic rates of most bamboo species showed the double peak curve. Additionally, the net photosynthetic rates of all bamboos decreased with months in winter (**Figure 3B**), implying that the net photosynthetic rates decreased constantly with temperature. Most bamboo species slightly decreased (P≥0.05) their photosynthesis from Nov to Jan except the tropical bamboos, such as *Dendrocalamus* species, which decreased significantly (P<0.05).

Similar to net photosynthetic rates, the transpiration rate curves also showed the single peak type and double peak type (**Figure 3C**). The transpiration rates of the single peak type gradually increased in the morning, reached the highest values at noon, and then decreased in the afternoon, such as *Bambusa multiplex*, *B. textilis*, and *Fargesia fractiflexa*. However, the bamboo species with double peak curve had midday depression in transpiration rate at noon, such as *Bambusa multiplex* f. *alphonso-karri*, *B. rigida*, *Chimonocalamus pallens*, and *Dendrocalamus brandisii*. Similar to net photosynthetic rates. The tropical bamboos also showed higher transpiration rates than the temperate bamboos in the winter seasons (**Figure 3D**). The transpiration rates of *Dendrocalamus* and *Bambusa* bamboos decreased significantly with months, while that of the *Fargesia* bamboos decreased slightly. In general, the net photosynthetic rates and transpiration rates decreased more significantly in the tropical bamboo species as compared to those temperate bamboos in winter. The tropical bamboo species showed higher net photosynthetic rates and transpiration rates but lower low-temperature tolerant ability as compared to those temperate ones in winter. This implied that the template bamboos decreased their photosynthesis and transpiration, so as to better adapt to the low temperature of winter seasons.

### Comparison of moisture contents in leaves

**Figure 4:**
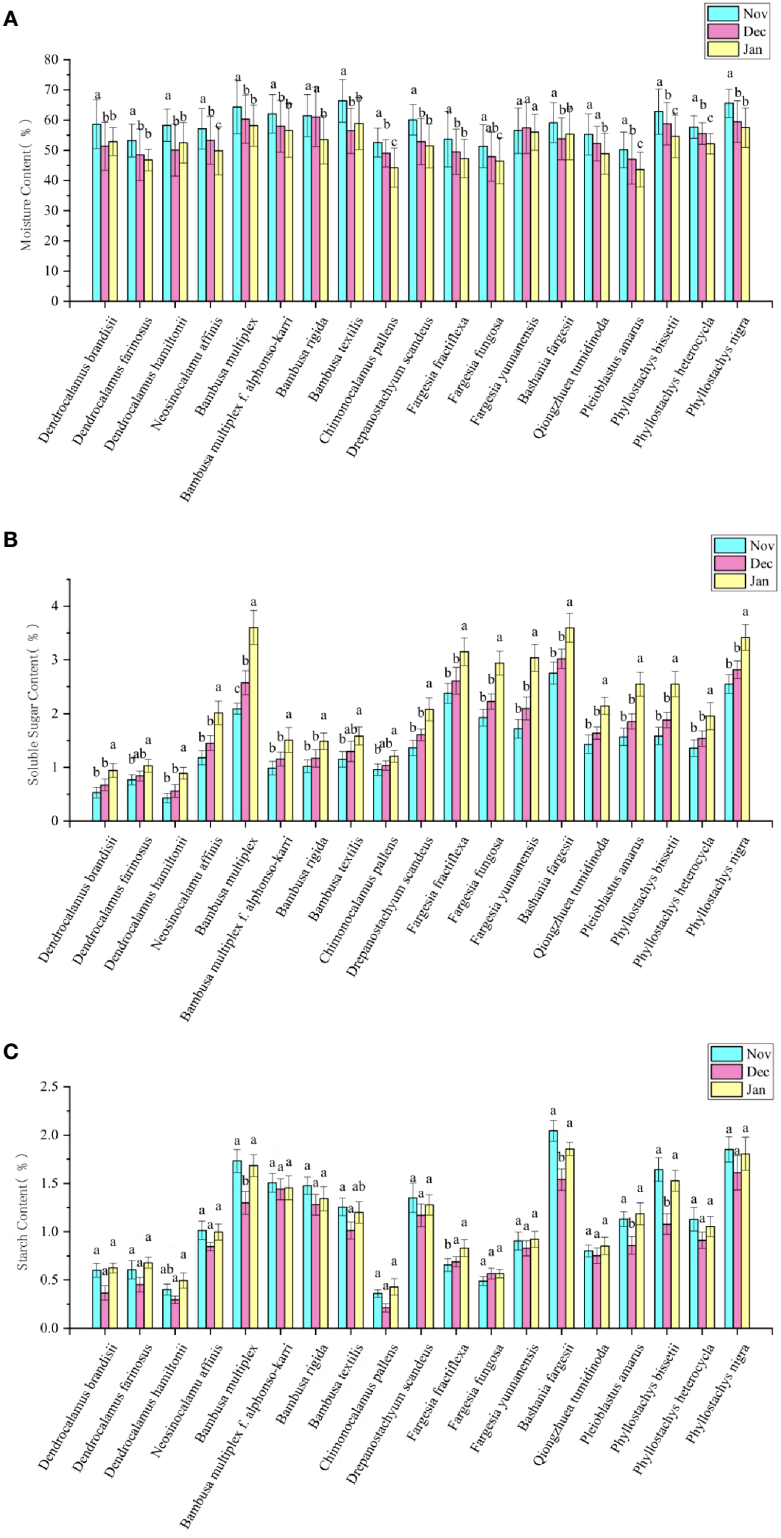
Changes of physiological indicators of bamboo leaves in different months. **(A)** Changes of moisture content in different months. **(B)** Changes of soluble sugar content in different months. **(C)** Changes of starch content in different months. Means with the same letters in each bamboo species was not significantly different (P < 0.05). Means with the different letters in each bamboo species was significantly different (P ≥ 0.05).

Different bamboo species showed different moisture contents in leaves in winter (**Figure 4A**). The moisture content was higher in the leaves of *Pleioblastus amarus*, *Fargesia fungosa* and *Chimonocalamus delicatus* than in those of *Bambusa textilis*, *B. multiplex* and *Phyllostachys nigra*. The moisture contents of leaves in different bamboo species showed a range from 50.16-66.41% in Nov, 47.11-61.05% in Dec, and 43.60-58.86% in Jan, which could also revealed that the moisture content showed a constantly decreasing trend with the month in most bamboo species. Additionally, we also noticed that the moisture content of *D. hamiltonii*, *D. brandisii*, *Bashania fargesii*, and *B. textilis* increased in the next Jan, while that of *F. yunnanensis* increased in Dec. and then decreased in the next Jan. In general, the leaf moisture contents of bamboo did not show apparent consistency with their natural distribution zones.

### Comparison and analysis of photoassimilates in leaves of different bamboo species

Soluble sugar contents were also different in the leaves of different bamboo species in winter (**Figure 4B**). The leaves of *Bambusa multiplex*, *Bashania fargesii* and *Phyllostachys nigra* showed higher soluble sugar contents, while those of *Dendrocalamus brandisii*, *D. farinosus* and *D. hamiltonii* showed lower contents as compared to those of other bamboo species. Generally, the temperate bamboo species showed higher soluble sugar content in leaves as compared to those tropical bamboo species. Contrary to the dynamics of net photosynthesis rates (**Figure 3B**), the soluble sugar content showed a constantly increasing trend in the leaves of most bamboo species with month, which slightly increased (P≥0.05) from Nov to Dec but significantly increased (P<0.05) from Dec to the next Jan. It could also be noticed that the soluble sugar contents increased more significantly in the temperate bamboo than in the tropical bamboo.

Starch was the main storage form of photoassimilates in plant cells (Malinova et al., 2018). Among all the bamboo species, the leaves of temperate *bamboos*, such as *Bashania fargesii* and *Phyllostachys nigra*, showed higher starch contents in all three months of winter, as compared to those of tropic bamboos, such as *Dendrocalamus brandisii*, *D. farinosus*, *D. hamiltonii* and *Chimonocalamus pallens* (**Figure 4C**). Almost the leaves of all the bamboo species showed lower starch contents in Dec than in Nov and the next Jan. Although the tropical bamboos showed higher photosynthesis, its soluble sugar and starch contents were lower than that temperate bamboo. Therefore, the photoassimilate contents in leaves might be closely related to their low-temperature tolerant abilities.

### Comparison of leaf morphological characteristics

**Figure 5:**
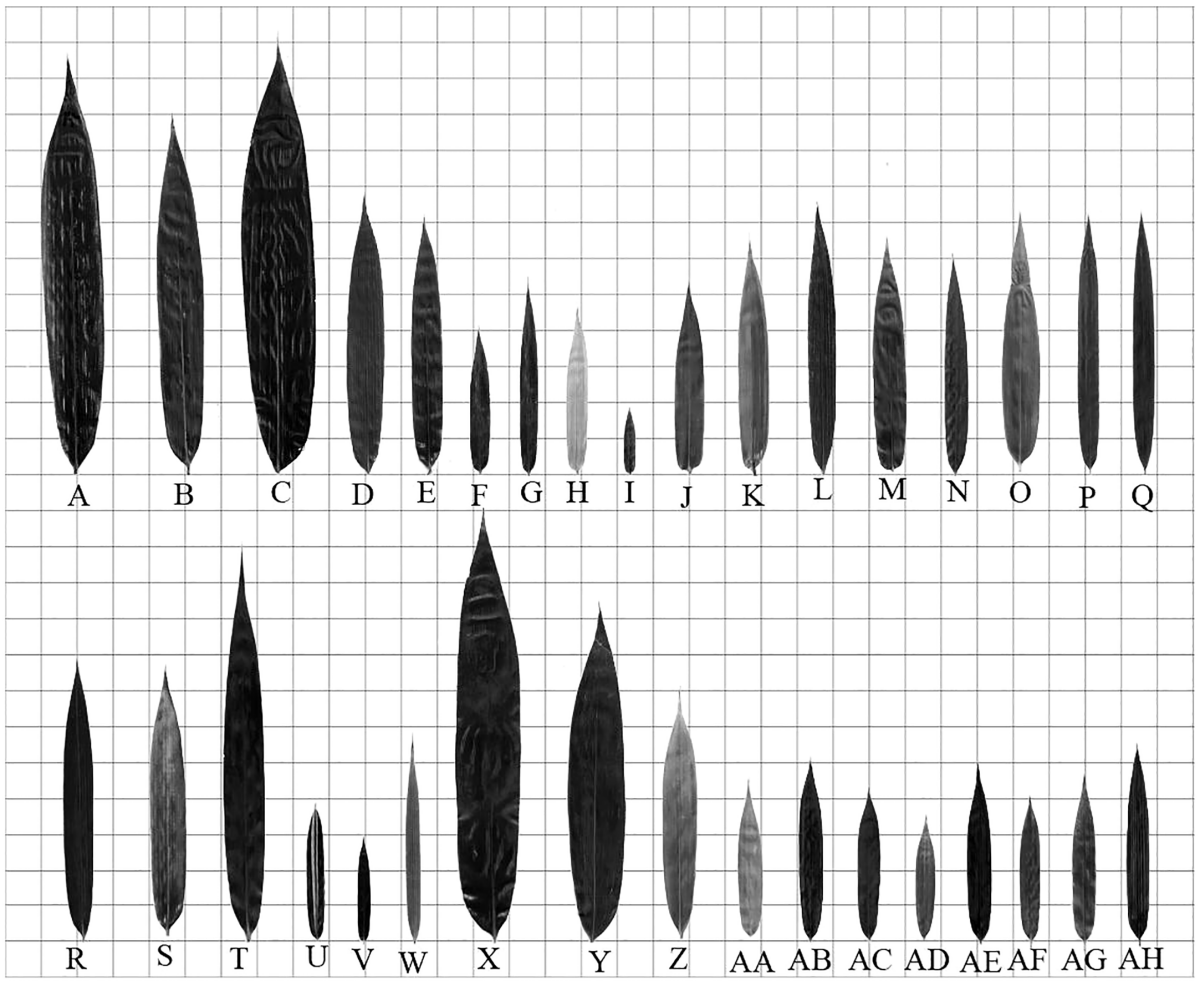
Leaf morphology and size of each bamboo species (each small grid side length = 25mm) **(A)** *Dendrocalamus brandisii*. **(B)** *Dendrocalamus farinosus*. **(C)** *Dendrocalamus hamiltonii*. **(D)** *Neosinocalamus affinis*. **(E)** *Neosinocalamus affinis* f. *viridiflavus*. **(F)** *Bambusa distegia*. **(G)** *Bambusa multiplex*. **(H)** *Bambusa multiplex* f. *alphonso-karri*. **(I)** *Bambusa multiplex* f. *fernleaf*. **(J)** *Bambusa rigida*. **(K)** *Bambusa textilis*. **(L)** *Bambusa tuldoides*. **(M)** *Bambusa ventricosa*. **(N)** *Chimonocalamus pallens*. **(O)** *Drepanostachyum scandeus*. **(P)** *Fargesia fractiflexa*. **(Q)** *Fargesia fungosa*. **(R)** *Fargesia yunnanensis*. **(S)** *Bashania fargesii*. **(T)** *Pseudosasa japonica*. **(U)** *Sasa fortunei*. **(V)** *Sasa pygmaea*. **(W)** *Qiongzhuea tumidinoda*. **(X)** *Indocalamus decorus*. **(Y)** *Indocalamus tessellatus*. **(Z)** *Pleioblastus amarus*. **(AA)** *Phyllostachys aurea*. **(AB)** *Phyllostachys aureosulcata f. spectabilis*. **(AC)** *Phyllostachys bissetii*. **(AD)** *Phyllostachys edulis*. **(AE)** *Phyllostachys mannii*. **(AF)** *Phyllostachys nigra*. **(AG)** *Phyllostachys nigra var. henonis*. **(AH)** *Phyllostachys vivax f. aureocaulis*.

The leaf blades of different bamboo species were different in morphology (**Figure 5**). According to the description of Yi and Shi (2008), the shape of bamboo leaves was classified into lanceolate, linear-lanceolate, ovate-lanceolate, oblong-lanceolate, long-elliptical and ovate. The leaves of *Dendrocalamus farinosus*, *Neosinocalamus affinis*, *N. affinis* f. *viridiflavus*, *Bambusa distegia*, *B. textilis*, *B. tuldoides*, *B. ventricosa*, *Drepanostachyum scandeus*, *Fargesia fractiflexa*, *F. fungosa*, *F. yunnanensis*, *Bashania fargesii*, *Pseudosasa japonica*, *Sasa fortunei*, *Qiongzhuea tumidinoda*, *Phyllostachys aurea*, *Ph. aureosulcata* f. *spectabilis*, *Ph. edulis*, *Ph. mannii* and *Ph. vivax* f. *aureocaulis* could be identified as the type of lanceolate (**Figures 5B, D–F, K–M, O–U, W, AA, AB, AD, AE, AH**). The leaves of *Bambusa multiplex*, *B. multiplex* f. *alphonso-karri*, *B. multiplex* f. *fernleaf*, *B. rigida*, *Chimonocalamus pallens*, *Sasa pygmaea*, *Ph. nigra*, *Ph. nigra* var. *Henonis* belonged to the linear-lanceolate type (**Figures 5G–J, N, V, AF, AG**). While those of *Dendrocalamus brandisii*, *D. hamiltonii* and *Indocalamus tessellatus* laeves were oblong-lanceolate types (**Figures 5A, C, Y**). Finally, the leaves of *Indocalamus decorus*, *Pleioblastus amarus*, and *Phyllostachys bissetii* could be identified as the type of long-elliptical (**Figures 5X, Z, AC**).

The leaf blade bases were also different in different bamboo species (**Figure 5**). The leaf blade bases of *Dendrocalamus brandisii, D. farinosus, D. hamiltonii, Bambusa rigida, B. tuldoides, Chimonocalamus pallens, Fargesia fractiflexa, F. fungosa, F. yunnanensis, Pseudosasa japonica, Qiongzhuea tumidinoda, Indocalamus tessellatus, Pleioblastus amarus, Phyllostachys bissetii, Ph.s edulis, Ph. mannii, Ph. nigra, Ph. vivax* f. *aureocaulis* belonged to cuneate type (**Figures 5A–D, J, L, N, P–R, T, W–Z, AC-AF, AH**). While those of *Neosinocalamus affinis, Neosinocalamus affinis* f. *viridiflavus, Bambusa distegia, B. multiplex, B. multiplex* f. *alphonso-karri, B. multiplex* f. *fernleaf, B. textilis, B. ventricosa, Drepanostachyum scandeus, Bashania fargesii, Sasa fortunei, S. pygmaea, Indocalamus decorus, Phyllostachys aurea, Ph. aureosulcata* f. *spectabilis, Ph. nigra* var. *Henonis* leaf blade bases were round type (**Figures 5E–I, K, M, O, S, U, V, X, AA, AB, AG**). Additionally, the leaf bases of most bamboo species were asymmetry between two sides. Unlike leaf bases, the leaf tips of most bamboo were of acuminate type (**Figure 5**).

It could be concluded that the leaf blades of tropical bamboos were usually in oblong-lanceolate and long-elliptical type, while the temperate bamboos were usually in lanceolate and linear-lanceolate type. Additionally, the bamboo species with the cuneate type of leaf bases were mainly distributed in tropical regions, while those bamboos with the round type of leaf bases were usually distributed in the temperate regions. This implied that the leaf blade shape was closely related to their distributions.

The leaf area, leaf vein density, stomatal density, leaf length and leaf width of 102 core collections from 34 bamboo species were measured, and the ratios of leaf length to leaf width and the ratio of leaf length and leaf width to leaf area were also calculated and presented in **Table 3**. There were significant difference (P<0.05) in leaf size among different species (**Table 3**; **Figure 5**). The bamboos of *Dendrocalamus* (**Table 3**; **Figures 5A–C**), *Pseudosasa* (**Table 3**; **Figure 5T**) and *Indocalamus* (**Table 3**; **Figures 5X, Y**) showed larger leaf areas than other bamboo species, among which *Dendrocalamus hamiltonii* showed the largest leaf blades with the length of 316.632 ± 19.2681 mm, the width of 49.2987 ± 6.3197 mm and the area of 11367.9778 ± 1728.7738 mm^2^ (**Table 3**; **Figure 5C**). The leaf blades of *Dendrocalamus*, *Neosinocalamus*, *Lingnania*, most of *Bambusa*, *Chimonocalamus*, *Drepanostachyum*, *Fargesia*, *Bashania*, *Qiongzhuea*, *Pleioblastus* and most of *Phyllostachys bamboos* were in the middle size with the length of 101.5147-198.8371 mm, the width of 11.8931-27.6091 mm and the area of 1033.6156-3698.9072 mm^2^. However, the leaf blades of *Bambusa multiplex* f. *fernleaf*, *Phyllostachys heterocycla*, *Sasa fortunei* and *Sasa pygmaea bamboos* were in a small size with a length of 47.0577-77.7952 mm, the width of 7.9864-12.2099 mm and the area of 310.3578-854.9518 mm^2^. The leaf blades of *Bambusa multiplex* f. *fernleaf* showed the smallest area, which was only 310.3578 mm^2^ (**Table 3**; **Figure 5I**).

**Table 3:** Morphological indicators of bamboo leaves.

Bamboo species	Leaf area (mm^2^)	Leaf vein density (cm^-1^)	Stomatal density (mm^-2^)	Leaf length (mm)	Leaf width (mm)	Leaf length/Leaf width
*Dendrocalamus brandisii*	10985.3525±1469.9934b	35.8607±1.8836r	341.3518±47.9843m	308.3940±23.8908f	50.1191±5.6537c	6.2538±0.9039r
*Dendrocalamus farinosus*	7450.1752±1472.2958c	42.9866±2.5940p	362.6540±67.6913m	269.0678±31.6063d	37.7017±3.7804f	7.1603±0.7204c
*Dendrocalamus hamiltonii*	11367.9778±1728.7738a	37.0529±3.9204qr	392.5317±78.3457jkl	316.6320±19.2681a	49.2987±6.3197a	6.5225±0.8915s
*Neosinocalamus affinis*	3698.9072±1660.3296f	44.6418±5.4978op	225.4497±71.2148q	196.1970±41.8872i	25.0203±5.6546h	7.8267±1.0156klm
*Neosinocalamus affinis* f. *viridiflavus*	2252.6480±446.8139k	54.3563±4.2540l	405.6255±50.6246hijk	173.0537±19.2610l	17.0043±2.1532mn	10.2733±1.2353q
*Bambusa distegia*	1033.6156±184.3167q	61.2119±2.4193hi	505.7545±62.866de	101.5147±13.2685bc	13.6063±1.4648c	7.5124±1.1850hi
*Bambusa multiplex*	1780.4084±644.4353lm	77.0009±4.6237b	557.3898±94.8986c	141.0962±30.4345j	15.4292±2.9100jk	9.1376±1.4278i
*Bambusa multiplex* f. *alphonso-karri*	1380.2887±437.6249n	90.4399±88.8674a	560.6764±139.4403c	116.3098±23.8141hi	15.0270±2.7099gh	7.8197±1.5220m
*Bambusa multiplex* f. *fernleaf*	310.3578±205.2770s	72.2071±8.0794c	516.4166±94.5051de	47.0577±17.3295i	7.9864±2.1824ij	5.7750±1.0212e
*Bambusa rigida*	2182.8065±202.8767k	62.7156±5.0982gh	635.5811±81.1892a	131.9743±7.8314j	22.9190±1.3358lm	5.7689±0.3664bc
*Bambusa textilis*	2491.4973±495.5809j	69.4774±5.0535d	615.5524±97.2334b	169.8458±10.0547f	19.3245±4.2097c	9.2297±2.1455a
*Bambusa tuldoides*	2759.6083±570.4703i	69.3326±6.7105d	385.3386±55.1608kl	182.5033±21.4779g	20.2753±2.9878ij	9.2752±1.4166b
*Bambusa ventricosa*	2878.7843±559.8495i	61.6486±6.3189hi	435.2972±50.2966g	165.9332±16.7433k	22.9238±3.3696ijk	7.3467±1.1549ij
*Chimonocalamus pallens*	1891.3563±141.2570l	47.2545±6.0018n	202.9135±31.8208r	154.7257±8.4021l	16.5273±1.2529no	9.4253±0.9901kl
*Drepanostachyum scandeus*	3494.0854±969.4964g	35.4122±1.8103r	321.5471±39.7591n	181.2488±20.6053l	27.6091±4.3332o	6.6157±0.4981jk
*Fargesia fractiflexa*	2175.0120±239.5068k	50.2260±6.1543m	298.3279±55.0662o	177.7318±8.0727jk	15.8332±1.5252h	11.3011±0.9102q
*Fargesia fungosa*	2114.2132±207.7249k	50.1691±5.9960m	262.2498±47.1145p	176.2912±12.265m	15.3685±1.388p	11.6045±1.6869p
*Fargesia yunnanensis*	3224.5721±537.2041h	67.7021±12.4047de	241.9723±54.1334q	198.8371±19.5431k	21.7534±2.5586kl	9.2563±1.2842gh
*Bashania fargesii*	3176.7733±630.3810h	50.1688±4.4079m	350.9776±70.9565m	188.6908±24.6992k	22.6616±3.0582ijk	8.4664±1.5468lm
*Pseudosasa japonica*	6384.7421±1415.8858d	38.6848±2.1890q	345.6452±55.1308m	278.3359±30.2657m	32.7869±5.3882p	8.6731±1.4915r
*Sasa fortunei*	854.9518±357.1474r	48.7316±5.7390mn	414.9822±91.4137hi	93.7474±24.6363i	11.5376±2.4836ef	8.2270±1.7904m
*Sasa pygmaea*	696.1832±191.6920r	50.9408±8.4590m	424.6199±79.4850gh	77.7952±12.1276k	11.9021±1.8173i	6.5999±0.9697op
*Qiongzhuea tumidinoda*	1205.1751±264.6319op	57.3509±4.8006jk	281.3191±62.3810op	144.0105±18.7493bc	11.8931±1.5543b	12.1763±1.3046jk
*Indocalamus decorus*	11034.0753±1882.9681b	39.3031±4.1269q	415.6284±45.9650ghi	297.0710±30.0014d	52.5277±8.9770def	5.8779±1.4239e
*Indocalamus tessellatus*	5972.3939±1395.3231e	38.8486±3.2404q	346.8858±47.4606m	229.5591±21.9093i	36.4516±6.5664i	6.4555±1.0193f
*Pleioblastus amarus*	2865.4564±751.9622i	46.9009±5.7968no	409.4605±64.4104hij	184.1897±38.9920a	21.6267±3.0588b	8.6684±2.6526n
*Phyllostachys aurea*	1358.8022±240.1113no	60.1997±3.6505hi	424.6199±33.9022gh	112.3031±11.3962e	17.0180±1.8644d	6.5676±0.7311fg
*Phyllostachys aureosulcata* f. *spectabilis*	1650.5925±561.2340m	64.7763±23.8130fg	522.7106±68.7271d	124.9588±25.4959f	17.6468±3.0679de	7.0335±0.9230jk
*Phyllostachys bissetii*	1299.0153±355.7895no	64.6681±5.6945fg	498.6234±68.4660ef	107.5915±16.6248b	16.5424±2.3801c	6.5517±1.0669d
*Phyllostachys edulis*	853.7851±391.2892r	64.6347±5.5294fg	387.3456±118.8575kl	93.6883±20.4315gh	12.2099±3.7628h	8.1966±2.2826d
*Phyllostachys mannii*	1637.2619±788.5555m	59.0747±4.1977ij	482.8268±71.9823f	124.8955±32.2034l	17.1440±5.0253o	7.3445±0.9832lm
*Phyllostachys nigra*	1068.1552±366.4300pq	62.7880±7.5288gh	382.3441±69.6607l	104.0998±16.5667c	13.7886±2.4852ef	7.6230±1.0144bc
*Phyllostachys nigra* var. *henonis*	1270.0791±364.3309no	66.6488±4.9079ef	360.2188±97.4310m	111.6150±15.8622i	15.0034±2.8709g	7.9228±0.9440no
*Phyllostachys vivax* f. *aureocaulis*	1737.4188±548.9689lm	55.8210±3.6447kl	397.9266±46.3475ijkl	139.5141±23.8676f	16.4159±2.9182c	8.5115±1.2716q

Means with the same letters in each column was not significantly different (P < 0.05). Means with the different letters in each bamboo species was significantly different (P ≥ 0.05).

The ratios of leaf length to width were directly related to their shapes. The ratio of leaf length to width of 34 bamboo species ranged from 5.7550 to 12.1637. The *Neosinocalamus affinis* f. *viridiflavus, Fargesia fractiflexa, F. fungosa, Qiongzhuea tumidinoda* showed high leaf length/leaf width ratios, which reached 10, while those of *Bambusa rigida, B. multiplex* f. *fernleaf and Indocalamus decorus* showed low ratios, which did not exceed 6. The leaf blades of *Qiongzhuea tumidinoda* showed the largest ratios, while those of *Bambusa rigida* showed the smallest ratio that was only 5.7689 ± 0.3664 (**Table 3**; **Figure 5W**). Generally, the tropical bamboo leaf blades showed higher leaf areas but lower leaf length/leaf width ratios than those of bamboos originated from the temperate regions. Leaf blades of bamboo species distributed in tropical regions tended to be larger and rounder, while those in temperate regions tended to be smaller and narrower.

In addition, the densities of veins and stomata were also important morphological characteristics for leaves. The leaf blades of *Bambusa multiplex*, *B. multiplex* f. *alphonso-karri*, *B. multiplex* f. *fernleaf*, *B. textilis B. tuldoides*, Phyllostachys nigra var. Henonis, *Ph. aureosulcata* f. *spectabilis*, *Ph. bissetii*, *Ph. edulis*, *Ph. Nigra* showed higher vein densities (**Table 3**), which from 62.7880 to 90.4399 cm^-1^. while those bamboo species with larger leaf areas showed lower vein densities, such as *Dendrocalamus brandisii*, *D. hamiltonii*, *D. scandeus*, *Indocalamus tessellatus*, *I. decorus*, and *Pseudosasa japonica*, which ranged from 35.412*2* to 38.6848 cm^-1^ (**Table 3**). Generally, the vein densities of leaf blades showed an opposite trend to their leaf areas, i.e., higher leaf area, and lower vein density. Meanwhile, the density of leaf veins was also related to their natural distributions, i.e., the temperate bamboo species showed higher vein densities as compared to those originated from the tropical regions.

There were also significant differences (P<0.05) in stomatal density among different bamboo species (**Table 3**), but from which no apparent trend could be drawn among different genera. Both the temperate *Fargesia* bamboos (ranging from 241.9723 to 298.3279 mm^-2^) and the tropical *Dendrocalamus* bamboos (ranging from 341.3518 to 392.5317 mm^-2^) had lower stomatal densities in leaves, while the most of *Bambusa* bamboos (*B. multiplex* f. *fernleaf*, *B. multiplex*, *B. multiplex* f. *alphonso-karri*, *B. textilis*, *B. rigida*) had higher stomata densities (ranging from 516.4166 to 635.5811 mm^-2^) as compared to other bamboos species (**Table 3**). The stomata densities of leaves could not reveal apparent relations to their distribution zones.

### Comparison of leaf anatomical characteristics among bamboo species

The leaf anatomical structures of different bamboo species were basically similar, including adaxial and abaxial epidermis, ground tissue and vascular bundle (**Figure S2**). The adaxial epidermises of most bamboo leaves were generally smooth with slightly wavy cell walls. The abaxial epidermises were rougher as compared to the adaxial epidermis, and more papillae were observed on their cuticles (**Figure S2**). Among all the bamboo species, the *Chimonocalamus pallens* leaves (**Figure S2N**; **Table 4**) showed the thickest adaxial epidermises, while the *Fargesia fractiflexa* leaves (**Figure S2P**; **Table 4**) showed the thinnest adaxial ones. As for the abaxial epidermis, the thickest ones were observed in *Pleioblastus amarus leaves*, while the thinnest ones were shown in *Fargesia fungosa leaves* (**Figures S2Z, Q**; **Table 4**). For the total epidermis thickness (the sum of adaxial and abaxial epidermises), the *F. fungosa* leaves showed the lowest values while the *Chimonocalamus pallens* leaves showed the highest values (**Figures S2N, Z**; **Table 4**).

**Table 4:** Anatomical indicators of bamboo leaves.

Bamboo species	Leaf thickness (mm)	Adaxial cuticle thickness (mm)	Abaxial cuticle thickness (mm)	Total cuticle thickness (mm)	Adaxial epidermis thickness (mm)	Abaxial epidermis thickness (mm)	Total epidermis thickness (mm)	Mesophyll thickness (mm)
*Dendrocalamus brandisii*	132.3866±37.0210m	4.2531±0.9450no	3.8265±7.0720c	8.0797±7.1728gh	9.6967±1.6921gh	6.7667±1.3607n	16.4635±2.4062l	101.5837±35.7222v
*Dendrocalamus farinosus*	189.0447±16.3600ijk	5.1358±0.7762lm	3.4458±0.7459s	8.5817±1.1312klm	12.9003±2.6813b	6.9390±1.4134c	19.8393±2.1173b	150.5483±16.2972pqr
*Dendrocalamus hamiltonii*	157.1430±37.6246jkl	3.8533±0.9964gh	2.8187±0.6616h	6.6720±1.4499de	11.3600±1.966op	6.2913±1.0417op	17.6513±2.4747u	123.5288±36.7040t
*Neosinocalamus affinis*	95.4381±9.2825b	3.8274±0.7960de	2.7480±0.5378e	6.5754±0.9607c	11.1762±2.1157gh	6.8206±1.3882e	17.9967±2.7227gh	65.5381±8.5145b
*Neosinocalamus affinis* f. *viridiflavus*	114.2059±16.0027m	3.7453±0.7148gh	2.8290±0.5599o	6.5743±0.9591gh	10.9717±1.4045hij	6.5276±1.0056j	17.4992±1.9008k	85.0630±13.8603u
*Bambusa distegia*	153.0081±13.0894a	5.6782±1.0973ef	3.8173±0.8913j	9.4955±1.5518d	18.4042±2.9496c	8.7008±1.6842g	27.1050±3.7616d	110.5481±11.1522a
*Bambusa multiplex*	116.2123±16.3097fg	3.4737±0.7281lm	2.6472±0.5949qr	6.1209±1.0018jkl	13.2935±2.0668gh	7.1688±1.2829hi	20.4623±2.5880j	84.8225±14.4489j
*Bambusa multiplex* f. *alphonso-karri*	123.8056±20.4834cd	3.2852±0.7075ij	2.6798±0.7241o	5.9650±1.1448hi	13.8337±3.0182no	7.2002±1.4728no	21.0339±3.8355st	90.0976±16.7924c
*Bambusa multiplex* f. *fernleaf*	114.8858±14.6391ijk	3.1518±0.9103lm	2.5105±0.7731f	5.6624±1.4570gh	13.8142±2.4151def	7.5650±1.6590m	21.3792±3.3715i	81.5573±11.6229pq
*Bambusa rigida*	178.5680±9.7293jkl	4.4638±0.5453o	3.4928±0.4929no	7.9567±0.7379lmno	15.7623±1.7042klm	8.4045±1.2874s	24.1668±1.8411st	137.5487±9.0462rs
*Bambusa textilis*	158.9229±34.3329jkl	4.108±0.7564hi	3.0091±0.6750k	7.1171±1.1615efg	15.2786±3.2688lmn	7.6950±1.4513pq	22.9736±4.1387rs	118.6320±30.0159pq
*Bambusa tuldoides*	141.8475±24.1353ijk	3.9502±0.7034kl	3.0609±0.6687n	7.0111±1.0424ij	15.7140±3.3810jk	8.1098±1.6520qr	23.8238±4.2394p	100.9572±18.4869n
*Bambusa ventricosa*	112.9672±13.4758hi	3.3730±0.8484lm	3.2637±6.3953u	6.6367±6.4878mno	13.4774±2.1373mno	7.4797±1.4498qr	20.9571±2.8271t	80.2959±12.4103m
*Chimonocalamus pallens*	128.7917±12.7178ab	4.0287±0.6710b	2.6748±0.6735a	6.7035±1.1369a	18.6390±1.3644a	9.0040±1.2999b	27.6430±1.9255a	93.1614±10.2043bc
*Drepanostachyum scandeus*	134.5431±8.5282fg	3.4671±0.8472d	2.7858±0.4914g	6.2529±1.0510c	10.4443±1.3360fgh	6.4360±1.3154kl	16.8803±1.7903j	103.7583±8.8204jk
*Fargesia fractiflexa*	107.8897±6.4531cd	2.8513±0.4867mn	2.4103±0.5702t	5.2617±0.6939lmno	7.8423±1.1404kl	6.2670±1.0541k	14.1093±1.5715o	85.3267±6.2622d
*Fargesia fungosa*	74.9358±5.4031jkl	2.6938±0.4964mn	2.2490±0.5185rs	4.9428±0.7513klmn	7.7960±1.3348efgh	5.2848±0.9225lm	13.0808±1.7912j	55.1153±5.1471st
*Fargesia yunnanensis*	116.6319±11.1753jkl	4.3831±1.0644lm	3.3485±0.8628p	7.7316±1.6589jklm	10.3841±1.6885kl	7.0332±1.5942qr	17.4173±2.5651q	86.2856±9.7194pqr
*Bashania fargesii*	137.6772±17.6904fg	5.2991±1.4808lm	3.2483±0.8559rs	8.5474±1.9889jklm	10.5413±1.8710mbo	7.6639±1.5781lm	18.2053±2.8654qr	103.2621±16.0369hi
*Pseudosasa japonica*	180.5492±20.0375c	5.5132±1.1314lm	3.2337±0.7198pq	8.7470±1.5481jk	15.4858±2.2693efg	9.0766±1.8447f	24.5624±3.1598fg	137.1826±16.8968bc
*Sasa fortunei*	156.0715±21.5335efg	4.4357±0.9629jk	3.1105±0.7709i	7.5463±1.4209gh	12.2868±2.2786de	9.2064±1.9554hi	21.4932±3.5955f	119.2591±18.9059kl
*Sasa pygmaea*	143.7017±25.0058fg	4.5740±0.9016gh	2.9808±0.7147h	7.5548±1.3120d	12.2713±1.9517d	8.2204±1.3631g	20.4918±2.5216e	108.4253±21.0756l
*Qiongzhuea tumidinoda*	98.4595±10.6556def	4.1949±1.1537fg	2.8485±0.6682jk	7.0434±1.5837d	10.3897±1.8094no	6.8180±1.0821n	17.2077±1.9687st	69.6235±11.3590ef
*Indocalamus decorus*	152.5302±17.0868l	5.4747±0.7641o	3.6068±0.6090pq	9.0814±1.0532no	12.0052±1.8268p	8.2210±1.1896t	20.2262±2.2538v	115.3468±15.7253op
*Indocalamus tessellatus*	152.2037±14.2131kl	5.3883±1.7085o	3.7183±1.1876t	9.1066±2.6780o	13.4179±3.1776ijk	8.2588±1.8151op	21.6767±4.3638o	113.3824±12.0626qrs
*Pleioblastus amarus*	156.7942±20.9952efg	5.4368±1.3791hi	3.2927±0.9617l	8.7295±1.9493gh	13.6447±2.6539ijk	9.6769±2.8268k	23.3215±4.6012mn	117.1139±20.2038fg
*Phyllostachys aurea*	135.1204±17.9809efg	4.5266±1.0737fg	3.1829±0.7671k	7.7095±1.5493def	10.5055±1.8338d	7.3689±1.2842gh	17.8744±2.4225e	101.6069±17.1268i
*Phyllostachys aureosulcata* f. *spectabilis*	132.346±18.232hij	4.5344±1.3328c	3.0180±0.7628d	7.5524±1.8379b	10.9431±1.9517de	7.3520±1.3473d	18.2951±2.7052e	98.5931±14.5269no
*Phyllostachys bissetii*	134.8525±13.3367cde	4.3101±1.0154gh	2.8859±0.757lm	7.1960±1.5062fg	10.9040±1.9968ijk	7.4662±1.3996ij	18.3702±2.7587lm	101.4268±11.4015e
*Phyllostachys edulis*	98.4745±10.1308h	3.7654±0.8497o	2.6462±0.6345pq	6.4117±1.2035no	9.6930±1.5901ghi	6.4284±1.2827r	16.1214±2.3534n	70.7377±9.2365l
*Phyllostachys mannii*	138.0393±12.4912defg	4.8180±1.0714hi	3.2155±0.8484m	8.0336±1.6005gh	10.1226±1.7255gh	7.1499±1.3597e	17.2724±2.5627hi	104.2167±11.6704gh
*Phyllostachys nigra*	116.2193±18.659def	3.7720±0.9647lm	2.6639±0.7271o	6.4359±1.4356jk	10.4908±1.9961c	7.1283±1.7708m	17.6191±3.2572e	86.4158±16.0468hi
*Phyllostachys nigra* var. *henonis*	145.4541±20.7099b	4.6929±1.4216a	3.1082±1.0283b	7.8011±2.1950a	11.7221±2.7022c	7.8656±1.7601a	19.5877±3.8307c	108.7081±15.3386b
*Phyllostachys vivax* f. *aureocaulis*	143.6813±19.0446g	4.6354±1.1016de	3.2151±0.7532jk	7.8504±1.5401d	11.7841±2.0540efgh	7.3719±1.4131d	19.1560±2.8931f	107.5820±16.8634jk

Means with the same letters in each column was not significantly different (P < 0.05). Means with the different letters in each bamboo species was significantly different (P ≥ 0.05).

Both the adaxial and abaxial leaf epidermises were covered by a layer of cuticle with different thickness in different bamboo species (**Figure S2**; **Table 4**). Meanwhile, the adaxial cuticle were thicker than the abaxial cuticle in most bamboo species, and among which the leaves of *Bambusa distegia* showed the thickest adaxial cuticle while the leaves of *Dendrocalamus brandisii* showed the thickest abaxial cuticle (**Table 4**). The total cuticle thickness (the sum of adaxial and abaxial cuticle thickness) was the thickest in *Bambusa distegia* and the thinnest in *Fargesia fungosa* (**Table 4**). In general, the leaf cuticle and epidermis thickness of sympodial bamboos were higher than those of monopodial bamboos.

Mesophyll cells were the most important part of the leaf. The bamboo species with larger leaf area usually had larger mesophyll thickness and leaf thickness than those with small leaf area such as *Dendrocalamus farinosus*, *D. hamiltonii*, *Pseudosasa japonica* (**Figures S2B, C, T**; **Table 4**). The temperate bamboo species also had thicker leaf thickness and mesophyll thickness, such as *Bashania fargesii*, *Phyllostachys nigra* var. *henonis* (**Figures S2S, AG**; **Table 4**). Additionally, the leaves of *Fargesia fungosa*, *Bambusa multiplex* f. *fernleaf* and *Ph. edulis* showed lower mesophyll thickness and leaf thickness as compared to other bamboo species (**Figure S2Q, I, AD**; **Table 4**). Therefore, the leaf thickness and mesophyll thickness of bamboo leaves showed no direct correlations with their low-temperature tolerance and distribution.

The ratios between anatomical indicators could also reflect the anatomical characteristics of bamboo leaves. The leaf length/leaf thickness ratios ranged from 0.4145 to 2.4899, among which the highest values were observed in the leaves of *Dendrocalamus hamiltonii*, *D. brandisii* and *Fargesia fungosa*, the middle values were observed in the leaves of *Bambusa textilis*, *Pleioblastus amarus*, *Chimonocalamus pallens*, and the lowest values were observed in the leaves of *Sasa pygmaea*, *S. fortunei, Phyllostachys edulis*, *Ph. aureosulcata* f. *spectabilis*, *Ph. nigra*, *Ph. mannii*, *Ph. aurea*, *Ph. bissetii*, *Ph. nigra* var. *henonis* (**Table S1**). For leaf width/leaf thickness, the range of values were from 0.0704 to 0.4087 with the smallest values in *Bambusa distegia*, *B. multiplex* f. *fernleaf*, *B. tuldoides*, *Phyllostachys nigra*, *Ph. nigra* var. *henonis*, *Ph. vivax* f. *aureocaulis*, *Sasa fortunei*, *S. pygmaea* leaves but the largest values in *Indocalamus decorus* and *D. brandisii leaves* (**Table S1**). Generally, the monopodial bamboo species showed lower ratios of leaf length/leaf thickness and leaf width/leaf thickness values as compared to those of sympodial bamboos (**Table S1**).

As compared to leaf thickness, the cuticle layers were relatively thinner and their total thickness did not exceed 10% of the leaf thickness *in all bamboo species* (**Table S1**). The largest ratios of adaxial cuticle/leaf thickness and total cuticle thickness/leaf thickness were shown in *Qiongzhuea tumidinoda leaves*, and the smallest ones were observed in *Dendrocalamus hamiltonii* (**Table S1**). The largest ratio of abaxial cuticle/leaf thickness was observed in *D. brandisii leaves*, and the smallest one was observed in *Pseudosasa japonica leaves* (**Table S1**). Therefore, the ratios of cuticle thickness to leaf thickness did not show apparent variation patterns in all bamboo species.

The largest ratios of adaxial epidermis thickness/leaf thickness and total epidermis thickness/leaf thickness were observed in *Chimonocalamus pallens* leaves, but the largest ratios of abaxial epidermis thickness/leaf thickness were observed in *Neosinocalamus affinis* leaves (**Table S1**). Besides, the leaves of *Dendrocalamus farinosus* showed the smallest ratios of adaxial epidermis thickness/leaf thickness, abaxial epidermis thickness/leaf thickness, total epidermis thickness/leaf thickness, but their ratios of mesophyll thickness/leaf thickness was the highest, and the lowest was observed in *Neosinocalamus affinis* leaves (**Table S1**). Generally, no apparent trend could be drawn in the ratios of epidermis thickness to leaf thickness among bamboo species.

For the ratio of leaf indicators to leaf area, the leaves of *Bambusa multiplex* f. *fernleaf* showed the highest value *among all the bamboo species* (**Table S2**), but it was lower for those bamboos with large leaves, such as *Indocalamus tessellatus*, *Dendrocalamus brandisii* and *D. hamiltonii* (**Table S2**). Generally, the ratios of cuticle thickness to leaf area were greater in the temperate bamboo species than in tropical bamboo.

### Variations in morphological and anatomical characteristics of bamboo leaves with seasons and regions

The variation degrees of all leaf morphological and anatomical indicators with seasons and regions were analyzed so as to screen out the stable inherent genetic indicators that could determine the northernmost distributions of different bamboo species, which could also be applied for the establishment of mathematical model for bamboo distribution prediction.

It was observed that the leaf shapes and anatomical structures of most bamboo species changed with seasons (**Figures S3–S6**). The leaf area, length, and width were lower in winter than in summer (**Figures S3A, D, E**), but the length/width of leaves showed no apparent trend (**Figure S3C**). Similarly, both stomata density and leaf vein density increased in summer, which might be mainly due to the newly sprouted leaves in summer (**Figures S3B, F**). Meanwhile, the leaf thickness and the thickness of adaxial cuticle, total cuticle, adaxial and abaxial epidermis, total epidermis, and mesophyll also increased in summer except the thickness of abaxial cuticle (**Figures S4A–H**). There were no regular trends in the ratio of leaf width/leaf thickness and leaf length/leaf thickness between seasons (**Figures S5A, B**). However, we founded a drop in the mesophyll thickness/leaf thickness but an increase in the ratio of other anatomical indicators to leaf thickness in summer as compared to those in winter (**Figures S5C–I**). Additionally, the ratios of leaf length and width to leaf area declined, but the ratios of all anatomical indicators to leaf area were higher in summer than in winter (**Figures S6A–J**).

**Figure 6:**
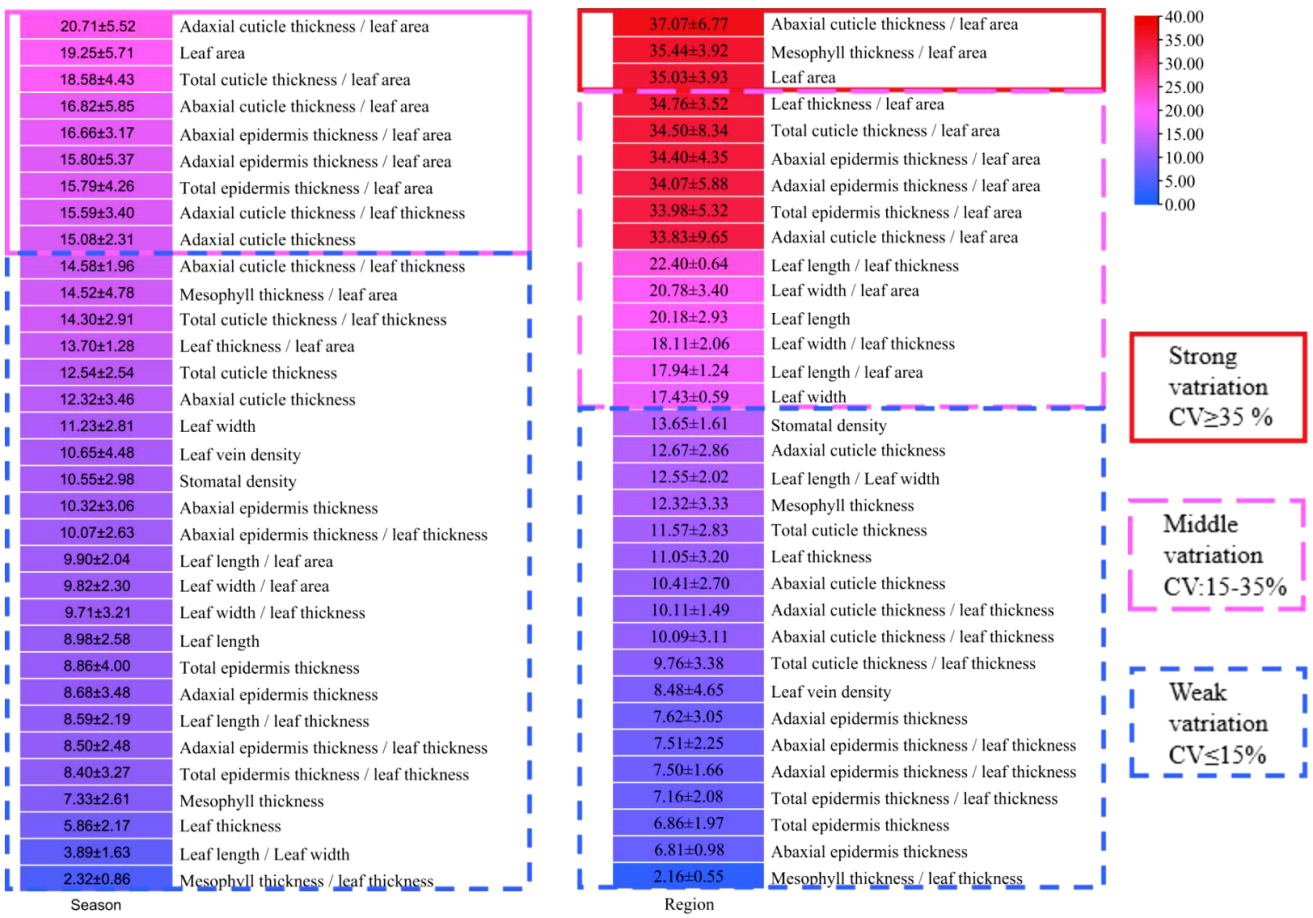
The variation of each indicator in different seasons and different regions.

The coefficient of variation (CV) of all indicators were analyzed in all leaf samples according to the methods of Canchola et al. (2017), which could reflect the variation degree and stability of leaves. The variation degree of all indicators could be classified into three levels, i.e., weak variation (0-15%), middle variation (15-35%) and strong variation (35-100%) according to the CV value (Guan et al., 2012). As shown in **Figure 6**, there were no strong variation in leaf indicators between summer and winter. The adaxial cuticle thickness, leaf area, adaxial cuticle thickness/leaf area, total cuticle thickness/leaf area, abaxial cuticle thickness/leaf area, abaxial epidermis thickness/leaf area, adaxial epidermis thickness/leaf area, total epidermis thickness/leaf area and adaxial cuticle thickness/leaf thickness were all in meddle variation level, ranging from 15 to 35%. However other indicators showed a weak variation level, which ranged from 0 to 15% (**Figure 6**). Therefore, seasons did not cause trong variations in the leaf morphological and anatomical characteristics of all bamboo species.

The morphological and anatomical characteristics of bamboo leaves changed with regions. Bamboo leaves grew larger in southern regions than in northern regions due to their increase of leaf area, leaf length and leaf width (**Figures S7A, D, E**). However, the bamboos in the northern regions usually showed higher vein densities and lower stomata densities (**Figures S7B, C**) and greater leaf length/width ratios (**Figure S7F**) as compared to those in the southern regions. The bamboo leaves became thicker in the southern regions, and the thickness of adaxial cuticle, abaxial cuticle, total cuticle, adaxial epidermis, abaxial epidermis, total epidermis and mesophyll also showed higher values in the southern regions than in the northern regions (**Figures S8A–H**). Bamboos in the northern regions usually had lower values in the ratios of leaf length and width to leaf thickness than the bamboos in the southern regions (**Figures S9A, B**). In addition, the ratio of mesophyll thickness/leaf thickness also showed lower values in northern regions as compared to those in the southern regions, which implied higher proportions of cuticle and epidermis in the northern regions. The ratios of adaxial cuticle, abaxial cuticle, total cuticle, adaxial epidermis, abaxial epidermis, and total epidermis thickness to leaf thickness tended to be higher in lower-temperature regions in deed (**Figures S9B–I**). This implied that the higher thickness of cuticle and epidermis of bamboo leaves was closely related to their low temperature resistance and distribution. Similarly, the ratios of leaf length, leaf width, leaf thickness, adaxial cuticle thickness, and mesophyll thickness to leaf area tended to be larger in the low-temperature regions. The ratios of abaxial cuticle, total cuticle thickness, adaxial epidermis and abaxial epidermis thickness to leaf area showed no regular trends with regions (**Figures S10A–J**).

For analysis of the variation degrees of bamboo leaves with regions, the leaf indicators of three *Phyllostachys* species were compared among Kunming City, Changsha City, Nanjing City, Xuancheng City and Beijing City (**Figure 6**). The results showed that the CV of leaf area, abaxial cuticle/leaf area and mesophyll thickness/leaf area were greater than 35%, which were all in the strong variation level (**Figure 6**), while the CV of leaf thickness/leaf area, total cuticle thickness/leaf area, abaxial epidermis thickness/leaf area, adaxial epidermis thickness/leaf area, total epidermis thickness/leaf area adaxial cuticle thickness/leaf area, leaf length, leaf length/leaf thickness, leaf length/leaf area, leaf width, leaf width/leaf area and leaf width/leaf thickness ranged from 15% to 25%, which were all in the middle variation level (**Figure 6**). The indicators, including stomatal density, adaxial cuticle thickness, abaxial cuticle thickness, total cuticle thickness, leaf length/leaf width, mesophyll thickness, leaf thickness, adaxial cuticle thickness/leaf thickness, abaxial cuticle thickness/leaf thickness, total cuticle thickness/leaf thickness, leaf vein density, adaxial epidermis thickness, abaxial epidermis thickness/leaf thickness, adaxial epidermis thickness/leaf thickness, total epidermis thickness/leaf thickness, total epidermis thickness, abaxial epidermis thickness and mesophyll thickness/leaf thickness, were all in weak variation level due to their low CV of 0-15%. Generally, the indicators related to leaf area were more variable because the leaf area was more easily influenced by the environmental conditions compared with other indicators, while the ratio of mesophyll thickness/leaf thickness was the most stable due to its lowest variability (**Figure 6**).

### Correlations between morphological and anatomical indicators and distribution zones

The above results showed that regions caused strong variations in the morphological and anatomical characteristics of leaves. Therefore, we selected the leaves of 29 bamboo species as samples in the same city of Kunming in winter to analyze the correlations between leaf indicators and their northernmost distribution zones, and to further screen out the key indicators related to the bamboo distribution for the subsequent establishment of mathematical model for bamboo distribution zone prediction.

**Figure 7:**
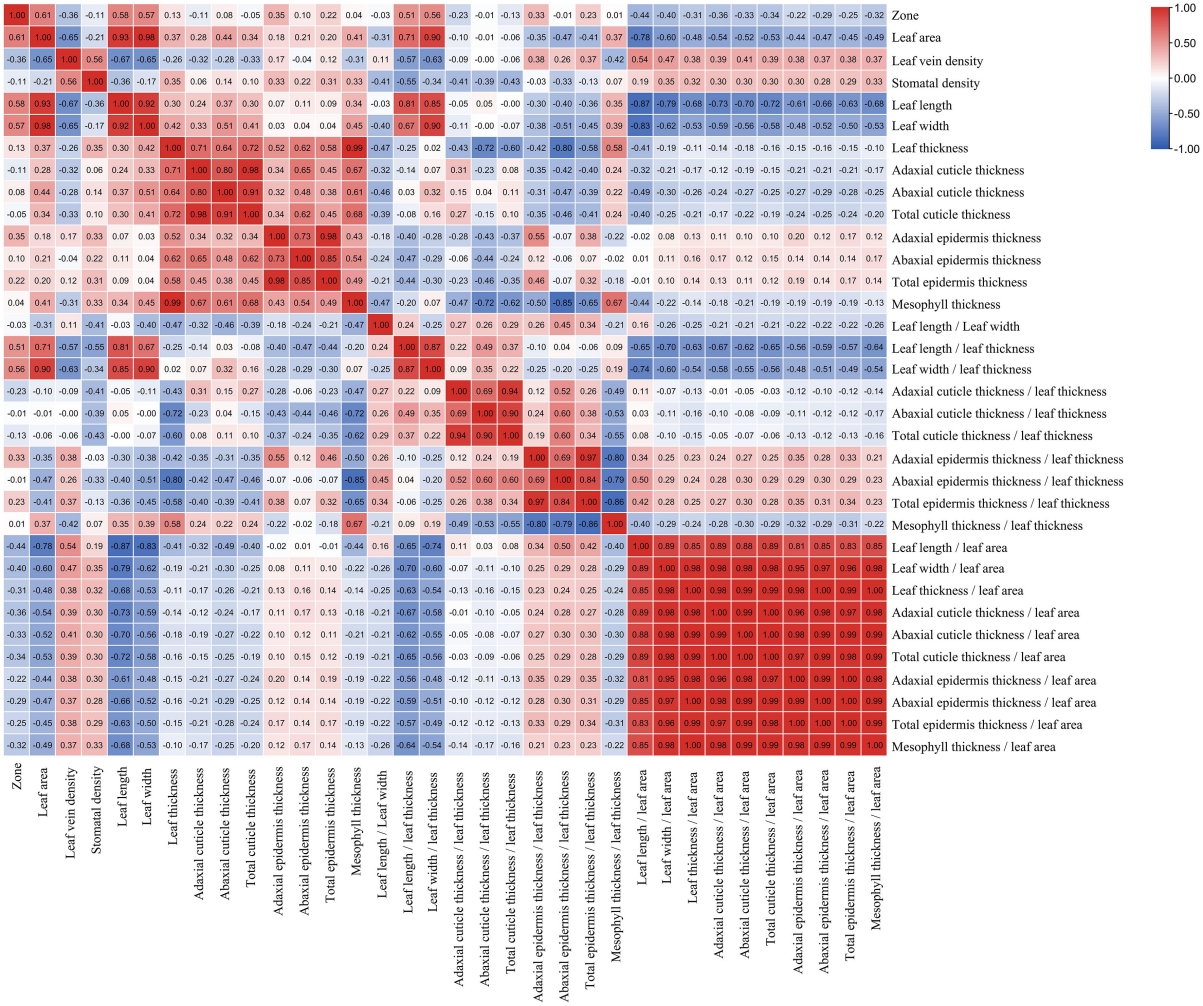
The heatmap of correlations among the each indicator and distribution zone (red: positively correlated; blue: negatively correlated).

According to the correlation analysis, a total of 14 indicators were shown to be correlated with the distribution zones of bamboos (**Figure 7**), and among which the leaf area, leaf length, leaf width, adaxial epidermis thickness, epidermis thickness, leaf length/leaf thickness, leaf width/leaf thickness, adaxial epidermis thickness/leaf thickness, epidermis thickness/leaf thickness were significantly and positively correlated with the bamboo distribution zone (P<0.05) (**Figure 7**). However, the leaf length/leaf area, leaf width/leaf area, leaf vein density, adaxial cuticle thickness/leaf area, and total cuticle thickness/leaf area were shown to be significantly and negatively correlated with the bamboo distribution zones (P<0.05) (**Figure 7**). Additionally, no significant and positive correlations (P≥0.05) were shown between the distribution zones and other 19 indicators, including leaf thickness, abaxial epidermis thickness, abaxial cuticle thickness, mesophyll thickness, abaxial cuticle thickness/leaf thickness, mesophyll thickness/leaf thickness, abaxial epidermis thickness/leaf thickness, length/width, total cuticle thickness, stomatal density, adaxial cuticle thickness, cuticle thickness/leaf thickness, adaxial cuticle thickness/leaf area, adaxial cuticle thickness/leaf thickness, epidermis thickness/leaf area, abaxial cuticle thickness/leaf area, leaf thickness/leaf area, mesophyll thickness/leaf area, abaxial cuticle thickness/leaf area (**Figure 7**).

### Establishment of mathematical model for bamboo distribution prediction

The indicators with strong variation were not suitable for the establishment of mathematical model for bamboo distribution prediction. This was mainly because these indicators with strong variations were easily influenced by the environmental conditions, and decreased the prediction accuracy of mathematical model. Hence, stable indicators should be selected to reduce the environmental influence on the accuracy of the prediction model and to ensure its application in different regions and seasons. The physiological indicators of leaves were easily affected by the external environment conditions, such as net photosynthetic rate, transpiration rate, moisture content, soluble sugar and starch content. Seasons did not cause strong variations (CV≥35%) in all leaf morphological and anatomical indicators. However, regions could cause strong variations in the indicators of leaf area, abaxial cuticle/leaf area and mesophyll thickness/leaf area, which should be excluded during model establishment.

During the establishment of mathematical models, the northernmost distribution zones of bamboos were assumed as dependent variable (y), constant term as x_0_, and the leaf indicator as independent variables (x), i.e., leaf vein density = x_1_, leaf length = x_2_, leaf width = x_3_, adaxial epidermis thickness = x_4_, total epidermis thickness = x_5_, leaf length/leaf thickness = x_6_, leaf width/leaf thickness = x_7_, adaxial epidermis thickness/leaf thickness = x_8_, total epidermis thickness/leaf thickness = x_9_, leaf length/leaf area = x_10_, leaf width/leaf area = x_11_, adaxial cuticle/leaf area = x_12_, total cuticle thickness/leaf area = x_13_.

#### (a) Multiple linear regression (MLR)

The multiple linear regression analysis was carried out by using SPSS software to calculate the constant term, indicator coefficient, and then the following equation was gotten:


$$\text{y}=1.681-0.008{\text{x}}_{1}-0.010{\text{x}}_{2}+0.149{\text{x}}_{3}-0.331{\text{x}}_{4}+0.289{\text{x}}_{5}+2.638{\text{x}}_{6}-9.189{\text{x}}_{7}+179.648{\text{x}}_{8}-121.779{\text{x}}_{9}+44.883{\text{x}}_{10}+200.331{\text{x}}_{11}+1004.379{\text{x}}_{12}-922.690{\text{x}}_{13}$$

#### (b) Multiple nonlinear regression (MNLR)

The results of the univariate nonlinear regression model of single indicator were shown in **Table S3**. According to the calculation results, the single regression model with the highest R^2^ was selected to synthesize the following multi-factor nonlinear regression model:


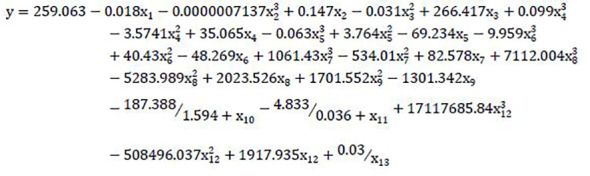


#### (C) Principal component analysis (PCA)

The principal component analysis was applied by using SPSS software, and the principal components with eigenvalue greater than 1 were selected. The results showed that the cumulative contribution rate of the four principal components was 94.964%, and only 5.036% of the information was lost (**Table S4**). The principal component 1 (PC_1_) was assumed as y_1_, principal component 2 (PC_2_) a y_2_, principal component 3 (PC_3_) as y_3_, and principal component 4 (PC_4_) as y_4_. According to the component score matrix and the value of each indicator (**Table S5**), the four principal components were expressed as:


$${\text{y}}_{1}=0.094{\text{x}}_{1}-0.129{\text{x}}_{2}-0.12{\text{x}}_{3}-0.339{\text{x}}_{4}+0.039{\text{x}}_{5}-0.115{\text{x}}_{6}-0.119{\text{x}}_{7}+0.064{\text{x}}_{8}+0.067{\text{x}}_{9}+0.128{\text{x}}_{10}+0.123{\text{x}}_{11}+0.12{\text{x}}_{12}+0.119{\text{x}}_{13}$$

$${\text{y}}_{2}=0.023{\text{x}}_{1}+0.07{\text{x}}_{2}+0.046{\text{x}}_{3}+0.344{\text{x}}_{4}+0.325{\text{x}}_{5}+0.002{\text{x}}_{6}+0.003{\text{x}}_{7}+0.277{\text{x}}_{8}+0.227{\text{x}}_{9}-0.1{\text{x}}_{10}-0.097{\text{x}}_{11}-0.089{\text{x}}_{12}-0.088{\text{x}}_{13}$$

$${\text{y}}_{3}=0.086{\text{x}}_{1}-0.016{\text{x}}_{2}-0.121{\text{x}}_{3}-0.264{\text{x}}_{4}-0.323{\text{x}}_{5}+0.348{\text{x}}_{6}+0.149{\text{x}}_{7}+0.363{\text{x}}_{8}+0.446{\text{x}}_{9}+0.116{\text{x}}_{10}-0.027{\text{x}}_{11}-0.044{\text{x}}_{12}-0.029{\text{x}}_{13}$$

$${\text{y}}_{4}=-0.403{\text{x}}_{1}+0.186{\text{x}}_{2}+0.328{\text{x}}_{3}+0.078{\text{x}}_{4}+0.09{\text{x}}_{5}+0.146{\text{x}}_{6}+0.336{\text{x}}_{7}+0067{\text{x}}_{8}+0.06{\text{x}}_{9}+0.097{\text{x}}_{10}+0.314{\text{x}}_{11}+0.38{\text{x}}_{12}+0.391{\text{x}}_{13}$$

According to the preliminary analysis results of PCA, further regression analysis was performed. The results based on PCA were modeled by multiple factor linear regression and multiple factor nonlinear regression.

##### (1) PCA-MLR analysis

y was set as the dependent variable, y_1_, y_2_, y_3_, y_4_ as independent variables, and the multiple linear regression was carried out. Finally, the equation was obtained as follows:


$$\text{y}=5.335-0.07{\text{y}}_{1}+0.098{\text{y}}_{2}-0.062{\text{y}}_{3}-0.044{\text{y}}_{4}$$

##### (2) PCA-MNLR analysis

y was set as the dependent variable while y_i_ was set as the independent variable for single factor curve regression analysis, and the maximum R^2^ value regression curve was selected (**Table S6**). The univariate regression curve was combined into a multivariate curve by using SPSS software, in order to calculate the constant term and coefficient, and then the equation was obtained:


$$\text{y}=-6.309-0.019{\text{y}}_{1}^{2}-0.218{\text{y}}_{1}-0.039{\text{y}}_{2}^{2}+1.253{\text{y}}_{2}-0.005{\text{y}}_{3}^{3}-0.104{\text{y}}_{3}^{2}-0.606{\text{y}}_{3}-0.00006113{\text{y}}_{4}^{3}+0.005{\text{y}}_{4}^{3}-0.33{\text{y}}_{4}$$

### Re-establishment of mathematical model after exclusion of those indicators with weak correlation coefficient

Correlation coefficient (r) represented the correlation between two variables, which was divided into three levels: high correlation (|r|≥0.8), moderate correlation (0.5≤|r|<0.8), weak correlation (0.3≤|r|<0.5), extremely weak correlation (|r|<0.3) (Gao and Yin, 2007). To increase the accuracy of the models, the above four mathematical models were re-established by excluding the indicators with extremely weak correlation coefficients with the northernmost distribution zones of bamboo. The re-established models were listed as follows:

#### (a) Multiple linear regression (MNR-E)

According to multiple linear regression analysis, the equation was re-established with exclusion of the indicators with extremely weak correlation coefficients.


$${\text{y}}_{1}=-1.549-0.002{\text{x}}_{1}-0.013{\text{x}}_{2}+0.206{\text{x}}_{3}+0.228{\text{x}}_{4}+2.942{\text{x}}_{6}-16.321{\text{x}}_{7}+5.208{\text{x}}_{8}+38.243{\text{x}}_{10}+222.019{\text{x}}_{11}+472.326{\text{x}}_{12}-620.374{\text{x}}_{13}$$

#### (b) Multiple nonlinear regression (MNLR-E)

The multivariate nonlinear regression model was carried out by using SPSS, and the equation was obtained with exclusion of the indicators with extremely weak correlation coefficients.


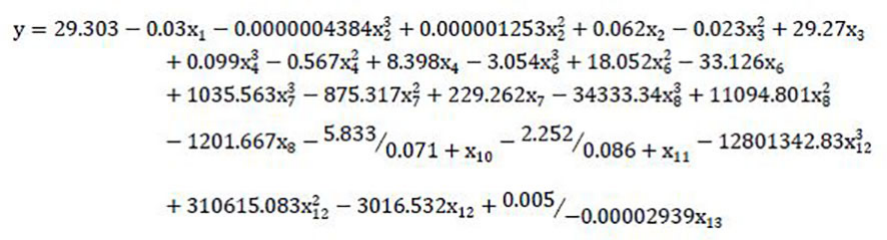


#### (c) PCA analysis

The PCA software was carried out by using SPSS software, and the principal components with eigenvalues greater than 1 were selected (**Table S7**). According to the results of PCA, the first three principal components were selected with the cumulative contribution rate of 87.083% (**Table S7**). The principal component 1 (PC_1_) was assumed as y_1_, the principal component 2 (PC_2_) as y_2_, the principal component 3 (PC_3_) as y_3_. Based on the component score matrix and the value of each indicator (**Table S8**), the equations were obtained and the three PCs could be expressed as follows:


$${\text{y}}_{1}=0.097{\text{x}}_{1}-0.135{\text{x}}_{2}-0.125{\text{x}}_{3}+0.03{\text{x}}_{4}-0.121{\text{x}}_{6}-0.124{\text{x}}_{7}+0.054{\text{x}}_{8}+0.135{\text{x}}_{10}+0.13{\text{x}}_{11}+0.127{\text{x}}_{12}+0.126{\text{x}}_{13}$$

$${\text{y}}_{2}=0.187{\text{x}}_{1}+0.02{\text{x}}_{2}-0.042{\text{x}}_{3}+0.537{\text{x}}_{4}-0.077{\text{x}}_{6}-0.115{\text{x}}_{7}+0.445{\text{x}}_{8}-0.131{\text{x}}_{10}-0.17{\text{x}}_{11}-0.178{\text{x}}_{12}-0.177{\text{x}}_{13}$$

$${\text{y}}_{3}=-0.341{\text{x}}_{1}+0.209{\text{x}}_{2}+0.34{\text{x}}_{3}+0.326{\text{x}}_{4}+0.124{\text{x}}_{6}+0.317{\text{x}}_{7}+0.282{\text{x}}_{8}+0.055{\text{x}}_{10}+0.28{\text{x}}_{11}+0.347{\text{x}}_{12}+0.361{\text{x}}_{13}$$

Similarly, based on the results of PCA, the multiple linear regression and multiple nonlinear regression were used to re-establish the following PCA-MLR analysis (PCA-MLR-E) and MNLR-PCA (PCA-MNLR-E) analysis with the exclusion of the indicators with extremely weak correlation coefficients.

#### (1) PCA-MLR-E analysis

According to the multiple linear regression results of PCA factors and distribution zones by using SPSS software, the equation was re-obtained as follows:


$$\text{y}=5.814+0.156{\text{y}}_{1}+0.131{\text{y}}_{2}+0.119{\text{y}}_{3}$$

#### (2) PCA-MNLR-E analysis

According to the univariate nonlinear regression results of PCA factors and distribution zones (**Table S9**), a multivariate nonlinear regression model was synthesized:


$$\text{y}=19.856+0.008{\text{y}}_{1}^{2}+0.499{\text{y}}_{1}+0.032{\text{y}}_{2}^{2}-1.155{\text{y}}_{2}+0.00001637{\text{y}}_{3}^{3}-0.004{\text{y}}_{3}^{2}+0.287{\text{y}}_{3}$$

### Verification of model prediction ability

**Figure 8:**
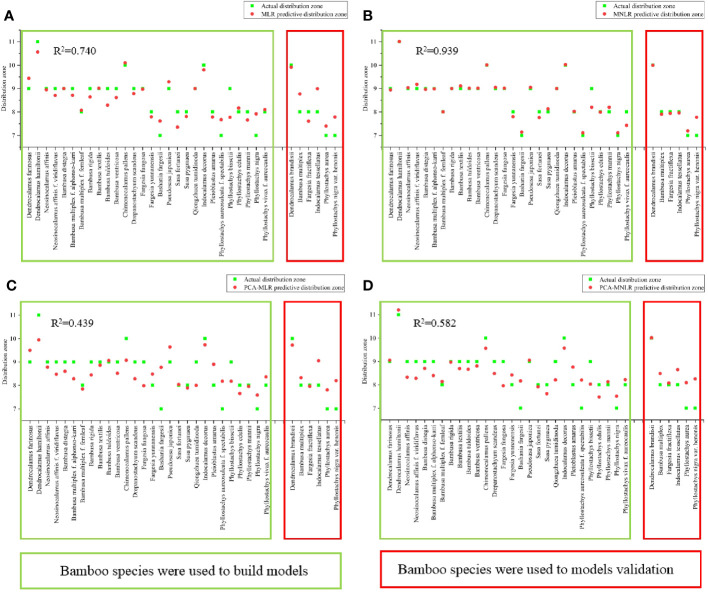
The predicted values of each model were compared with the actual value and the test of the model. **(A)** The predicted and actual values of MLR model. **(B)** The predicted and actual values of MNLR model. **(C)** The predicted and actual values of PCA-MLR model. **(D)** The predicted value and actual value of PCA-MNLR model.

**Figure 9:**
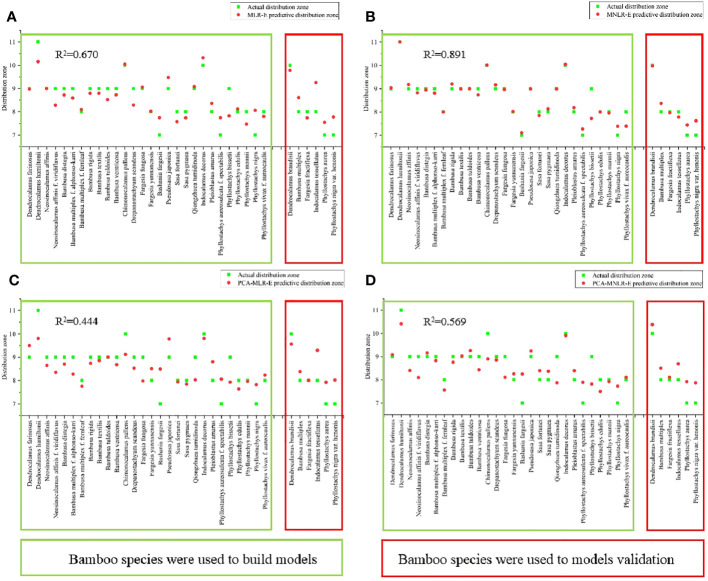
The predicted values of the re-established models were compared with the actual values and tested by the model. **(A)** The predicted and actual values of MLR-E model. **(B)** The predicted and actual values of MNLR-E model. **(C)** The predicted and actual values of PCA-MLR-E model. **(D)** The predicted value and actual value of PCA-MNLR-E model.

In order to analyze the accuracy of different models, the R^2^ of each model was calculated (**Figures 8** and **9**), and the predictive distribution zones and the actual distribution zones of all bamboo species were also compared.

For the MLR model (**Figure 8A**), the predictive distribution zones were basically close to the actual distribution zones in most bamboo species that were employed in the model establishment, and the R^2^ value of which reached 0.740. It was also noticed that the predictive distribution zones were lower than the actual distribution zones in almost all bamboo species. Additionally, the predictive distribution zones were quite different from the actual distribution zones in six bamboo species that were not employed during model establishment, which revealed the low predictive ability of MLR model. For MNLR model (**Figure 8B**), its R^2^ reached up to 0.939, and the actual distribution zones of the bamboo species were very similar to their predictive distribution zones, which showed better predictive ability. In the bamboo species that were not involved in the modeling, their predictive distribution zones were almost the same as their natural distribution zones.

As compared to MLR and MNLR, the R^2^ values of PCA-MLR and PCA-MNLR were relatively lower, only 0.439 and 0.582 respectively and far lower than that of MLR and MNLR (**Figures 8C, D**). The predictive distribution zones of all bamboo species were completely inconsistent with their natural distribution zones. showing their poor predictive abilities.

The R^2^ of the MLR-E model was only 0.670 (**Figure 9A**), which was lower than that of the MLR model (**Figure 8A**). The predictive distribution zones were lower than the actual distribution zones in the bamboo species involved in the model establishment, and quite different from the actual distribution zones in the bamboo species that were not involved in the model establishment. In MNLR-E model (**Figure 9B**), the predictive distribution zones of all bamboo species were very close to their actual distribution zones, and the R^2^ (0.891) was the highest in the four models but was still lower than that of MNLR (**Figure 8B**). Similarly, the PCA-MLR-E and PCA-MNLR-E models also showed lower R^2^ values as compared to PCA-MLR and PCA-MNLR models, (**Figures 9C, D**), which showed poor predictive abilities.

Generally, the R^2^ of the four models based on PCA analysis (PCA-MLR, PCA-MNLR, PCA-MLR-E, PCA-MNLR-E) were generally low, which did not exceed 0.6 (**Figures 8C, D**, **9C, D**). The R^2^ values of the models based on the multiple linear regression (MLR, MLR-E) were higher than those models based on PCA, but also was only about 0.7 (**Figure 8A** and **Figure 9A**). The models based on multiple nonlinear regression analysis (MNLR, MNLR-E) had the highest R^2^ (**Figures 8B**, **9B**), among which the R^2^ values of MNLR model was the highest in the eight models, implying that this model had the optimal predictive ability and could be used to predict the northernmost distribution zone of bamboos.

## Discussion

In China, most bamboo species were mainly distributed in the southern regions (Luo et al., 2019), and only a few species could be grown in the cold northern regions. Bamboo plays an important role in production and life (Scheba et al., 2017; Wang et al., 2018). This was also the driving force for the cross-regional introduction of bamboos. The longtime low temperature climate was the key limiting factor for the survival of the introduced plants in the north area, and often caused severe death of bamboos (Sun et al., 2002). Therefore, the successful bamboo introduction was mainly dependent on the selection of bamboos with high low-temperature tolerant ability.

As the main site of photosynthesis, the leaf easily suffered injuries from the low temperature climates. Hence most previous studies on cold-tolerant bamboo species were mainly focused on the morphology, anatomy and physiology of leaves (Borowski et al., 2022; Wang et al., 2022). In this study, the influences of low temperature on the leaf photosynthesis in different bamboo species in winter were compared, and the leaf morphological and anatomical characteristics of different bamboos that adapted to the low temperature regions were also analyzed. The leaf indicators related to bamboo distributions with low variability in different seasons and regions were screened, so as to be used to establish the accurate mathematical model for the distribution prediction of bamboos. It could provide fine references for the bamboo introduction and management.

### Overwintering performance and physiological changes of bamboo leaves

Low temperature not only affected the growth and development of bamboos, but also changed their external appearances. The morphological characteristics of bamboo leaves could directly reveal their environmental adaptability, which could also be used as an intuitive and convenient method for the selection of bamboos with high abilities in low temperature tolerance (Cai, 2015). Different bamboo species showed different abilities in low temperature tolerance according to their suffered injuries during winter season in the same distribution site. According to the field observations, the bamboo species of *Fargesia*, *Phyllostachys, Bashania* and some species of *Bambusa* genera showed relatively strong ability in low temperature tolerance, while those of *Dendrocalamus* species showed significantly low cold or freezing tolerant ability, and suffered severe injuries in the bamboo garden in Kunming City. In general, the sympodial bamboos suffered more severe injuries from low temperature as compared to the monopodial bamboos. This could also explain the reason why no *Dendrocalamus* species could be introduced into the central or northern areas of China.

Low temperature could directly affect the structure and activity of the photosynthetic apparatus and other physiological processes, and thereby decreased the photosynthesis (Xu, 2009). Barros et al. (1997) noticed that the low night temperature lead the partial stomatal closure of *Coffea arabica* at the next day. Siddique et al. (2000) presented that the drought conditions could also cause a decrease in stomatal conductance. The continuous water supply via transpiration was essential for the leaf photosynthesis. The leaf photosynthetic rates and transpiration rates of all bamboo species in Kunming also declined constantly with the decrease of temperature from November to the next February. Moreover, the period from November to February of the next year was just the local dry season (Zhu et al., 2016), which further decreased the transpiration and photosynthesis rates of leaves. Generally, the net photosynthesis rates and transpiration rates of the bamboos with low-temperature tolerant abilities decreased more greatly with decreasing temperature. This might be because they were more susceptible to the environmental changes as compared to those with high abilities to resist low temperature.

The diurnal variation curves of photosynthetic and transpiration rates showed different trends in different bamboo species. Some species showed one single peak in the diurnal variation curves, such as *Bambusa multiplex*, *B. textilis*, and *Phyllostachys edulis*, while others showed the double peaks, such as *Bambusa multiplex* f. *alphonso-karri*, *B. rigida*, *Chimonocalamus pallens*, *Dendrocalamus brandisii* and so on. The variation curves of net photosynthetic rates and transpiration rates varied with light and temperature (Alam et al., 2005). The double peak curves was usually caused by high light intensity at noon (Xu et al., 2014). Hence, the single peak curves might reveal that these bamboo species were more adaptive to the high light intensity and low temperature of Kunming in winter.

The moisture content in bamboo leaves decreased with months, which was also associated with the constant decrease of temperature and prolonged drought season. A similar result was also reported in the leaves of *Brassica napus* (Xu et al., 2022). It was mainly because low temperature stress was often accompanied by water stress, which affected the absorption of water by roots and decreased the moisture content of leaves (Zhao et al., 2012). The prolonged dry season and continuous decrease in rainfall further decreased the moisture content of leaves, which was similar to the results reported by Siddique et al. (2000) and Flexas and Medrano (2002).

The accumulation of soluble sugar could increase the cytoplasmic concentration, reduced the freezing point to prevent cytoplasmic dehydration, decreased the damage of low temperature, and improved the ability in low temperature tolerance (Shahba et al., 2003; Wang, 2017). The soluble sugar contents increased constantly as temperature decreased with months in the leaves of all bamboo species of Kunming site. This implied the bamboos increased their resistance to the low temperature by accumulating a large amount of soluble sugar contents in leaves. The leaves of monopodial bamboos showed more soluble sugar content as compared to those of other bamboo species, which suggested that the low temperature tolerant abilities of bamboo leaves were completely consistent with their soluble sugar contents. The bamboo species with strong abilities to resist low temperature would accumulate more soluble sugars in their leaves in winter seasons.

Starch was hydrolyzed into soluble sugar contents in leaves to improve their low temperature resistance when temperature dropped (Zhang et al., 2014; Liang et al., 2021). Zhang et al. (2014) found that the size and number of starch grains in the leaves of Neosinocalamus affinis decreased after low temperature treatment.The starch content decreased slightly in Dec in leaves of all bamboos but with the constantly increasing soluble sugar contents, which implied the increasing ability resistant to low temperature. This was also in agreement with the result reported by Zhang et al. (2014) that starch hydrolysis can produce soluble sugar in time when chloroplast photosynthesis is reduced to reduce stromal water potential, thereby protecting cells from water loss or even plasmolysis.

### Morphological and anatomical differences in leaves of different bamboo species during winter seasons, and correlations with their northmost distribution zones

Leaves were the vegetative organs exposed to the air, and their external appearance and internal anatomical structure were easily affected by the environment conditions, which could best reflect the adaptability of plants to the ecological environment (Tian et al., 2016). Leaf anatomical characteristics were closely related to their cold tolerant abilities (Huner et al., 1981). Hence, it was important to screen out the inherent traits that directly decided the low temperature tolerant abilities and the distribution range of bamboos from so many morphological and anatomical characteristics, which was the result of the long-term adaptation to the environment, not a result of the short-term environmental responses.

Leaf morphology had been demonstrated to link with climate and varied within species (Guerin et al., 2012). Royer (2012) also reported that leaf size and shape was related to climate. Guerin et al. (2012) reported that leaf size was associated with latitude and altitude.By comparison, it was concluded that the bamboo species distributed in tropical regions usually showed larger leaves (leaf area, leaf length, leaf width) with thicker epidermis. However, the ratio of cuticle to leaf area and the density of leaf vein of bamboos in low-temperature regions were larger. These results revealed that the leaf size of bamboos was closely related to environmental temperature. Correlation analysis showed that the bamboos with larger leaves were distributed in more southward zones, implying weaker abilities in low temperature tolerance. For leaf length/leaf area and leaf width/leaf area, the higher values, the more southern distribution and weaker abilities in low temperature tolerance. On the contrary, bamboos with smaller ratios of leaf length/leaf thickness and leaf width/leaf thickness were distributed more northern regions and implied stronger abilities in low temperature tolerance. Tian et al. (2021) reported similar results that the leaf length of *Pinus tabuliformis* decreased while the leaf thickness increased with altitude, which implied that the short roundish leaves were more adaptable to the high temperature and drought environment of high altitude compared with the large leaves. Liu et al. (2020) also considered that the leaf length and width of three plant species decreased but leaf thickness increased constantly with the air temperature decreased abruptly. Therefore, the closer the bamboo leaves were to the short rod shape, the stronger the tolerant abilities in low temperature environment.

Leaf vein densities of bamboos were also shown to be related to their distribution zones. The bamboo species distributed in the northern regions showed more leaf veins as compared to those distributed in the southern regions. The higher the vein density was, the more northern region the bamboo was distributed in. Miao et al. (2004) also reported the same conclusion in wheat. This was because the precipitation also reduced in the northern region of China except for the decrease of temperature. Smaller leaves with higher vein density were more tolerant to the draught by providing more “superhighways” for water transport (Scoffoni et al., 2011; Sack et al., 2012). Correlation analyses also indicated that the leaf vein density was significantly correlated with the northernmost distribution zones of bamboos. Therefore, the bamboo with larger leaf vein densities might be better adapted to the low-temperature and arid environment of northern regions.

Leaf anatomical traits displayed evolutionary adaptive changes to suit the surrounding environment (Liu et al., 2020). The cuticle had a protective effect on leaf cells and could reduce or avoid damage to plants (Sturaro et al., 2005). The cuticle was composed of the cutin polymer matrix, and cold stress led to increase in the wax content of the cuticle (He et al., 2019). However, the cuticle of bamboo leaves did not show strong correlations with the distribution zones of bamboos, while the ratio of the cuticle to leaf area reached significant correlations with the distribution of bamboos, particularly, the ratio of the adaxial cuticle to leaf area. The bamboos with a larger ratio of cuticle thickness/leaf area could distributed the further north zones.

The epidermis was another protective tissue beside the cuticle. Li and Bao (2014) found that the leaf epidermis thickness increased with the increase of altitude and the decrease of temperature. Xu et al. (2009) found thicker adaxial epidermis of leaves under low temperature conditions. On the contrary, the present work found that the leaf epidermis, especially the adaxial epidermis, showed significant and positive correlations with the distributions of different bamboo species. It was mainly because the leaves of tropical bamboo species usually had larger size, thicker leaf thickness and thicker epidermis than those of temperate bamboo species. In addition, the temperate bamboos showed lower proportions of adaxial epidermis thickness than the tropical bamboos. The correlation analysis showed that the epidermis thickness of bamboo leaves correlated significantly and positively with the size of leaves. Similarly, the ratios of adaxial epidermis thickness/leaf thickness and total epidermis thickness/leaf thickness also showed significant and positive correlations with bamboo distribution zones. Therefore, the indicators could be used for the prediction of bamboo distribution zones.

Mesophyll was the main place for the photosynthesis of leaves (Zhang et al., 2021). Gorsuch et al. (2010) treated plants at low temperatures and found that mesophyll cells became larger and mesophyll thickness were increased. However, the mesophyll thickness also did not show significant correlations with the distributions of different bamboo species, which was similar to the leaf thickness. Similarly, the ratios of mesophyll thickness/leaf thickness, mesophyll thickness/leaf area, and leaf thickness/leaf area also did not significantly correlate with the distribution zones. Therefore, these indicators could not be used as prediction indicators for the prediction of bamboo distribution.

### Variation of morphological and anatomical indicators of leaves with seasons and regions

Leaf morphology and anatomy changed with seasonal adjustment, which was mainly caused by environmental changes, such as light intensity, humidity and temperature (Kim et al., 2019; Mendes et al., 2022; Walasek et al., 2022). The growth dynamics of leaves also reflected seasonal changes (Hubálková et al., 2017). As analyzed the leaf morphological and anatomical variability of 18 bamboo species between seasons, the results showed that the morphological and anatomical characteristics of bamboo leaves changed with seasons. Most indicators related to leaf area were variable and unstable, while the indicators related to leaf thickness and the leaf length/leaf width ratios were stable between seasons. This indicated that the basic shape of bamboo leaves had been fully adapted to the local environment and did not change with seasons, except the leaf size.

Dombroski et al. (2011) reported that the optimal leaf structure could be determined depending on seasons, and summer leaves had a higher stomatal density than winter leaves. Ferris et al. (1996) reported the same result. The length of new leaves (Smith and Nobel, 1977) and the leaf structures of trees were affected by seasonal changes in water supply (Kloeppel et al., 1993). Mendes et al. (2022) found that the seasonality of precipitation affected the leaf size and anatomical structure. It was also noticed that the bamboo leaves were larger in summer than in winter, as well as their anatomical components, such as tadaxial and abaxial epidermis cuticle, which were also thicker in summer. Cuticle thickening could contribute to light reflection, which helped leaves protect themselves from excessive radiation (Chazdon and Kaufmann, 1993). Kunming City had more precipitation in the rainy season in summer and less precipitation in the dry season in winter. Plants were likely to exhibit their best morphological traits in the optimal circumstance, and that was the maximum photosynthetic activity was observed in the rainy season with a larger leaf area than in the dry season (Mendes et al., 2022). Therefore, a larger bamboo leaf was shown in summer but small size of leaves in winter.

In addition to seasons, different regions also played an important role in morphological and anatomical changes of leaves. Leaf morphology and anatomical traits could be significantly influenced by climate (Tian et al., 2016). Beyschlag and Zotz (2017) founded that the structural characteristics of leaves were related to habitat differences. Zhao et al. (2019a) reported that the leaf stomatal density showed a decrease trend In high altitude environment. Gielwanowska et al. (2005) reported that the leaves of plants grown in dry, nutrient-deficient and wind-exposed areas were short and small, and the cell walls were thick.

Similar to the variability of bamboo leaves with seasons, the indicators related to leaf area in the same bamboo species also significantly varied with regions, which showed an increased trend from the northern regions to the southern regions. The leaves of bamboos often showed larger size and more stomata in the tropic region. However, the indicators related to leaf thickness were relatively stable and did not vary significantly with regions. Luomala et al. (2005) reported that cell differentiation increased under relatively high temperature conditions, which resulted in increased stomatal density. At lower-temperature regions, the leaf length/width showed larger values, whereas the leaf length/leaf thickness and leaf width/leaf thickness showed smaller values. This implied that the leaves were closer to the “short rod” shape in lower-temperature regions, so as to better resist the low temperature stress. Additionally, the ratio of mesophyll thickness to leaf thickness was lower whereas the ratios of cuticle and epidermis to leaf thickness were larger in the temperate regions than in the tropical regions. This implied the proportion of cuticle and epidermis in leaves would constantly increase from the southern regions to the northern regions in the same bamboo species. The ratios of leaf length, leaf width, leaf thickness, adaxial cuticle and mesophyll thickness to leaf areas were larger in the northern regions than in the southern regions, whereas the ratios of other indexes to leaf area did not change regularly with bamboo distribution regions. Overall, plants could adjust their leaf anatomy in response to changing environmental conditions (Tian et al., 2016).

It can be concluded that the leaf areas and the ratios of all indicators to leaf area varied greatly with environmental changes, which were mainly caused by the climate changes from the changes of regions and seasons. This indicated that leaf area had stronger plasticity under different environmental conditions and hence was not suitable for the prediction model establishment. The ratios of length/width, leaf length/leaf thickness, and leaf width/leaf thickness showed medium variability (CV:15-35%) with regions, which might be because bamboo leaves could make some adjustments to adapt to different environments. Moreover, the variability of these indicators were higher with regions than with seasons, and this indicated that the leaf size of bamboos was more susceptible to the influences of regions than those of seasons.

### Key indicators selection and establishment of mathematical prediction models

During the establishment of mathematical prediction models, the stable indicators were more essential and could better reveal their adaptability to stress, which were usually the results of long time of domestication or natural selection. However, plants were exquisitely sensitive to their surroundings (Kohchi et al., 2001). The physiological indicators of leaves were easily affected by the environment, which usually revealed the emergency responses to stress, and changed dramatically within months and even at different time at one day. Therefore the leaf physiological indicators could not be used in the establishment of mathematical models because of their high instability. Similarly, these morphological and anatomical indicators that were easily affected by the distribution regions and seasons should also be excluded during model establishment, so as to increase the accuracy of models. As a result, the indicators including leaf vein density, leaf length, leaf width, upper epidermis thickness, epidermis thickness, leaf length/leaf thickness, leaf width/leaf thickness, upper epidermis thickness/leaf thickness, total epidermis thickness/leaf thickness, leaf area/leaf length, leaf area/leaf width, upper cuticle/leaf area, total cuticle thickness/leaf area were suitable for the establishment of mathematical prediction models according to their high correlations with distributions and low variabilities (CV<35%).

As for the establishment of mathematical prediction models, Tackenberg (2007) used linear regression to fit the above ground fresh biomass, dry biomass, and dry matter content of forage grass with growth-related traits, and the fitting degree was higher than 0.85, which showed a good linear relationship with growth-related traits. In this study, the R^2^ values of MLR and MLR-E were 0.740 and 0.670 respectively, lower than those of MNLR and MNLR-E, which were 0.939 and 0.891 respectively. This indicated that the relationship between indicators and bamboo distributions should be a nonlinear relationship.

Because of the large number of indicators, the classical method of dimensionality reduction of data was PCA in previous studies(Artoni et al., 2018). However, the prediction ability of the model established by PCA was poor because of their low R^2^ values, which were far lower than those of MNLR and MNLR-E. This might be because PCA deleted redundant data, but it inevitably deleted some useful data (Zhao et al., 2019b). Therefore, the principal component analysis was not suitable for the establishment of mathematical models for the distribution prediction of bamboos.

Additionally, the re-established models analysis with the exclusion of the indicators with extremely weak correlation coefficients showed lower R^2^ values as compared to the models with all indicators. This implied that the distributions of bamboos were influenced not only by the indicators with high correlation coefficients, but also by the indicators with weak correlation coefficients. The optimal distribution prediction of model was the multiple nonlinear regression (MNLR), i.e., y = 259.063 − 0.018x _1_ − $0.0000007137{\text{x}}_{2}^{3}$ + 0.147x_2_ − $0.031{\text{x}}_{3}^{2}$ + 266.417x_3_ + $0.099{\text{x}}_{4}^{3}$ − $3.5741{\text{x}}_{4}^{2}$ + 35.065x_4_ − $0.063{\text{x}}_{5}^{3}+3.764{\text{x}}_{5}^{2}$ − 69.234x_5_ − $9.959{\text{x}}_{6}^{3}+40.43{\text{x}}_{6}^{2}$ − 48.269x_6_ + $1061.43{\text{x}}_{7}^{3}-534.01{\text{x}}_{7}^{2}$ + 82.578x_7_ + $7112.004{\text{x}}_{8}^{3}-5283.989{\text{x}}_{8}^{2}$ + 2023.526x_8_ + $1701.552{\text{x}}_{9}^{2}$ − 1301.342x_9_ − 187.3881.594+x10−4.8330.036+x11+17117685.84x123−508496.037x122+1917.935x12+0.03x13 . The verification test also supported this result. Moreover, different types of bamboos were involved in the model establishment, which further increase its prediction accuracy. Hence, the model was also applicable for the induction prediction in different bamboo types.

However, it was difficult to predict the specific and precise introduction range of bamboos according to the prediction models during the cross regional introduction of bamboos. This was because of a significant association between plant species and microclimate (Sánchez-Reyes et al., 2021). Therefore, the air temperature and precipitation caused by altitudes should also be fully taken into consideration during bamboo introduction. Moreover, some bamboo species could tolerate low temperature, but could not resist the strong drought stress. Wang et al. (2009) reported that *Fargesia yunnanensis* had low temperature tolerant abilities, but usually grew better in high humidity and could not tolerate the long-term drought. Hence, it was essential to investigate and compare the climates between the origin regions and newly introduced regions, and then to combine the prediction results of mathematical models for comprehensive evaluation before introduction. Additionally, small-scale introduction and acclimatization should also be carried out. The mathematical model also needed to add more bamboo species to adjust the parameters, so as to further increase the prediction accuracy and serve the development of the bamboo industry.

## Data availability statement

The original contributions presented in the study are included in the article/**Supplementary Material**, further inquiries can be directed to the corresponding author/s.

## Author contributions

SW: Funding acquisition, Methodology, Resources, Supervision, Writing – review & editing. YW: Data curation, Formal Analysis, Investigation, Methodology, Validation, Visualization, Writing – original draft. JL: Data curation, Formal Analysis, Investigation, Methodology, Writing – review & editing. LY: Formal Analysis, Investigation, Writing – review & editing. ZL: Formal Analysis, Resources, Validation, Writing – review & editing. HL: Formal Analysis, Resources, Validation, Writing – review & editing. JZ: Resources, Writing – review & editing. SL: Resources, Writing – review & editing. MY: Resources, Writing – review & editing. ZC: Resources, Writing – review & editing. HS: Resources, Writing – review & editing.

## Supplementary material

Supplementary Figure 1 Daily changes of photosynthetically active radiation intensity in different months.

Supplementary Figure 2 Anatomical observation of leaves of each bamboo species. UC:adaxial (upper) cuticle, LC: abaxial (lower) cuticle, UE: adaxial (upper) epidermis, LE, abaxial (lower) epidermis, ME: mesophyll. **(A)** *Dendrocalamus brandisii*. Bar=100μm. **(B)** *Dendrocalamus farinosus*. Bar=100μm. **(C)** *Dendrocalamus hamiltonii*. Bar=100μm. **(D)** *Neosinocalamus affinis*. Bar=100μm. **(E)** *Neosinocalamus affinis* f. *viridiflavus*. Bar=100μm. **(F)** *Bambusa distegia*. Bar=100μm. **(G)** *Bambusa multiplex*. Bar=100μm. **(H)** *Bambusa multiplex* f. *alphonso-karri*. Bar=100μm. **(I)** *Bambusa multiplex* f. *fernleaf*. Bar=100μm. **(J)** *Bambusa rigida*. Bar=100μm. **(K)** *Bambusa textilis*. Bar=100μm. **(L)** *Bambusa tuldoides*. Bar=100μm. **(M)** *Bambusa ventricosa*. Bar=100μm. **(N)** *Chimonocalamus pallens*. Bar=100μm. **(O)** *Drepanostachyum scandeus*. Bar=100μm. **(P)** *Fargesia fractiflexa*. Bar=100μm. **(Q)** *Fargesia fungosa*. Bar=100μm. **(R)** *Fargesia yunnanensis*. Bar=100μm. **(S)** *Bashania fargesii*. Bar=100μm. **(T)** *Pseudosasa japonica*. Bar=100μm. **(U)** *Sasa fortunei*. Bar=100μm. **(V)** *Sasa pygmaea*. Bar=100μm. **(W)** *Qiongzhuea tumidinoda*. Bar=100μm. **(X)** *Indocalamus decorus*. Bar=100μm. **(Y)** *Indocalamus tessellatus*. Bar=100μm. **(Z)** *Pleioblastus amarus*. Bar=100μm. (AA) *Phyllostachys aurea*. Bar=100μm. (AB) *Phyllostachys aureosulcata* f. *spectabilis*. Bar=100μm. (AC) *Phyllostachys bissetii.* Bar=100μm. (AD) *Phyllostachys edulis*. Bar=100μm. (AE) *Phyllostachys mannii*. Bar=100μm. (AF) *Phyllostachys nigra*. Bar=100μm. (AG) *Phyllostachys nigra* var. *henonis.* Bar=100μm. (AH) *Phyllostachys vivax* f. *aureocaulis*.

Supplementary Figure 3 Changes of leaf morphological indicators of 18 bamboo species with different seasons. **(A)** Leaf area. **(B)** Leaf vein density. **(C)** Stomatal density. **(D)** Leaf length. **(E)** Leaf width. **(F)** Leaf length/leaf width.

Supplementary Figure 4 Changes of leaf anatomical indicators of 18 bamboo species with different seasons. **(A)** Leaf thickness. **(B)** Adaxial cuticle thickness. **(C)** Abaxial cuticle thickness. **(D)** Total cuticle thickness. **(E)** Adaxial epidermis thickness. **(F)** Abaxial epidermis thickness. **(G)** Total epidermis thickness. **(H)** Mesophyll thickness.

Supplementary Figure 5 The changes of ratios of morphological and anatomical indicators to leaf thickness of 18 bamboo species with different seasons. **(A)** Leaf length/leaf thickness. **(B)** Leaf width/leaf thickness. **(C)** Adaxial cuticle thickness/leaf thickness. **(D)** Abaxial cuticle thickness/leaf thickness. **(E)** Total cuticle thickness/leaf thickness. **(F)** Adaxial epidermis thickness/leaf thickness. **(G)** Abaxial epidermis thickness/leaf thickness. **(H)** Total epidermis thickness/leaf thickness. **(I)** Mesophyll thickness/leaf thickness.

Supplementary Figure 6 The changes of ratios of leaf morphological and anatomical indicators to leaf area of 18 bamboo species with different seasons. **(B)** Leaf width/leaf area. **(C)** Leaf thickness/leaf area. **(D)** Adaxial cuticle thickness/leaf area. **(E)** Abaxial cuticle thickness/leaf area. **(F)** Total cuticle thickness/leaf area. **(G)** Adaxial epidermis thickness/leaf area. **(H)** Abaxial epidermis thickness/leaf area. **(I)** Total epidermis thickness/leaf area. **(J)** Mesophyll thickness/leaf area.

Supplementary Figure 7 Changes of morphological indicators of bamboo leaves of three bamboo species with different regions. **(A)** Leaf area. **(B)** Leaf vein density. **(C)** Stomatal density. **(D)** Leaf length. **(E)** Leaf width. **(F)** Leaf length/leaf width.

Supplementary Figure 8 Changes of anatomical indicators of bamboo leaves of three bamboo species with different regions. **(A)** Leaf thickness. **(B)** Adaxial cuticle thickness. **(C)** Abaxial cuticle thickness. **(D)** Total cuticle thickness. **(E)** Adaxial epidermis thickness. **(F)** Abaxial epidermis thickness. **(G)** Total epidermis thickness. **(H)** Mesophyll thickness.

Supplementary Figure 9 Changes of ratios of leaf morphological and anatomical indicators to leaf thickness of three bamboo species with different regions. **(A)** Leaf length/leaf thickness. **(B)** Leaf width/leaf thickness. **(C)** Adaxial cuticle thickness/leaf thickness. **(D)** Abaxial cuticle thickness/leaf thickness. **(E)** Total cuticle thickness/leaf thickness. **(F)** Adaxial epidermis thickness/leaf thickness. **(G)** Abaxial epidermis thickness/leaf thickness. **(H)** Total epidermis thickness/leaf thickness. **(I)** Mesophyll thickness/leaf thickness.

Supplementary Figure 10 Changes of ratios of morphological and anatomical indicators to leaf area of three bamboo species with different regions. **(A)** Leaf length/leaf area. **(B)** Leaf width/leaf area. **(C)** Leaf thickness/leaf area. **(D)** Adaxial cuticle thickness/leaf area. **(E)** Abaxial cuticle thickness/leaf area. **(F)** Total cuticle thickness/leaf area. **(G)** Adaxial epidermis thickness/leaf area. **(H)** Abaxial epidermis thickness/leaf area. **(I)** Total epidermis thickness/leaf area. **(J)** Mesophyll thickness/leaf area.
