# Supplementary material for: Overwintering performance of bamboo leaves, and establishment of mathematical model for the distribution and introduction prediction of bamboos
Source: Front Plant Sci. 2023 Sep 8;14:1255033. doi: 10.3389/fpls.2023.1255033 (PMC10515091; doi:10.3389/fpls.2023.1255033)
Supplement: Supplementary file 1 [file Table_1.docx]

Table S1 The ratio of leaf morphological and anatomical indicators to leaf thickness of different bamboo species

| Bamboo species | Leaf length/  leaf thickness | Leaf width/  leaf thickness | Adaxial cuticle thickness/  leaf thickness | Abaxial cuticle thickness/  leaf thickness | Total cuticle thickness/  leaf thickness | Adaxial epidermis thickness/  leaf thickness | Abaxial epidermis thickness/  leaf thickness | Total epidermis thickness/  leaf thickness | Mesophyll thickness/  leaf thickness |
| --- | --- | --- | --- | --- | --- | --- | --- | --- | --- |
| *Dendrocalamus brandisii* | 2.4899±0.6385b | 0.4087±0.1212b | 0.0336±0.0088h | 0.0320±0.0709a | 0.0656±0.0730bc | 0.0781±0.0221c | 0.0541±0.0147d | 0.1322±0.0332c | 0.7542±0.0603p |
| *Dendrocalamus farinosus* | 1.425±0.1406cd | 0.1997±0.0141fg | 0.0272±0.0039kl | 0.0183±0.0039mn | 0.0455±0.0056klm | 0.0698±0.0201a | 0.0366±0.0059b | 0.1064±0.0191a | 0.7950±0.0242n |
| *Dendrocalamus hamiltonii* | 2.1317±0.517ab | 0.3252±0.0549a | 0.0246±0.0035e | 0.0184±0.0041d | 0.0431±0.0054ef | 0.0776±0.0250mno | 0.0432±0.0154hi | 0.1208±0.0384mno | 0.7744±0.0584o |
| *Neosinocalamus affinis* | 2.065±0.4182o | 0.2627±0.0528opq | 0.0403±0.0084lm | 0.0290±0.0063m | 0.0694±0.0110klm | 0.1174±0.0202qr | 0.0719±0.0154o | 0.1893±0.0277rs | 0.6861±0.0505ij |
| *Neosinocalamus affinis* f. *viridiflavus* | 1.5254±0.1222m | 0.1495±0.0111jk | 0.0333±0.0070a | 0.0250±0.0052d | 0.0583±0.0097a | 0.0972±0.0138c | 0.0575±0.0075a | 0.1547±0.0168b | 0.7431±0.0293hij |
| *Bambusa distegia* | 0.668±0.1037j | 0.0895±0.012j | 0.0373±0.0075n | 0.0251±0.0062p | 0.0624±0.0109nop | 0.1206±0.0186ijkl | 0.0572±0.012p | 0.1778±0.0253opq | 0.7229±0.0463e |
| *Bambusa multiplex* | 1.2139±0.1942mn | 0.1331±0.0196q | 0.0305±0.0082s | 0.0230±0.0052p | 0.0535±0.0110opq | 0.1156±0.0186hi | 0.0630±0.0148ij | 0.1786±0.0284jk | 0.7276±0.0399hi |
| *Bambusa multiplex* f. *alphonso-karri* | 0.9537±0.1852m | 0.1238±0.0257lm | 0.0270±0.0061rs | 0.0219±0.0058q | 0.0489±0.0096opq | 0.1124±0.0202s | 0.0592±0.0126q | 0.1716±0.0271u | 0.7258±0.0352a |
| *Bambusa multiplex* f. *fernleaf* | 0.4145±0.1561j | 0.0704±0.0199k | 0.0275±0.0071lm | 0.0218±0.0060c | 0.0493±0.0109ghi | 0.1210±0.0198d | 0.0661±0.0129g | 0.1870±0.0257e | 0.7096±0.0382lm |
| *Bambusa rigida* | 0.7392±0.0217k | 0.1284±0.0054nop | 0.0251±0.0035s | 0.0196±0.0030g | 0.0447±0.0050lmn | 0.0882±0.0079hi | 0.0472±0.0078klm | 0.1355±0.009kl | 0.7702±0.0246kl |
| *Bambusa textilis* | 1.1187±0.2411i | 0.1219±0.0079gh | 0.0267±0.0063e | 0.0196±0.0056e | 0.0463±0.0102f | 0.0975±0.0151hi | 0.0498±0.0106gh | 0.1473±0.0215ij | 0.7408±0.0361ij |
| *Bambusa tuldoides* | 0.7498±0.7606gh | 0.0829±0.0839kl | 0.0286±0.0067i | 0.0220±0.0051hi | 0.0506±0.0098jk | 0.1115±0.0198hi | 0.0581±0.0118kl | 0.1696±0.0249kl | 0.7109±0.0308ij |
| *Bambusa ventricosa* | 1.4823±0.1833lm | 0.2041±0.0285mno | 0.0301±0.0076opq | 0.0286±0.0509q | 0.0587±0.0517opq | 0.1201±0.0194op | 0.0667±0.0130mn | 0.1868±0.0257q | 0.7088±0.0551de |
| *Chimonocalamus pallens* | 1.21±0.1086s | 0.1289±0.0096s | 0.0315±0.0060d | 0.0209±0.0055e | 0.0525±0.0100de | 0.1456±0.0133b | 0.0703±0.0106f | 0.2159±0.0185b | 0.7234±0.0337kl |
| *Drepanostachyum scandeus* | 1.3443±0.0865q | 0.2043±0.0209r | 0.0259±0.0069f | 0.0208±0.0039g | 0.0467±0.0088g | 0.0781±0.0124jklm | 0.0481±0.0106n | 0.1262±0.0176no | 0.7706±0.0288kl |
| *Fargesia fractiflexa* | 1.6497±0.0622op | 0.1466±0.0096mno | 0.0266±0.0050w | 0.0224±0.0054s | 0.0489±0.0071r | 0.0729±0.0113r | 0.0580±0.0085op | 0.1309±0.0139t | 0.7906±0.0258b |
| *Fargesia fungosa* | 2.3606±0.1899rs | 0.2061±0.0233r | 0.0362±0.0074op | 0.0301±0.0071m | 0.0663±0.0114mn | 0.1049±0.0215d | 0.0714±0.0163f | 0.1764±0.0330e | 0.7350±0.0352mn |
| *Fargesia yunnanensis* | 1.7199±0.2377l | 0.1878±0.025mno | 0.0377±0.0090jk | 0.0288±0.0071ijk | 0.0665±0.0138jkl | 0.0894±0.0143hi | 0.0607±0.0145hi | 0.1501±0.0228jk | 0.7395±0.0412k |
| *Bashania fargesii* | 1.3956±0.2753op | 0.1658±0.0205pq | 0.0386±0.0101s | 0.0239±0.0067q | 0.0625±0.0141pq | 0.0776±0.0159qr | 0.0560±0.0106lmn | 0.1336±0.0222r | 0.7486±0.0491cd |
| *Pseudosasa japonica* | 1.5485±0.1488t | 0.1811±0.0183t | 0.0306±0.0062v | 0.0180±0.0039r | 0.0486±0.0083r | 0.0865±0.0142lmno | 0.0506±0.0102lmn | 0.1371±0.0194nop | 0.7593±0.0268bc |
| *Sasa fortunei* | 0.6089±0.1783p | 0.0748±0.0176mnop | 0.0286±0.0060no | 0.0201±0.0047hij | 0.0487±0.0087klm | 0.0791±0.0126hij | 0.0593±0.0112kl | 0.1384±0.0189kl | 0.7625±0.0329n |
| *Sasa pygmaea* | 0.5503±0.0959o | 0.0847±0.0169mnop | 0.0325±0.0073h | 0.0211±0.0053g | 0.0536±0.0106hi | 0.0868±0.0149fg | 0.0583±0.0111ij | 0.1451±0.0209ghi | 0.7526±0.0336o |
| *Qiongzhuea tumidinoda* | 1.4814±0.255e | 0.1218±0.0177d | 0.04360±0.0140g | 0.0295±0.0084l | 0.0731±0.0204ij | 0.1062±0.0194r | 0.0702±0.0143o | 0.1764±0.0262st | 0.7040±0.0477e |
| *Indocalamus decorus* | 1.9703±0.2855c | 0.3493±0.0754de | 0.0363±0.0063qr | 0.0240±0.0051h | 0.0603±0.0098lmn | 0.0790±0.0103no | 0.0544±0.0094lmn | 0.1334±0.0149pq | 0.7547±0.0293b |
| *Indocalamus tessellatus* | 1.5181±0.1765j | 0.2405±0.0442jk | 0.0354±0.0105r | 0.0244±0.0073n | 0.0598±0.0162no | 0.0883±0.0195ef | 0.0544±0.0117g | 0.1427±0.0268f | 0.7451±0.0396ij |
| *Pleioblastus amarus* | 1.183±0.2274d | 0.1409±0.0303c | 0.0352±0.0094ij | 0.0212±0.0063mn | 0.0564±0.0133klm | 0.0875±0.0152klmn | 0.0621±0.0174jk | 0.1496±0.0269mn | 0.7445±0.0587de |
| *Phyllostachys aurea* | 0.8496±0.1635fg | 0.1283±0.023i | 0.0341±0.0098g | 0.0240±0.0067kl | 0.0581±0.0147ij | 0.0787±0.0147ef | 0.0556±0.0127hi | 0.1343±0.0234fg | 0.7491±0.0449ij |
| *Phyllostachys aureosulcata* f. *spectabilis* | 0.9494±0.1765ef | 0.1342±0.0206fg | 0.0343±0.0089b | 0.0229±0.0054b | 0.0572±0.0121ab | 0.0832±0.0138d | 0.0561±0.0104c | 0.1393±0.0193d | 0.7448±0.0370mn |
| *Phyllostachys bissetii* | 0.799±0.0983ef | 0.1231±0.0164hi | 0.0320±0.0071m | 0.0214±0.0052o | 0.0534±0.0102mn | 0.0811±0.0139pq | 0.0556±0.0102n | 0.1367±0.0190r | 0.7521±0.0377fg |
| *Phyllostachys edulis* | 0.9514±0.2271ghi | 0.1238±0.0402j | 0.0386±0.0094t | 0.0271±0.0069mn | 0.0656±0.0135opq | 0.0991±0.0173ghi | 0.0658±0.0144lmn | 0.1649±0.0267kl | 0.7175±0.0470e |
| *Phyllostachys mannii* | 0.9045±0.2231qr | 0.1240±0.034r | 0.0351±0.0082lm | 0.0235±0.0065o | 0.0586±0.0125mn | 0.0737±0.0127jklm | 0.0520±0.0100hi | 0.1257±0.0189lm | 0.7543±0.0383f |
| *Phyllostachys nigra* | 0.9143±0.1873ef | 0.1205±0.0235j | 0.0327±0.0077u | 0.0232±0.0063p | 0.0559±0.0117q | 0.0913±0.0171e | 0.0622±0.0156o | 0.1535±0.0285hij | 0.7413±0.0368ij |
| *Phyllostachys nigra* var. *henonis* | 0.771±0.0748no | 0.103±0.0129mn | 0.0319±0.0075c | 0.0211±0.0055f | 0.0530±0.0108cd | 0.0807±0.0148hijk | 0.0543±0.0107d | 0.1350±0.0205fgh | 0.7485±0.0355gh |
| *Phyllostachys vivax* f. *aureocaulis* | 0.98±0.1705hi | 0.1148±0.0179ef | 0.0324±0.0073f | 0.0226±0.0054jk | 0.0551±0.0104gh | 0.0829±0.0151gh | 0.0519±0.0107e | 0.1348±0.0221fg | 0.7472±0.0377ij |

Means with the same letters in each column was not significantly different (P < 0.05). Means with the different letters in each column was significantly different (P≥0.05).

Table S2 The ratio of morphological and anatomical indicators to leaf area of different bamboo species

| Bamboo species | Leaf length/leaf area | Leaf width/leaf area | Leaf thickness/leaf area | Adaxial cuticle thickness/leaf area | Abaxial cuticle thickness/leaf area | Total cuticle thickness/leaf area | Adaxial epidermis thickness/leaf area | Abaxial epidermis thickness/leaf area | Total epidermis thickness/leaf area | Mesophyll thickness/leaf area |
| --- | --- | --- | --- | --- | --- | --- | --- | --- | --- | --- |
| *Dendrocalamus brandisii* | 0.0284±0.0033i | 0.0046±0.0004de | 0.0123±0.0039d | 0.0004±0.0001e | 0.0003±0.0006c | 0.0007±0.0006c | 0.0009±0.0002b | 0.0006±0.0001c | 0.0015±0.0003b | 0.0095±0.0037d |
| *Dendrocalamus farinosus* | 0.0369±0.0056mn | 0.0052±0.0007r | 0.0259±0.0029p | 0.0007±0.0001op | 0.0005±0.0001pq | 0.0012±0.0002qr | 0.0018±0.0004l | 0.0009±0.0002lm | 0.0028±0.0008ij | 0.0206±0.0020r |
| *Dendrocalamus hamiltonii* | 0.0284±0.0040r | 0.0044±0.0003mn | 0.0137±0.0018lmno | 0.0003±0.0001k | 0.0002±0.0001lm | 0.0006±0.0001kl | 0.0010±0.0003rs | 0.0006±0.0002lm | 0.0016±0.0004mno | 0.0107±0.0021nopq |
| *Neosinocalamus affinis* | 0.0579±0.0119m | 0.0074±0.0015lm | 0.03±0.0109k | 0.0012±0.0005j | 0.0009±0.0004kl | 0.0021±0.0008jk | 0.0035±0.0013mnop | 0.0022±0.0009jk | 0.0056±0.0021jkl | 0.0206±0.0077m |
| *Neosinocalamus affinis* f. *viridiflavus* | 0.0786±0.0104ef | 0.0077±0.0009d | 0.0514±0.004ij | 0.0017±0.0004f | 0.0013±0.0003g | 0.0030±0.0006e | 0.0051±0.0012gh | 0.0030±0.0005f | 0.0080±0.0013e | 0.0382±0.0029ij |
| *Bambusa distegia* | 0.0994±0.0094q | 0.0135±0.0022pq | 0.1527±0.0300mnop | 0.0057±0.0016mno | 0.0038±0.0012p | 0.0095±0.0024pq | 0.0183±0.0047opqrs | 0.0087±0.0023mn | 0.0271±0.0063mno | 0.1101±0.0213opqr |
| *Bambusa multiplex* | 0.0841±0.0152c | 0.0093±0.0019b | 0.0718±0.0207b | 0.0022±0.0008d | 0.0017±0.0006d | 0.0038±0.0013c | 0.0083±0.0029b | 0.0046±0.0017b | 0.0129±0.0045b | 0.0521±0.0147b |
| *Bambusa multiplex* f. *alphonso-karri* | 0.0880±0.0150jk | 0.0115±0.0024ij | 0.0979±0.0359hi | 0.0026±0.0009h | 0.0021±0.0008ij | 0.0047±0.0015gh | 0.0112±0.0051kl | 0.0058±0.0024hi | 0.0170±0.0071gh | 0.0710±0.0261h |
| *Bambusa multiplex* f. *fernleaf* | 0.1827±0.0571k | 0.0335±0.0150jl | 0.5690±0.4086k | 0.0148±0.0097j | 0.0119±0.0083ij | 0.0266±0.0175ij | 0.0679±0.0481jk | 0.0365±0.0252i | 0.1040±0.0713g | 0.4091±0.3064m |
| *Bambusa rigida* | 0.0607±0.0029gh | 0.0105±0.0007ij | 0.0821±0.0036ij | 0.0021±0.0003i | 0.0016±0.0003hi | 0.0037±0.0005h | 0.0073±0.0012hi | 0.0039±0.0007gh | 0.0111±0.001f | 0.0632±0.0033jk |
| *Bambusa textilis* | 0.0707±0.0137a | 0.0078±0.0006f | 0.0636±0.0033c | 0.0017±0.0004b | 0.0012±0.0003b | 0.0029±0.0006b | 0.0062±0.0012c | 0.0032±0.0006b | 0.0094±0.0013b | 0.0472±0.004c |
| *Bambusa tuldoides* | 0.0678±0.0099l | 0.0075±0.0010o | 0.0451±0.0033lmn | 0.0015±0.0003lm | 0.0011±0.0003no | 0.0025±0.0006mno | 0.0051±0.0014opqr | 0.0028±0.0007lm | 0.0078±0.0016klmno | 0.0321±0.0027nop |
| *Bambusa ventricosa* | 0.0590±0.008j | 0.0081±0.0010hi | 0.0403±0.0073j | 0.0012±0.0004ij | 0.0012±0.0024kl | 0.0024±0.0024ij | 0.0049±0.0012kl | 0.0027±0.0007hi | 0.0075±0.0017gh | 0.0286±0.0054jk |
| *Chimonocalamus pallens* | 0.0821±0.0059fg | 0.0088±0.0005f | 0.0680±0.0031f | 0.0021±0.0004c | 0.0014±0.0004d | 0.0036±0.0007c | 0.0099±0.0010d | 0.0048±0.0007e | 0.0147±0.0011c | 0.0492±0.0035f |
| *Drepanostachyum scandeus* | 0.0538±0.0075de | 0.0081±0.0009f | 0.0404±0.0071g | 0.0011±0.0004e | 0.0008±0.0002f | 0.0019±0.0005d | 0.0032±0.0009g | 0.0020±0.0006f | 0.0052±0.0014e | 0.0310±0.0050g |
| *Fargesia fractiflexa* | 0.0824±0.0065m | 0.0073±0.0004j | 0.0499±0.0033k | 0.0013±0.0003kl | 0.0011±0.0003no | 0.0025±0.0004lmn | 0.0037±0.0008mno | 0.0029±0.0004j | 0.0065±0.0007ijk | 0.0395±0.0030lm |
| *Fargesia fungosa* | 0.0839±0.0073c | 0.0073±0.0007c | 0.0357±0.0034e | 0.0013±0.0003f | 0.0011±0.0003e | 0.0024±0.0004d | 0.0038±0.0008e | 0.0025±0.0006d | 0.0063±0.0012c | 0.0262±0.0026e |
| *Fargesia yunnanensis* | 0.0626±0.0065hi | 0.0068±0.0008g | 0.0371±0.0066ij | 0.0014±0.0004h | 0.0011±0.0003h | 0.0025±0.0006g | 0.0033±0.0008j | 0.0022±0.0007g | 0.0055±0.0012f | 0.0274±0.0051jk |
| *Bashania fargesii* | 0.0605±0.0078jk | 0.0073±0.0012ghi | 0.0447±0.0092ij | 0.0017±0.0006i | 0.0011±0.0003jk | 0.0028±0.0009hi | 0.0034±0.0009kl | 0.0025±0.0007gh | 0.0059±0.0015gh | 0.0335±0.0073jk |
| *Pseudosasa japonica* | 0.0450±0.0073b | 0.0052±0.0007a | 0.0291±0.0044a | 0.0009±0.0002a | 0.0005±0.0002a | 0.0014±0.0003a | 0.0025±0.0007a | 0.0015±0.0004a | 0.0040±0.001a | 0.0221±0.0035a |
| *Sasa fortunei* | 0.1189±0.0268m | 0.0150±0.0042k | 0.2158±0.0883l | 0.0062±0.0029kl | 0.0043±0.0022lmn | 0.0105±0.0048lm | 0.0172±0.0079opqrs | 0.0128±0.0059jkl | 0.0299±0.0131klmn | 0.1641±0.0657n |
| *Sasa pygmaea* | 0.1157±0.0166jk | 0.0178±0.0030gh | 0.2171±0.0491j | 0.0071±0.0025h | 0.0046±0.0017h | 0.0117±0.0039g | 0.0189±0.0056ij | 0.0126±0.0036gh | 0.0314±0.0085f | 0.1635±0.0382kl |
| *Qiongzhuea tumidinoda* | 0.1219±0.0129pq | 0.0101±0.0012pq | 0.0854±0.0197q | 0.0035±0.0009no | 0.0024±0.0006pq | 0.0059±0.0012pqr | 0.0091±0.0027tu | 0.0059±0.0015no | 0.0149±0.0036pq | 0.0606±0.0167r |
| *Indocalamus decorus* | 0.0276±0.0050q | 0.0048±0.0007r | 0.0143±0.0035q | 0.0005±0.0001q | 0.0003±0.0001q | 0.0009±0.0002s | 0.0011±0.0003u | 0.0008±0.0002o | 0.0019±0.0005q | 0.0108±0.0027s |
| *Indocalamus tessellatus* | 0.0399±0.0069l | 0.0062±0.0006kl | 0.0268±0.0062l | 0.0009±0.0003lm | 0.0006±0.0002mno | 0.0016±0.0005lmn | 0.0023±0.0006lmn | 0.0014±0.0004jk | 0.0038±0.0009ij | 0.0200±0.0050n |
| *Pleioblastus amarus* | 0.0659±0.0099q | 0.0079±0.0016pq | 0.0581±0.0159nop | 0.0021±0.0008mn | 0.0013±0.0006p | 0.0033±0.0013op | 0.0051±0.0019qrs | 0.0037±0.0016kl | 0.0088±0.0033lmno | 0.0432±0.0117opqr |
| *Phyllostachys aurea* | 0.0839±0.0082m | 0.0127±0.0016no | 0.1030±0.0247lm | 0.0034±0.0010kl | 0.0024±0.0008no | 0.0059±0.0016lm | 0.0081±0.0024nopq | 0.0056±0.0014klm | 0.0136±0.0034jklm | 0.0776±0.0205no |
| *Phyllostachys aureosulcata* f. *Spectabilis* | 0.0795±0.0122no | 0.0114±0.0026o | 0.0882±0.0272mnop | 0.0029±0.0010k | 0.0020±0.0007no | 0.0049±0.0015lm | 0.0073±0.0024opqr | 0.0049±0.0018jkl | 0.0122±0.0039klmn | 0.0659±0.0216pqr |
| *Phyllostachys bissetii* | 0.0855±0.0116op | 0.0132±0.0024p | 0.1089±0.0209mnop | 0.0035±0.0011no | 0.0023±0.0008p | 0.0058±0.0017pqr | 0.0088±0.0021st | 0.0060±0.0016mn | 0.0148±0.0033op | 0.0817±0.0154opqr |
| *Phyllostachys edulis* | 0.1305±0.0539l | 0.0157±0.0037n | 0.1520±0.0905l | 0.0058±0.0038mno | 0.0042±0.0030o | 0.0100±0.0066nop | 0.0154±0.0103mno | 0.0099±0.0063klm | 0.0253±0.0162jklm | 0.1090±0.0654n |
| *Phyllostachys mannii* | 0.0895±0.0296d | 0.0121±0.0038e | 0.1156±0.0731e | 0.0039±0.0024e | 0.0026±0.0015ef | 0.0065±0.0037d | 0.0086±0.0058f | 0.0060±0.004e | 0.0147±0.0096d | 0.0879±0.0577de |
| *Phyllostachys nigra* | 0.1024±0.0159o | 0.0135±0.0022r | 0.1175±0.0320op | 0.0038±0.0014p | 0.0027±0.0010pq | 0.0065±0.0023s | 0.0107±0.0034pqrs | 0.0073±0.0028no | 0.0180±0.0059no | 0.0873±0.0250qr |
| *Phyllostachys nigra* var. *henonis* | 0.0925±0.0169m | 0.0123±0.0019lm | 0.1210±0.0239k | 0.0038±0.0009h | 0.0025±0.0007jk | 0.0063±0.0012h | 0.0098±0.0026lm | 0.0066±0.0019i | 0.0163±0.0040hi | 0.0908±0.0196m |
| *Phyllostachys vivax* f. *aureocaulis* | 0.0849±0.0159l | 0.0100±0.0021ghi | 0.0905±0.0291h | 0.0030±0.0013g | 0.0021±0.0009g | 0.0050±0.0021f | 0.0075±0.003g | 0.0047±0.0019f | 0.0123±0.0046e | 0.0676±0.0218hi |

Means with the same letters in each column was not significantly different (P < 0.05). Means with the different letters in each column was significantly different (P≥0.05).

Table S3 The goodness of fit (R^2^) of each model in the regression calculation process of all indicators

| Curve model | Leaf vein density | Leaf length | Leaf width | Adaxial epidermis thickness | Skin thickness | Leaf length/  leaf thickness | Leaf width/  leaf thickness | Adaxial epidermis thickness/  leaf thickness | Epidermal thickness/  leaf thickness | Leaf length/  leaf area | Leaf width/  leaf area | Adaxial cuticle thickness/  leaf area | Cuticle thickness/  leaf area |
| --- | --- | --- | --- | --- | --- | --- | --- | --- | --- | --- | --- | --- | --- |
| Linear equation | 0.133 | 0.341 | 0.328 | 0.075 | 0.035 | 0.262 | 0.31 | 0.056 | 0.017 | 0.193 | 0.156 | 0.128 | 0.119 |
| Logarithmic curve equation | 0.173 | 0.275 | 0.272 | 0.061 | 0.025 | 0.21 | 0.247 | 0.046 | 0.009 | 0.283 | 0.27 | 0.344 | 0.332 |
| Inverse function curve equation | 0.207 | 0.172 | 0.19 | 0.047 | 0.016 | 0.142 | 0.165 | 0.037 | 0.003 | 0.342 | 0.346 | 0.447 | 0.438 |
| Quadratic curve equation | 0.284 | 0.374 | 0.354 | 0.106 | 0.101 | 0.286 | 0.326 | 0.099 | 0.164 | 0.293 | 0.297 | 0.264 | 0.257 |
| Cubic curve equation | 0.29 | 0.404 | 0.357 | 0.107 | 0.102 | 0.293 | 0.353 | 0.097 | 0.157 | 0.335 | 0.34 | 0.387 | 0.378 |
| Composite curve equation | 0.122 | 0.317 | 0.296 | 0.081 | 0.039 | 0.247 | 0.281 | 0.064 | 0.023 | 0.177 | 0.147 | 0.119 | 0.111 |
| Power function curve | 0.159 | 0.259 | 0.248 | 0.067 | 0.028 | 0.198 | 0.225 | 0.055 | 0.013 | 0.258 | 0.255 | 0.32 | 0.308 |
| S-shaped curve equation | 0.191 | 0.163 | 0.174 | 0.05 | 0.018 | 0.133 | 0.149 | 0.045 | 0.006 | 0.309 | 0.322 | 0.4 | 0.395 |
| Growth curve equation | 0.122 | 0.317 | 0.296 | 0.081 | 0.039 | 0.247 | 0.281 | 0.064 | 0.023 | 0.177 | 0.147 | 0.119 | 0.111 |
| exponential curve equation | 0.122 | 0.317 | 0.296 | 0.081 | 0.039 | 0.247 | 0.281 | 0.064 | 0.023 | 0.177 | 0.147 | 0.119 | 0.111 |
| Logistic curve equation | 0.122 | 0.317 | 0.296 | 0.081 | 0.039 | 0.247 | 0.281 | 0.064 | 0.023 | 0.177 | 0.147 | 0.119 | 0.111 |

Table S4 Eigenvalues and cumulative contribution rates of principal components.

| Principal components | Eigenvalues | Contribution rates (%) | Cumulative contribution rates (%) |
| --- | --- | --- | --- |
| Factor 1 | 7.203 | 55.407 | 55.407 |
| Factor 2 | 2.533 | 19.483 | 74.889 |
| Factor 3 | 1.455 | 11.191 | 86.08 |
| Factor 4 | 1.155 | 8.884 | 94.964 |
| Factor 5 | 0.437 | 3.359 | 98.323 |
| Factor 6 | 0.118 | 0.911 | 99.234 |
| Factor 7 | 0.039 | 0.303 | 99.537 |
| Factor 8 | 0.031 | 0.238 | 99.774 |
| Factor 9 | 0.02 | 0.154 | 99.928 |
| Factor 10 | 0.007 | 0.05 | 99.979 |
| Factor 11 | 0.002 | 0.016 | 99.995 |
| Factor 12 | 0 | 0.003 | 99.998 |
| Factor 13 | 0 | 0.002 | 100 |

Table S5 Principal component score coefficient matrix.

| Leaf morphology and anatomical indicators | Principal components | | | |
| --- | --- | --- | --- | --- |
|  | Factor 1 | Factor 2 | Factor 3 | Factor 4 |
| Leaf vein density | 0.094 | 0.023 | 0.086 | -0.403 |
| Leaf length | -0.129 | 0.07 | -0.016 | 0.186 |
| Leaf width | -0.12 | 0.046 | -0.121 | 0.328 |
| Adaxial epidermis thickness | 0.039 | 0.344 | -0.264 | 0.078 |
| Skin thickness | 0.039 | 0.325 | -0.323 | 0.09 |
| Leaf length/leaf thickness | -0.115 | 0.002 | 0.348 | 0.146 |
| Leaf width/leaf thickness | -0.119 | 0.003 | 0.149 | 0.336 |
| Adaxial epidermis thickness/leaf thickness | 0.064 | 0.277 | 0.363 | 0.067 |
| Epidermal thickness/leaf thickness | 0.067 | 0.227 | 0.446 | 0.06 |
| Leaf length/leaf area | 0.128 | -0.1 | 0.116 | 0.097 |
| Leaf width/leaf area | 0.123 | -0.097 | -0.027 | 0.314 |
| Adaxial cuticle thickness/leaf area | 0.12 | -0.089 | -0.044 | 0.38 |
| Cuticle thickness/leaf area | 0.119 | -0.088 | -0.029 | 0.391 |

Table S6 The goodness of fit (R^2^) of each model in the regression calculation process of all factors

| Curve model | Factor 1 | Factor 2 | Factor 3 | Factor 4 |
| --- | --- | --- | --- | --- |
| Linear equation | 0.317 | 0.417 | 0.349 | 0.311 |
| Logarithmic curve equation |  | 0.404 |  |  |
| Inverse function curve equation | 0.003 | 0.383 | 0.159 | 0.027 |
| Quadratic curve equation | 0.355 | 0.42 | 0.428 | 0.341 |
| Cubic curve equation | 0.376 | 0.42 | 0.469 | 0.35 |
| Composite curve equation | 0.295 | 0.396 | 0.323 | 0.291 |
| Power function curve |  | 0.387 |  |  |
| S-shaped curve equation | 0.003 | 0.369 | 0.159 | 0.024 |
| Growth curve equation | 0.295 | 0.396 | 0.323 | 0.291 |
| exponential curve equation | 0.295 | 0.396 | 0.323 | 0.291 |
| Logistic curve equation | 0.295 | 0.396 | 0.323 | 0.291 |

Table S7 Eigenvalues and cumulative contribution rates of principal components.

| Principal components | Eigenvalues | Contribution rates (%) | Cumulative contribution rates (%) |
| --- | --- | --- | --- |
| Factor1 | 6.946 | 63.149 | 63.149 |
| Factor2 | 1.534 | 13.948 | 77.097 |
| Factor3 | 1.098 | 9.986 | 87.083 |
| Factor4 | 0.815 | 7.412 | 94.495 |
| Factor5 | 0.424 | 3.851 | 98.345 |
| Factor6 | 0.114 | 1.039 | 99.384 |
| Factor7 | 0.033 | 0.299 | 99.683 |
| Factor8 | 0.025 | 0.231 | 99.914 |
| Factor9 | 0.007 | 0.062 | 99.976 |
| Factor10 | 0.002 | 0.02 | 99.996 |
| Factor11 | 0 | 0.004 | 100 |

Table S8 Component score coefficient matrix.

| Leaf morphology and anatomical indicators | Principal components | | |
| --- | --- | --- | --- |
|  | Factor 1 | Factor 2 | Factor 3 |
| Leaf vein density | 0.094 | 0.023 | 0.086 |
| Leaf length | -0.129 | 0.07 | -0.016 |
| Leaf width | -0.12 | 0.046 | -0.121 |
| Adaxial epidermis thickness | 0.039 | 0.344 | -0.264 |
| Skin thickness | 0.039 | 0.325 | -0.323 |
| Leaf length/leaf thickness | -0.115 | 0.002 | 0.348 |
| Leaf width/leaf thickness | -0.119 | 0.003 | 0.149 |
| Adaxial epidermis thickness/leaf thickness | 0.064 | 0.277 | 0.363 |
| Epidermal thickness/leaf thickness | 0.067 | 0.227 | 0.446 |
| Leaf length/leaf area | 0.128 | -0.1 | 0.116 |
| Leaf width/leaf area | 0.123 | -0.097 | -0.027 |
| Adaxial cuticle thickness/leaf area | 0.12 | -0.089 | -0.044 |
| Cuticle thickness/leaf area | 0.119 | -0.088 | -0.029 |

Table S9 The goodness of fit (R2) of each model in the regression calculation process of all factors

| Curve model | Factor 1 | Factor 2 | Factor 3 |
| --- | --- | --- | --- |
| Linear equation | 0.337 | 0 | 0.347 |
| Logarithmic curve equation | 2 | 0.001 | 2 |
| Inverse function curve equation | 0.009 | 0.003 | 0.034 |
| Quadratic curve equation | 0.371 | 0.086 | 0.374 |
| Cubic curve equation | 0.4 | 0.082 | 0.399 |
| Composite curve equation | 0.312 | 0 | 0.321 |
| Power function curve | 2 | 0 | 2 |
| S-shaped curve equation |  | 0.002 | 0.032 |
| Growth curve equation | 0.312 | 0 | 0.321 |
| exponential curve equation | 0.312 | 0 | 0.321 |
| Logistic curve equation | 0.312 | 0 | 0.321 |
