# Supplementary figures and images for: Overwintering performance of bamboo leaves, and establishment of mathematical model for the distribution and introduction prediction of bamboos
Source: Front Plant Sci. 2023 Sep 8;14:1255033. doi: 10.3389/fpls.2023.1255033 (PMC10515091; doi:10.3389/fpls.2023.1255033)

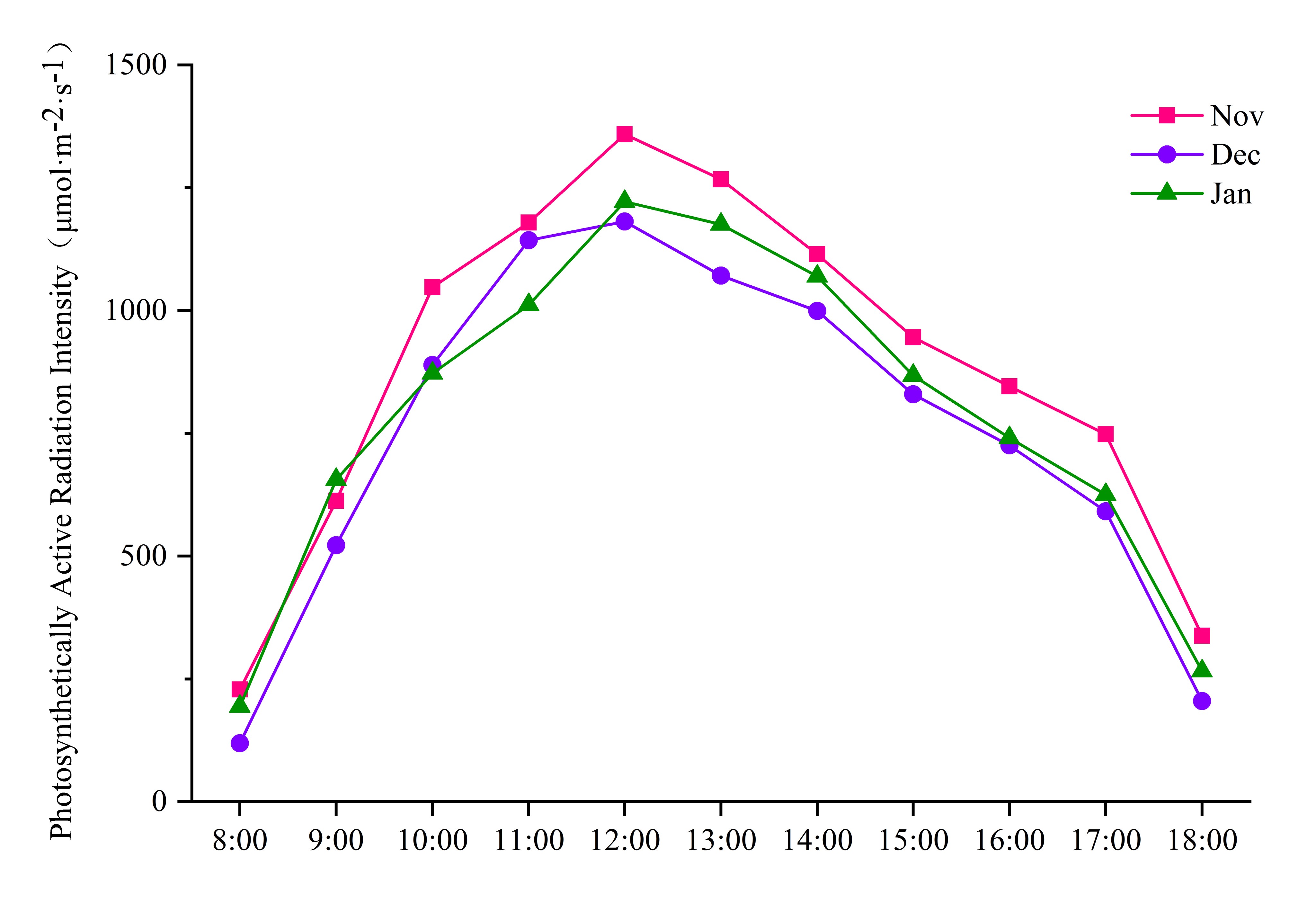

Supplement: Supplementary Figure 1 — Daily changes of photosynthetically active radiation intensity in different months. [file Image_1.jpeg]

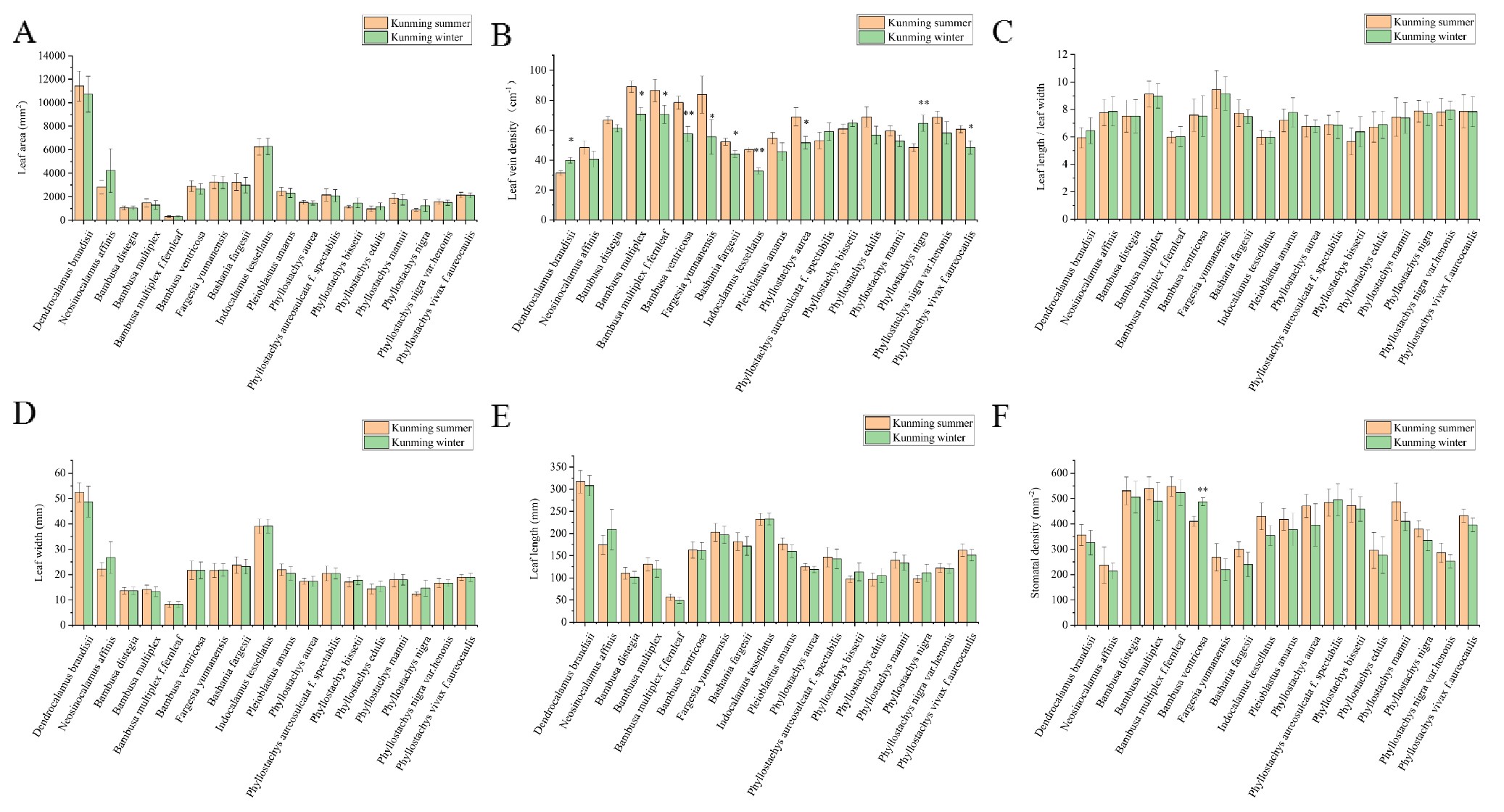

Supplement: Supplementary Figure 3 — Changes of leaf morphological indicators of 18 bamboo species with different seasons. (A) Leaf area. (B) Leaf vein density. (C) Stomatal density. (D) Leaf length. (E) Leaf width. (F) Leaf length/leaf width. [file Image_3.jpeg]

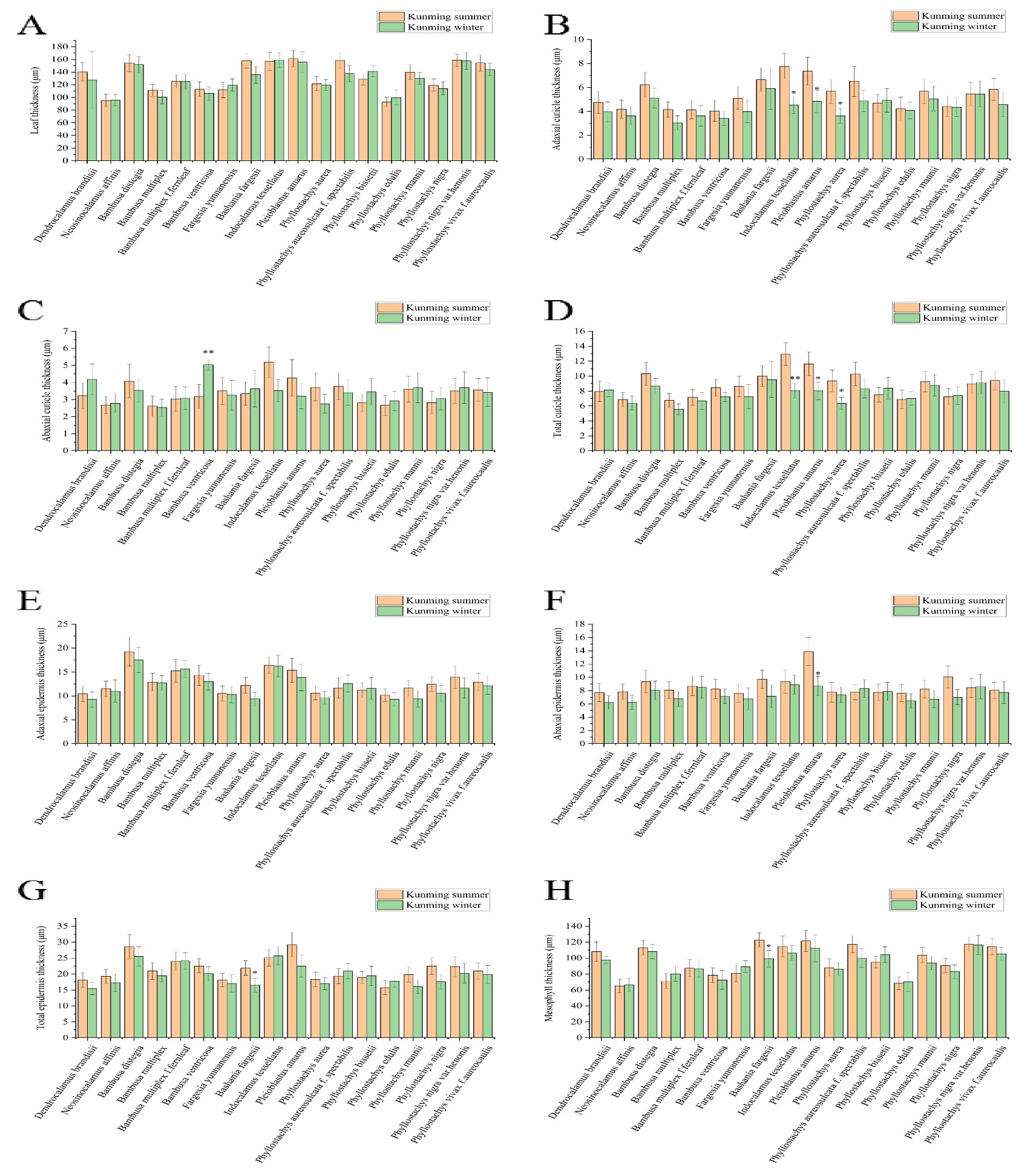

Supplement: Supplementary Figure 4 — Changes of leaf anatomical indicators of 18 bamboo species with different seasons. (A) Leaf thickness. (B) Adaxial cuticle thickness. (C) Abaxial cuticle thickness. (D) Total cuticle thickness. (E) Adaxial epidermis thickness. (F) Abaxial epidermis thickness. (G) Total epidermis thickness. (H) Mesophyll thickness. [file Image_4.jpeg]

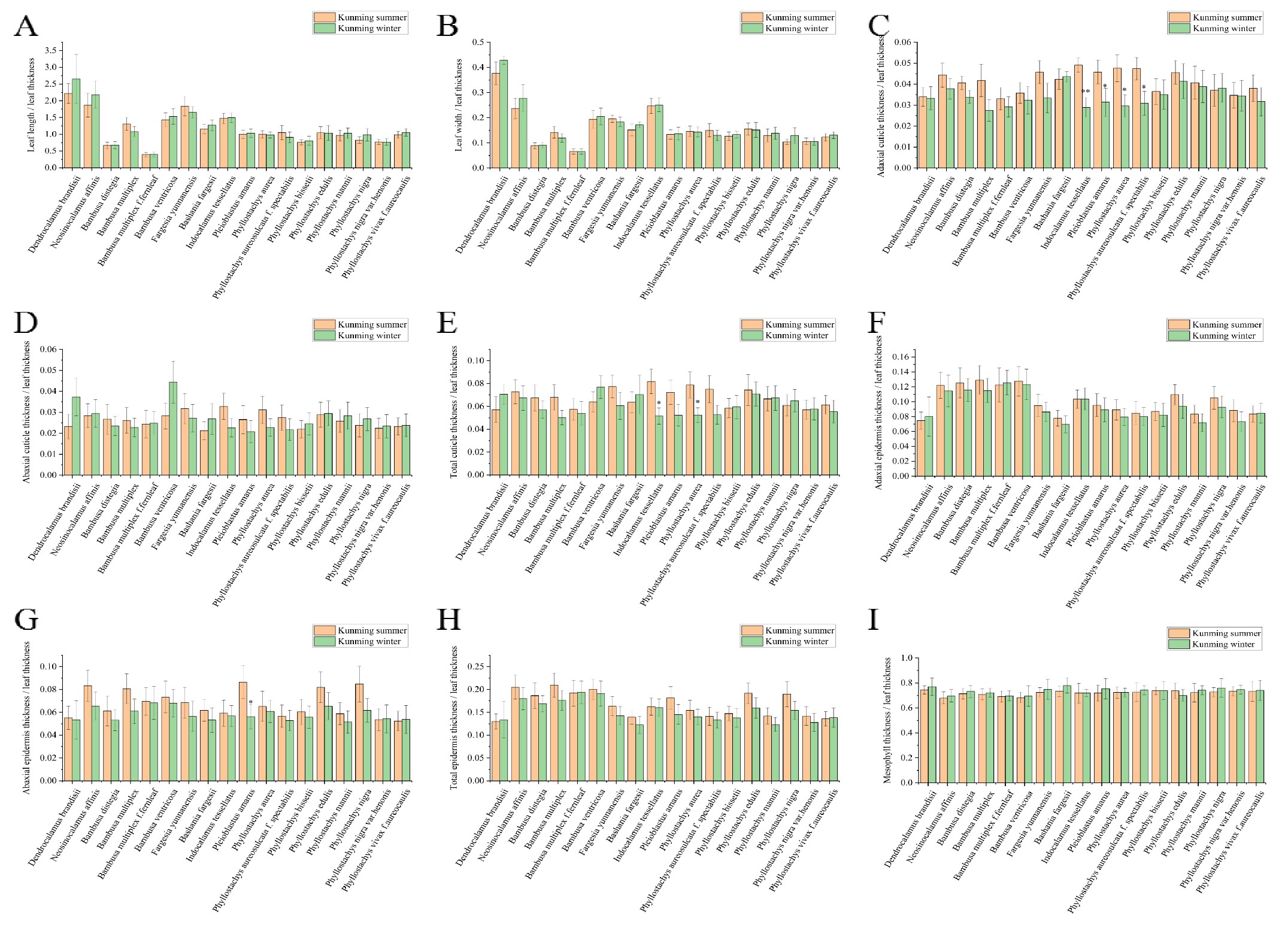

Supplement: Supplementary Figure 5 — The changes of ratios of morphological and anatomical indicators to leaf thickness of 18 bamboo species with different seasons. (A) Leaf length/leaf thickness. (B) Leaf width/leaf thickness. (C) Adaxial cuticle thickness/leaf thickness. (D) Abaxial cuticle thickness/leaf thickness. (E) Total cuticle thickness/leaf thickness. (F) Adaxial epidermis thickness/leaf thickness. (G) Abaxial epidermis thickness/leaf thickness. (H) Total epidermis thickness/leaf thickness. (I) Mesophyll thickness/leaf thickness. [file Image_5.jpeg]

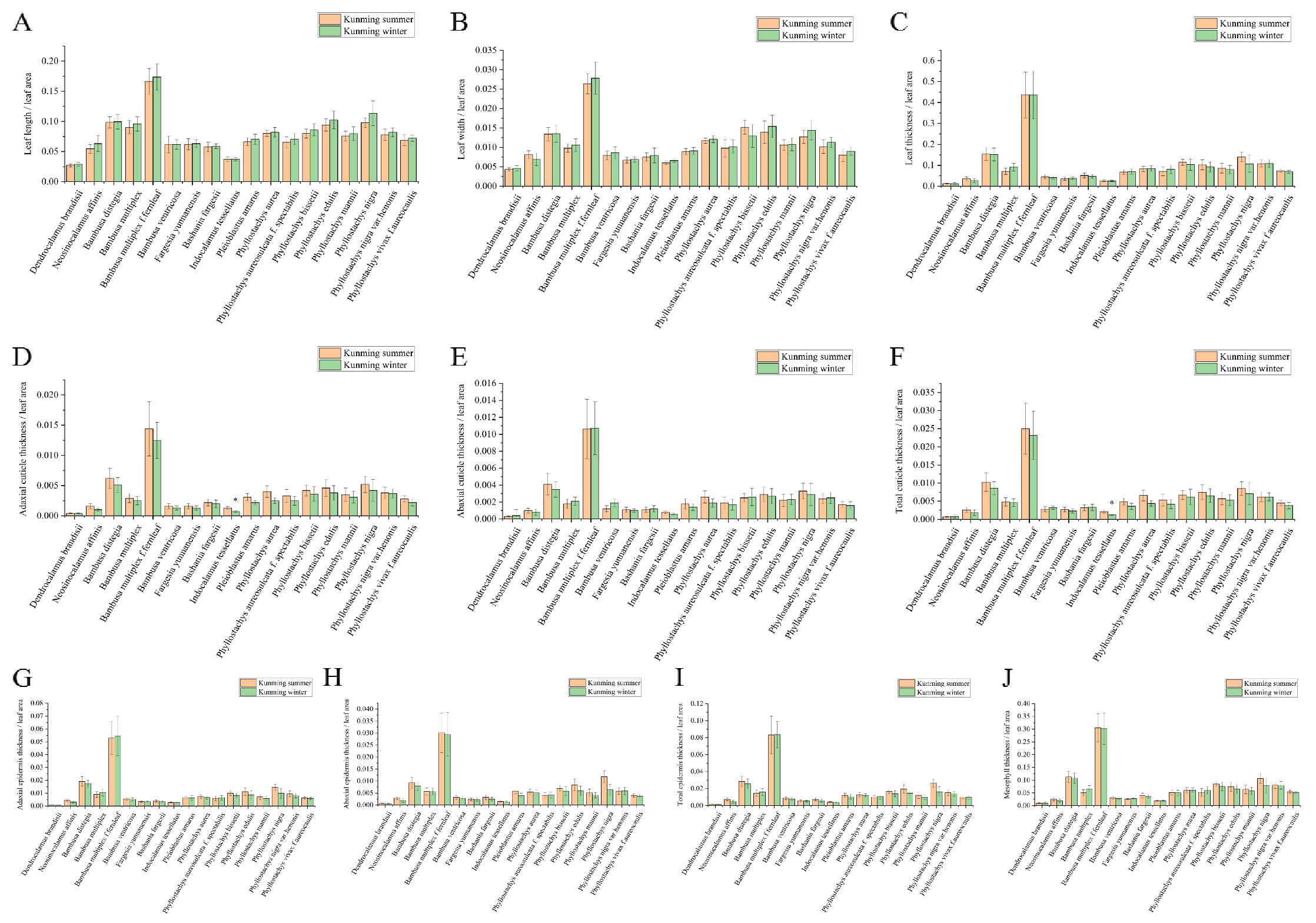

Supplement: Supplementary Figure 6 — The changes of ratios of leaf morphological and anatomical indicators to leaf area of 18 bamboo species with different seasons. (B) Leaf width/leaf area. (C) Leaf thickness/leaf area. (D) Adaxial cuticle thickness/leaf area. (E) Abaxial cuticle thickness/leaf area. (F) Total cuticle thickness/leaf area. (G) Adaxial epidermis thickness/leaf area. (H) Abaxial epidermis thickness/leaf area. (I) Total epidermis thickness/leaf area. (J) Mesophyll thickness/leaf area. [file Image_6.jpeg]

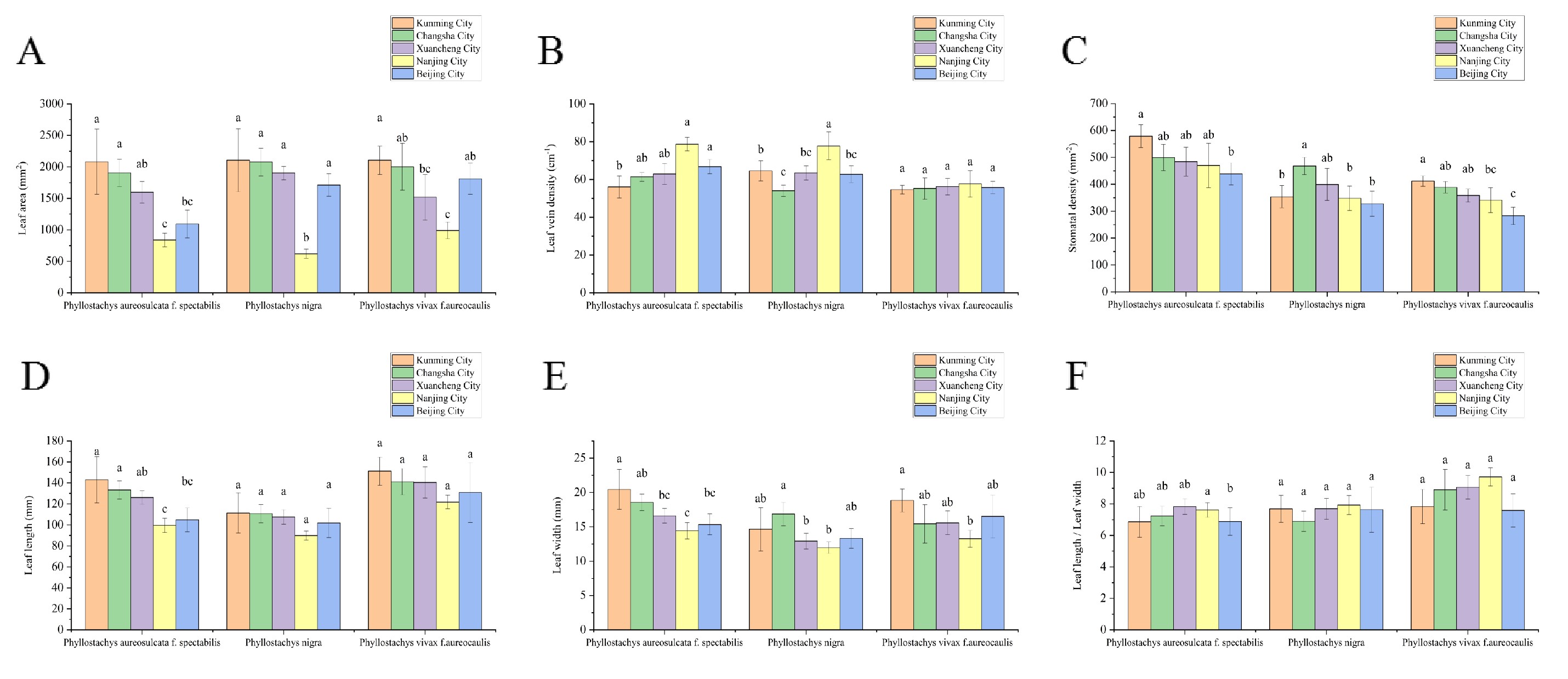

Supplement: Supplementary Figure 7 — Changes of morphological indicators of bamboo leaves of three bamboo species with different regions. (A) Leaf area. (B) Leaf vein density. (C) Stomatal density. (D) Leaf length. (E) Leaf width. (F) Leaf length/leaf width. [file Image_7.jpeg]

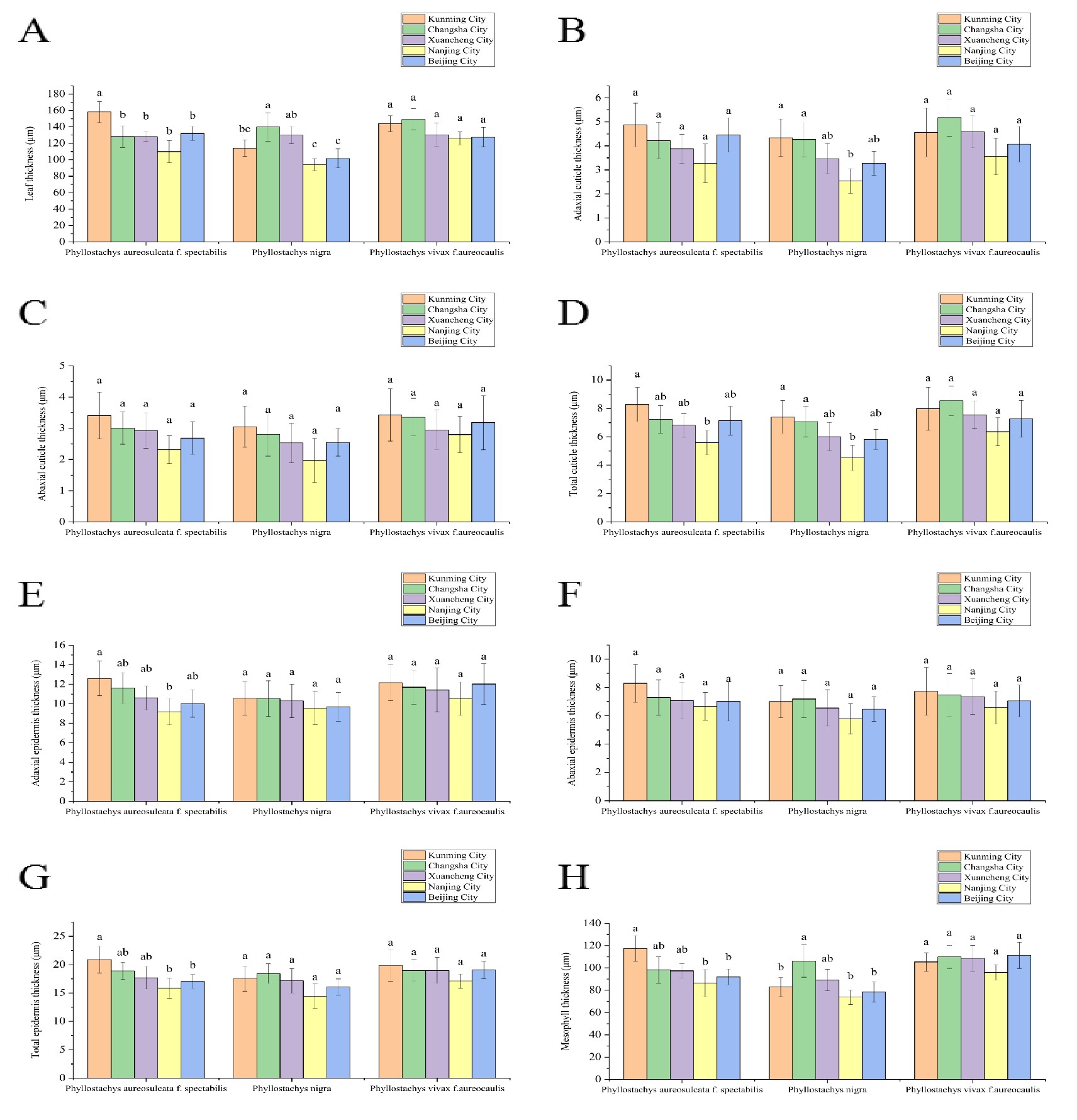

Supplement: Supplementary Figure 8 — Changes of anatomical indicators of bamboo leaves of three bamboo species with different regions. (A) Leaf thickness. (B) Adaxial cuticle thickness. (C) Abaxial cuticle thickness. (D) Total cuticle thickness. (E) Adaxial epidermis thickness. (F) Abaxial epidermis thickness. (G) Total epidermis thickness. (H) Mesophyll thickness. [file Image_8.jpeg]

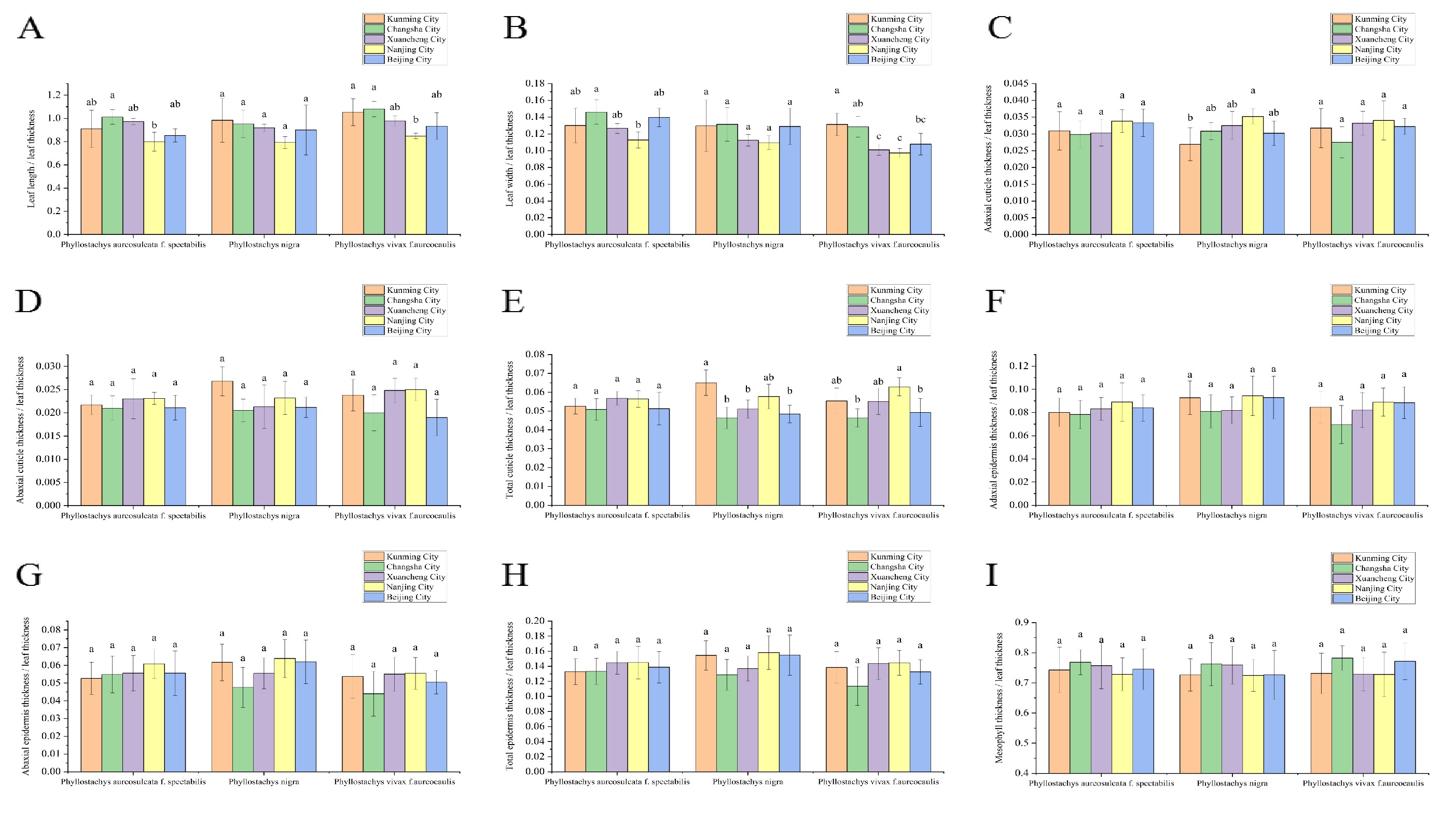

Supplement: Supplementary Figure 9 — Changes of ratios of leaf morphological and anatomical indicators to leaf thickness of three bamboo species with different regions. (A) Leaf length/leaf thickness. (B) Leaf width/leaf thickness. (C) Adaxial cuticle thickness/leaf thickness. (D) Abaxial cuticle thickness/leaf thickness. (E) Total cuticle thickness/leaf thickness. (F) Adaxial epidermis thickness/leaf thickness. (G) Abaxial epidermis thickness/leaf thickness. (H) Total epidermis thickness/leaf thickness. (I) Mesophyll thickness/leaf thickness. [file Image_9.jpeg]

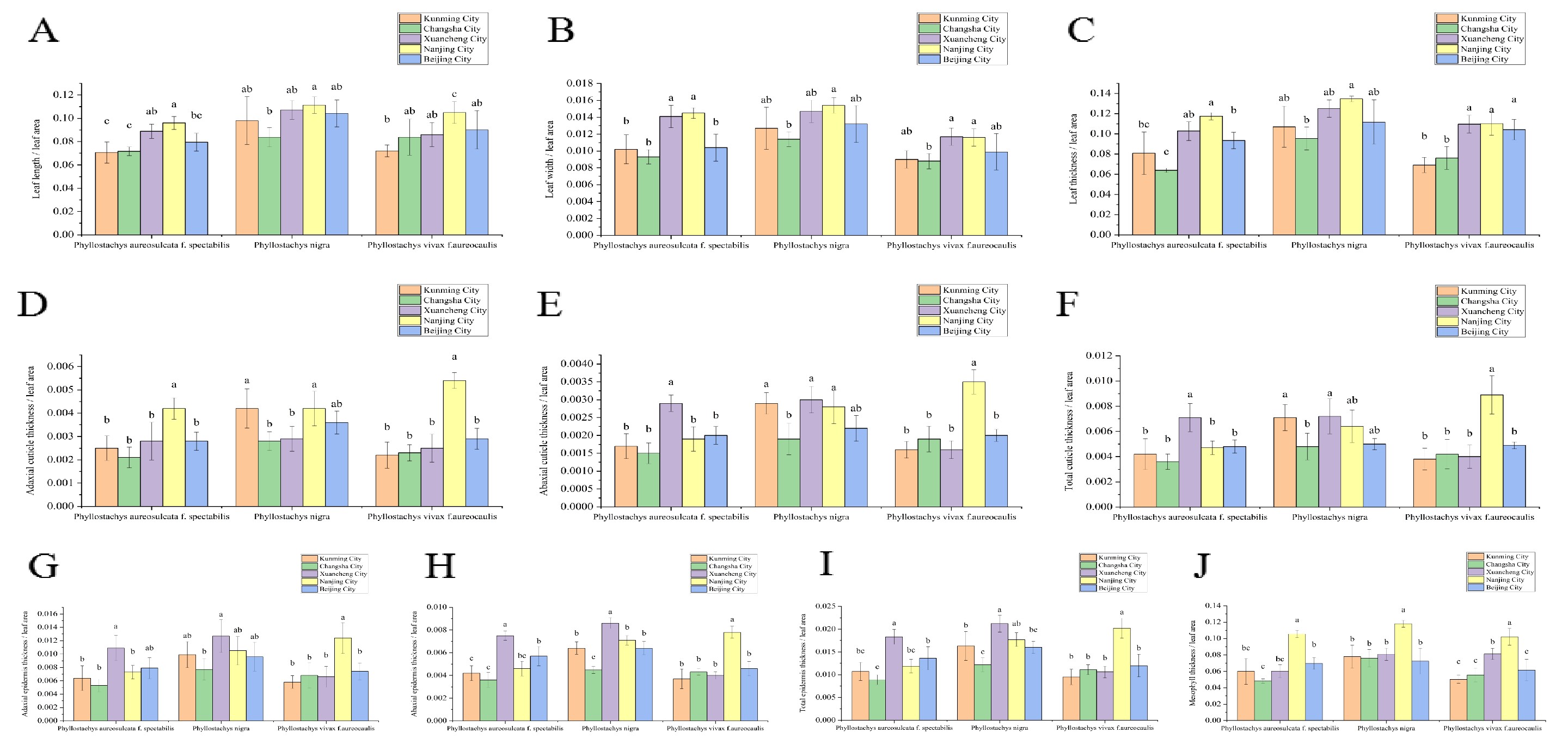

Supplement: Supplementary Figure 10 — Changes of ratios of morphological and anatomical indicators to leaf area of three bamboo species with different regions. (A) Leaf length/leaf area. (B) Leaf width/leaf area. (C) Leaf thickness/leaf area. (D) Adaxial cuticle thickness/leaf area. (E) Abaxial cuticle thickness/leaf area. (F) Total cuticle thickness/leaf area. (G) Adaxial epidermis thickness/leaf area. (H) Abaxial epidermis thickness/leaf area. (I) Total epidermis thickness/leaf area. (J) Mesophyll thickness/leaf area. [file Image_10.jpeg]
